# En Route to Stabilized Compact Conformations of Single-Chain Polymeric Nanoparticles in Complex Media

**DOI:** 10.1021/acs.macromol.2c00930

**Published:** 2022-07-13

**Authors:** Stefan Wijker, Linlin Deng, Fabian Eisenreich, Ilja K. Voets, Anja R. A. Palmans

**Affiliations:** † Institute for Complex Molecular Systems, Laboratory of Macromolecular and Organic Chemistry, Eindhoven University of Technology, P.O. Box 513, 5600 MB Eindhoven, The Netherlands; ‡ Laboratory of Self-Organizing Soft Matter, Department of Chemical Engineering and Chemistry, Institute for Complex Molecular Systems, Eindhoven University of Technology, P.O. Box 513, 5600 MB Eindhoven, The Netherlands

## Abstract

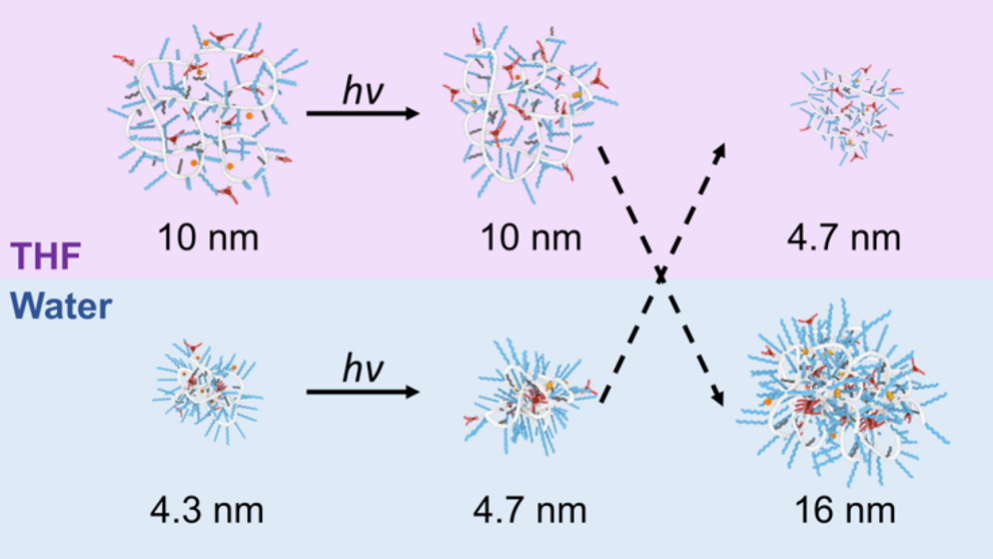

Precise control over the folding pathways of polypeptides using a combination of noncovalent and covalent interactions has evolved into a wide range of functional proteins with a perfectly defined 3D conformation. Inspired hereby, we develop a series of amphiphilic copolymers designed to form compact, stable, and structured single-chain polymeric nanoparticles (SCPNs) of defined size, even in competitive conditions. The SCPNs are formed through a combination of noncovalent interactions (hydrophobic and hydrogen-bonding interactions) and covalent intramolecular cross-linking using a light-induced [2 + 2] cycloaddition. By comparing different self-assembly pathways of the nanoparticles, we show that, like for proteins in nature, the order of events matters. When covalent cross-links are formed prior to the folding via hydrophobic and supramolecular interactions, larger particles with less structured interiors are formed. In contrast, when the copolymers first fold via hydrophobic and hydrogen-bonding interactions into compact conformations, followed by covalent cross-links, good control over the size of the SCPNs and microstructure of the hydrophobic interior is achieved. Such a structured SCPN can stabilize the solvatochromic dye benzene-1,3,5-tricarboxamide–Nile Red via molecular recognition for short periods of time in complex media, while showing slow exchange dynamics with the surrounding complex media at longer time scales. The SCPNs show good biocompatibility with cells and can carry cargo into the lysosomal compartments of the cells. Our study highlights the importance of control over the folding pathway in the design of stable SCPNs, which is an important step forward in their application as noncovalent drug or catalyst carriers in biological settings.

## Introduction

Single-chain polymeric nanoparticles have attracted significant interest as a result of their controllable size and their ability to adopt well-defined conformations in dilute solutions.^1−4^ Numerous examples using different chemistries have been evaluated to restrict the conformational freedom of single polymer chains in solution and thereby prepare SCPNs.^5−8^ The two main approaches are the use of (dynamic) covalent bonds for intramolecular cross-linking of single polymer chains and the use of noncovalent, supramolecular interactions to induce an intramolecular collapse. In covalent approaches, click chemistry,^9−14^ cycloadditions,^15,16^ free radical polymerization,^17−20^ dimerization reactions,^21,22^ and others^23−27^ have been applied, which irreversibly cross-link polymer chains. By use of dynamic, reversible covalent cross-links, such as disulfide bridges, imines, and Diels–Alder cycloaddition products, cross-links can be cleaved and re-formed under certain conditions.^28−31^ The second approach uses supramolecular interactions such as hydrogen bonds,^32−39^ π–π stacking interactions,^40,41^ host–guest interactions,^42−44^ or metal–ligand coordination chemistry.^45−47^ In water, purely hydrophobic interactions have also been explored to form SCPNs, as elegantly shown by the work of Morishima et al.^48−53^ as well as Terashima and Sawamoto et al.^54−58^ Combining hydrophobic with hydrogen-bonding interactions has permitted the collapse and concurrent folding of amphiphilic synthetic polymers into structured, compartmentalized nanoparticles.^33,59^ The folding of such amphiphilic systems into SCPNs is reminiscent of the way natural polypeptides fold into enzymes^60,61^ and has enabled mimicking some of the remarkable properties of enzymes, such as efficient catalysis in water.^62−67^

Combining a hydrophobic collapse with hydrogen-bond-driven folding using benzene-1,3,5-tricarboxamides (BTAs) has recently permitted us to access dynamic SCPN-based catalytic nanoreactors that function in complex media.^68^ However, the activity of the transition-metal complexes embedded in the SCPNs was reduced in complex biological media, which was attributed to interactions between the constituents of the media and the embedded transition-metal catalyst.^69^ As a cause of these undesired interactions, we proposed that the dynamic SCPNs adopt sparse, open conformations in solution rather than compact, globular particles.^70^ In fact, Pomposo et al. showed that almost all SCPNs adopt such sparse conformations.^71^ Sparse conformations create multiple small hydrophobic domains, which increases the likelihood of interactions between catalytic sites and the complex environment compared to compact conformations with a well-structured, hydrophobic interior. Indeed, small-angle neutron scattering (SANS) experiments on SCPNs that form predominantly through hydrogen-bond interactions between the BTA pendants corroborated the presence of elongated structures with multiple hydrophobic domains.^70^

With the aim to create stable, compact SCPNs, that is, SCPNs that maintain their size and structure in the presence of competing interactions, we started to explore different approaches to mitigate interactions between the SCPN and the surrounding media, taking inspiration from nature. Nature does not rely on a single interaction to fold polypeptides into stable, functional proteins but employs a combination of hydrophobic, hydrogen-bonding, and covalent chemistries to form stably folded structures. Moreover, the order in which these interactions/bonds are activated dictates the protein’s final structure and function. This was most strikingly illustrated by the Anfinsen experiment in 1962, which showed that hydrogen bonding followed by disulfide bridge formation was the correct folding pathway toward the native ribonuclease S species. Reversing the order of events led to a scrambled mixture of inactive proteins.^72^ Also in synthetic systems, the order of events matters. In a recent example, Zhang et al. showed the possibility of fixing various conformations of thermoresponsive *N-*isopropylacrylamide-based polymers in water. The thermoresponsive precursors adopted more open conformations at low temperatures and more collapsed states at high temperatures. Subsequent covalent cross-linking reactions at the different temperatures permitted the covalent locking of the obtained conformations and tuning the obtained SCPNs from loosely packed particles to collapsed globules.^73^ In addition, our group investigated approaches to control global conformations of polymers in organic media. Supramolecular BTA self-assembly as structuring graft was combined with reversible covalent dimerization of coumarin moieties. SCPNs were formed upon intramolecular coumarin dimerization in the absence of BTA assembly. However, in the presence of BTA self-assembly, coumarin dimerization occurred intermolecularly, resulting in the formation of large, irreversible aggregates.^74^ Thus, to stabilize polymer conformations in synthetic systems, like nature does with disulfide bridges, the order of activating bonds/interactions is important for the final structures and likely the stability of the SCPNs.

**Scheme 1:**
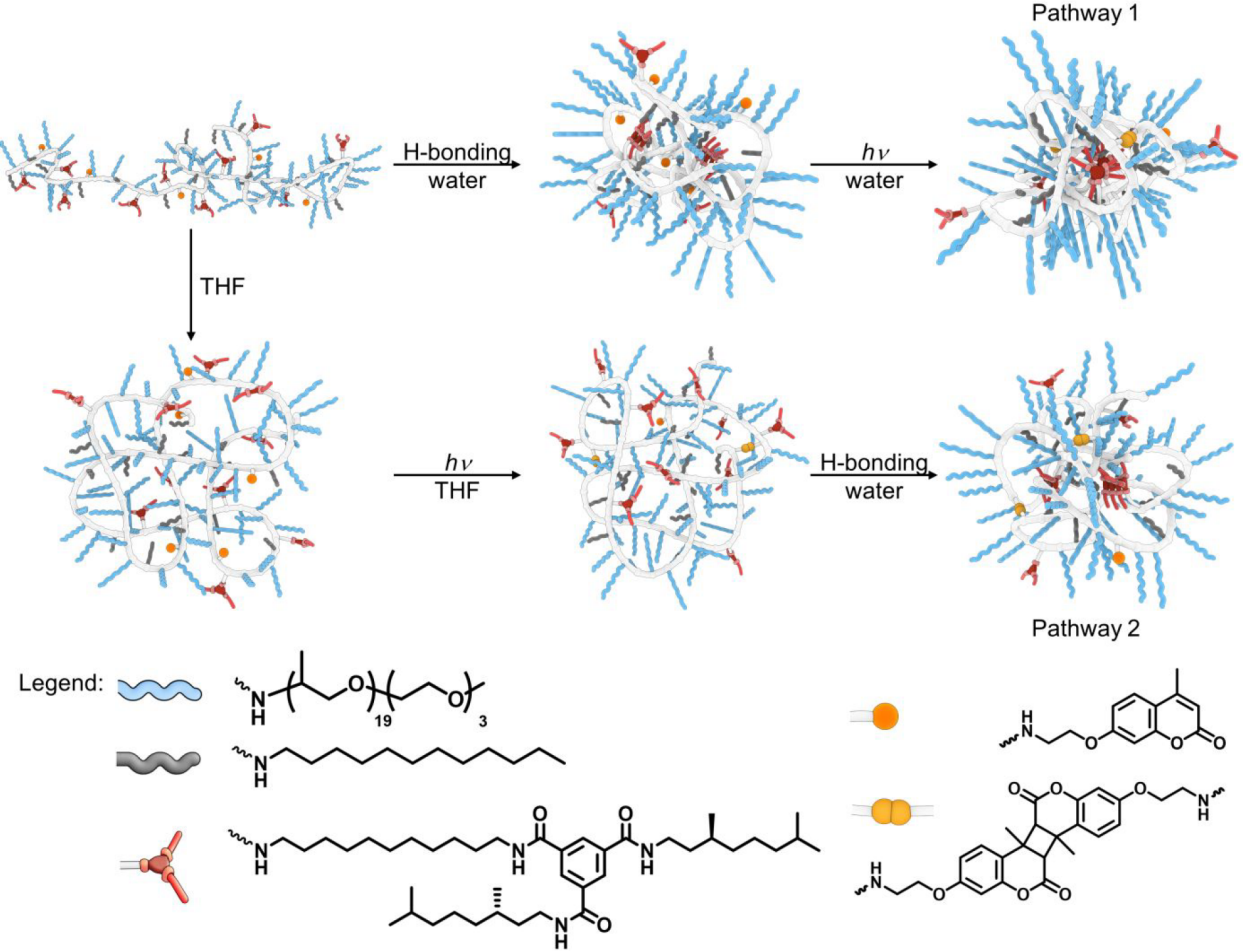
Representation of the Different Pathways Followed to Create Cross-Linked SCPNs In pathway 1 (top path, PW1), preorganization of the hydrophobic interior through hydrophobic collapse combined with BTA aggregation through triple hydrogen bonding is induced in water prior to the formation of covalent cross-links through photodimerization of the coumarin grafts. In pathway 2 (bottom path, PW2), preorganization of the hydrophobic interior is prevented in the good solvent THF, which disrupts hydrogen bonding. The covalent cross-links are formed through photodimerization of the coumarin grafts, upon which the polymers are redissolved in water to induce a collapse/folding of the polymer.

In this work, we investigate in detail the combination of noncovalent hydrophobic and hydrogen-bonding interactions with covalent cross-linking to access stable, structured, and compact SCPNs in water that retain their folded properties in complex media. Ideally, the SCPNs resist unfolding to keep the integrity of the hydrophobic interior in complex media and hereby prevent undesired interactions with biological molecules. To this end, we prepare a series of amphiphilic polymers randomly grafted with different functional groups. Hydrophilic Jeffamine@1000 grafts impart the polymer with sufficient water solubility, whereas hydrophobic dodecyl grafts aid the hydrophobic collapse and formation of more globular particles.^70,75,76^ The intramolecular folding is induced by the triple hydrogen-bond formation between BTA grafts into helical stacks, which results in a structured interior. Finally, coumarins are attached, which enable a [2 + 2] cycloaddition using UV-light, resulting in reversible covalent cross-linking.^29,30^ Because the Anfinsen experiment highlights the importance of controlling the folding pathway of enzymes to form the native, active species, we systematically study two distinct folding pathways of our synthetic system (Scheme 1). In pathway 1 (PW1), we fold the polymer in water into a SCPN and lock the compact conformation using covalent coumarin dimerization. Pathway 2 (PW2) reverses the order of events. Now, the coumarin grafts are first dimerized in a good solvent, THF, in which the polymer adopts a random coil-like conformation and no hydrogen bonding is present between the BTAs. Then, THF is removed, and the cross-linked polymer is dissolved in water, which induces the aggregation of BTAs. A combination of UV–vis absorbance, fluorescence, and circular dichroism (CD) spectroscopy, static and dynamic light scattering, and size exclusion chromatography (SEC) is used to investigate how both pathways affect the formation of the stable, structured, and compact SCPNs and how the SCPNs behave in complex media. Like in nature, PW1 leads to SCPNs with improved control over particle size, retention of particle size in complex media and more pronounced internal structure compared to PW2.

## Results and Discussion

### Synthesis and Characterization of Amphiphilic Random Copolymers

**Scheme 2:**
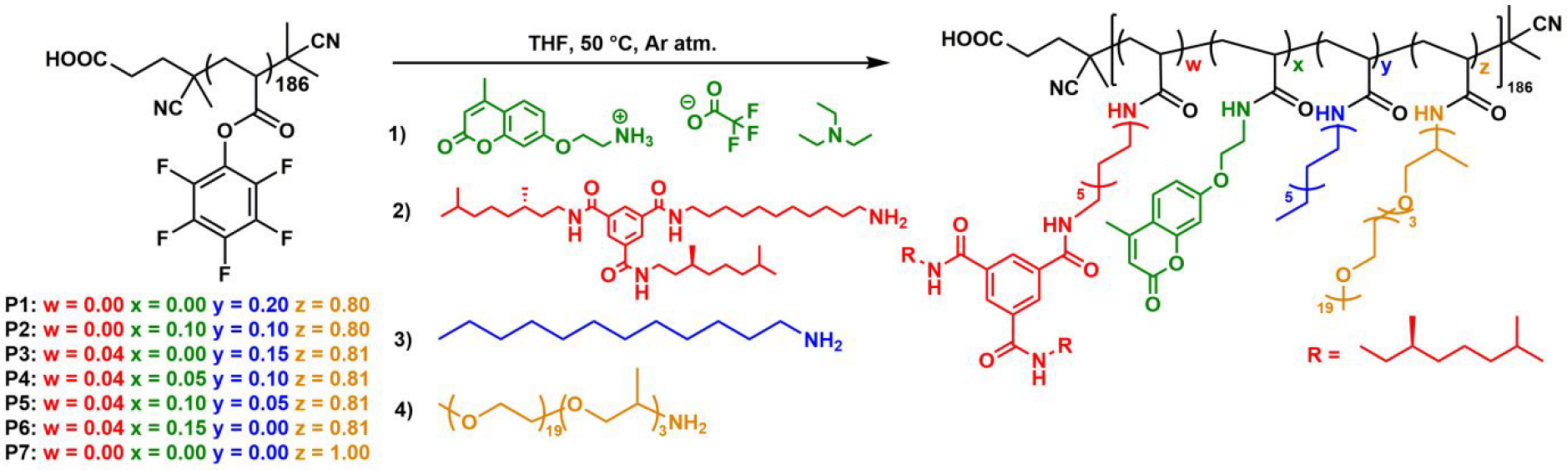
Synthetic Scheme of the Sequential Amine Postfunctionalization of PPFPA with DP = 186 to Afford Polymers **P1**–**P7** The incorporated graft ratios of each polymer were determined by ^19^F NMR spectroscopy.

Amphiphilic, random copolymers with a degree of polymerization (DP) of 200 were prepared via sequential amine postfunctionalization of poly(pentafluorophenyl acrylate) following a literature procedure.^77,78^ The general postfunctionalization procedure and graft incorporation ratios are shown in Scheme 2. Polymers **P1**–**P7** were prepared starting from the same prepolymer so that all polymers have the same molar mass dispersity and only differ in the ratios of different grafts. **P1** only contains Jeffamine@1000 and dodecyl grafts and serves as a model compound for SCPN formation in the absence of the cross-linkable coumarin grafts and the supramolecular unit BTA. **P2** incorporates 10% coumarin and 10% dodecyl grafts but no BTAs. **P3**–**P6** all incorporate 4% BTA grafts, with an increasing number of coumarin (0, 5, 10, and 15%, respectively) and decreasing number of dodecyl grafts (15, 10, 5, and 0%, respectively). **P7** only contains Jeffamine@1000 grafts. The full characterization of monomers and all polymers is given in the Supporting Information (Figures S1–S46).

Comparing the ^1^H NMR spectra of **P5** in CDCl_3_ (Figure S36) and D_2_O (Figure S47) provides a first indication of particle formation in water. Whereas the proton signals of the different grafts are well resolved in CDCl_3_, the signals corresponding to the BTA, coumarin, and dodecyl grafts as well as the polymer backbone are much weaker and broader in D_2_O. This indicates that these grafts are in a confined, restricted environment in D_2_O. In contrast, the signals of the Jeffamine@1000 grafts remain well resolved in both D_2_O and CDCl_3_. Additionally, fluorescence emission spectra were recorded of all polymer solutions after addition of the solvatochromic dye Nile Red, which is a well-known probe for hydrophobic compartments (Figure S48).^79^ The emission of Nile Red shows a blue-shift (λ = 631–640 nm) in the presence of **P1**–**P6** compared to pure water (λ = 656 nm), which is in line with similar amphiphilic polymers^37,62^ and indicates the presence of hydrophobic domains in water. The results from ^1^H NMR and fluorescence measurements agree well with the collapse/folding of the amphiphilic polymers in water, resulting in particles in which the hydrophobic grafts are located inside a hydrophobic pocket, shielded from the water phase by the hydrophilic Jeffamine@1000 grafts.

### Effect of Coumarin Incorporation on BTA Aggregation

The extent of helical stack formation by the chiral BTA grafts through triple hydrogen bonding in the different polymers was quantified by using circular dichroism (CD) spectroscopy.^59^ Only BTAs that are aggregated in a helical stack contribute to the CD effect.^80^ Because the BTA concentration is the same for all BTA-incorporating polymers, the magnitude of the CD effect, expressed as molar circular dichroism Δε, indicates which graft ratio leads to the largest amount of aggregated BTAs. **P4** in THF, a solvent that competes with hydrogen bonds and prevents BTA aggregation, does not show a CD effect (Figure S53).

**Figure 1:**
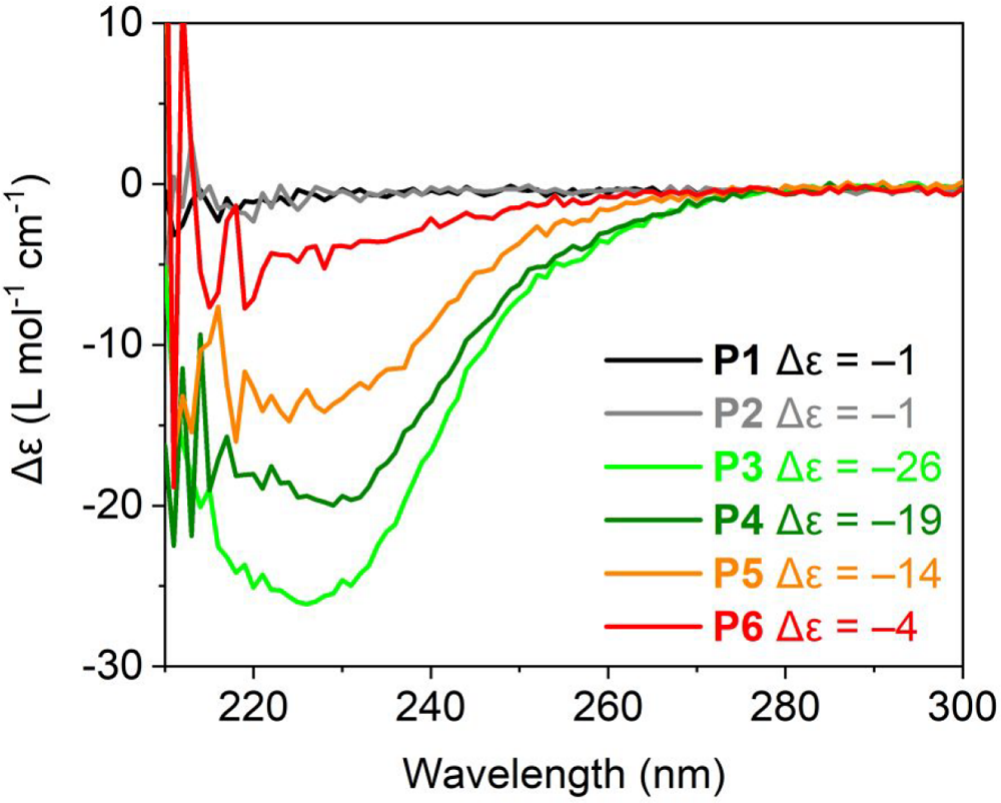
CD spectra of **P1**–**P6** in water at a concentration of 1 mg mL^–1^. *c*_BTA_ = 41 μM.

Figure 1 shows the CD spectra of aqueous solutions of **P1**–**P6**. No CD effect is observed for **P1** and **P2** as expected because there are no BTAs attached. **P3**–**P6**, all containing 4% BTAs, show negative CD effects centered around λ = 225 nm, indicative for the formation of a left-handed (*M*) helical BTA aggregate.^38,80,81^**P3** shows the largest Δε, indicating the highest degree of BTA stacking. Increasing the number of coumarin grafts and consequently lowering the dodecyl grafts, as with **P4**–**P6**, results in a 25–85% smaller value for Δε and thus less aggregation of the BTA grafts compared to **P3**. The CD heating and cooling curves of **P3**–**P6** (Figure S49) overlap well, reflecting the absence of hysteresis and reversibility of the BTA aggregation. All cooling curves have an isodesmic shape,^82^ indicating that the coumarin grafts do not influence the BTA aggregation mechanism. However, the differences in the values for Δε show that the replacement of dodecyl groups by coumarins reduces the degree of BTA aggregation. This is in line with previous results where higher contents of dodecyl chains were found to enhance BTA aggregation.^76^ In the remainder of our study, we therefore focus on **P4**, which shows the highest degree of BTA aggregation in the presence of coumarin grafts.

### Covalent Cross-Linking of Copolymers via UV-Light-Induced Coumarin Dimerization

The photodimerizations via a [2 + 2] cycloaddition of the coumarin grafts were performed at low polymer concentrations (1 mg mL^–1^) to promote intramolecular cross-linking over intermolecular cross-linking (see sections 4–6 in the Supporting Information for experimental details). For PW1, the photodimerization was performed in water, whereas for PW2, the photodimerization was performed in THF. All polymer solutions were irradiated with UV-light (λ_irr_ = 365 nm) at an intensity of 420 mW cm^–2^ for 6 h. The photodimerization process was monitored by following the decrease of the absorption band around λ = 322 nm, which is characteristic for the conversion of the coumarin monomer to its dimer.

**Figure 2:**
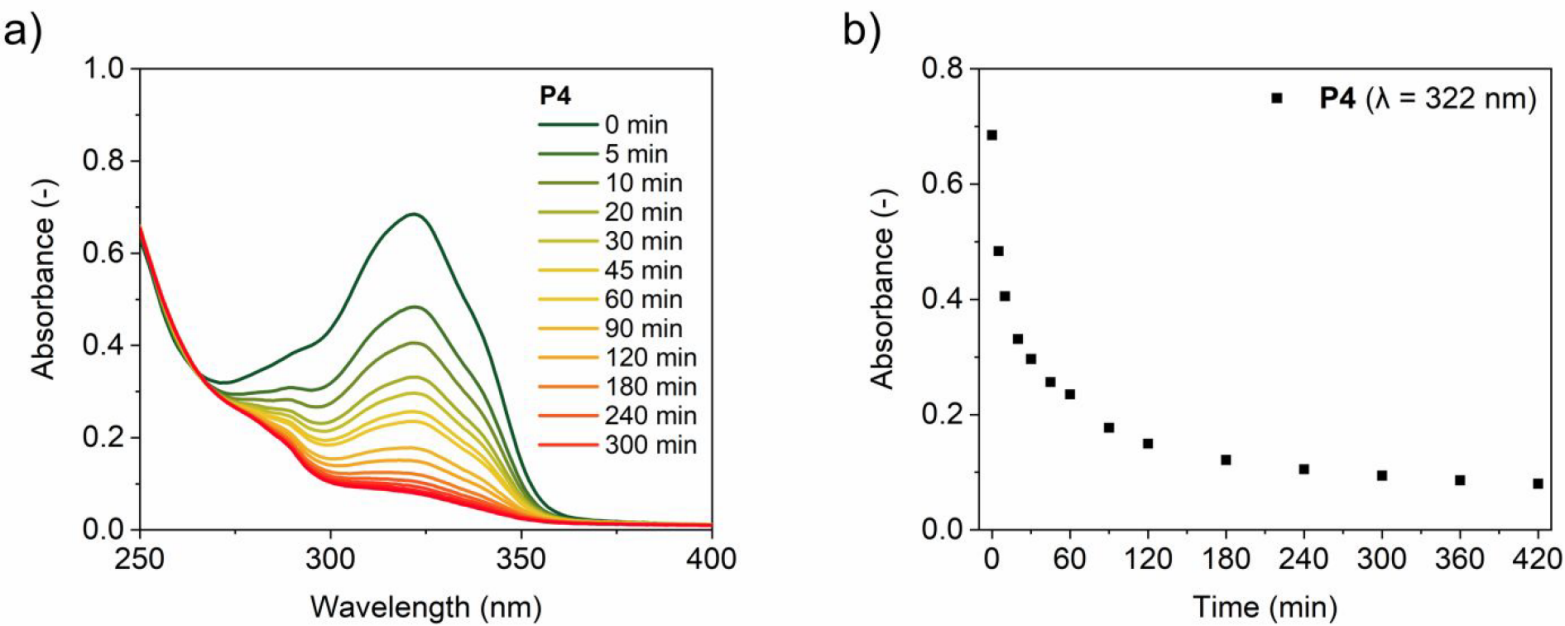
(a) UV–vis absorbance spectra of **P4** in water followed over time during coumarin dimerization (λ_irr_ = 365 nm), which was used to plot (b) the UV–vis absorbance maxima at λ = 322 nm against the cross-linking time. *c*_polymer_ = 1 mg mL^–1^.

Figure 2a shows the changes in the UV–vis spectra over time when dimerizing the coumarin moieties of **P4** in water. Plotting the absorbance at λ = 322 nm over time reveals that the decrease is exponential and significantly slows down after 5 h (Figure 2b). The coumarin conversion reached a conversion of 90% both in water and in THF after 6 h, as inferred from the molar extinction coefficients ε calculated from model compounds (see section 7 in the Supporting Information for details, Table S3). This conversion was corroborated by an 80–90% decrease of the monomer fluorescence centered around λ_em_ = 383 nm (using λ_ex_ = 320 nm) for both water and THF (Figure S54). The results show that the extent of the photodimerization is the same in both solvents. Importantly, filtration of the samples did not show significant changes in the intensity of the absorbance spectra (Figure S55), indicating the absence of large intermolecularly cross-linked aggregates after photodimerization in THF and water. Also, the coumarin dimerization was very robust and insensitive to the presence of oxygen: the reaction proceeded equally well with oxygen present in the solution as when the solution was degassed by argon bubbling (Figure S56). Additionally, reducing the light intensity by 50% did not influence the photodimerization process (Figure S57).

We then performed static and dynamic light scattering (SLS and DLS) measurements of **P4** in THF and water, before and after coumarin dimerization, to get more insights into the nature of the particles formed. In THF, **P4** shows a radius of gyration, *R*_G_, of 16.3 nm (Table 1 and Figure S58) and a hydrodynamic radius, *R*_H_, of 10.1 ± 0.2 nm (Table 1 and Figure S59). This gives a shape factor ρ = *R*_G_/*R*_H_ of 1.56, which indicates that **P4** may adopt a random coil conformation when dissolved in THF.^83^ In water, a small fraction of larger aggregates prevented the extraction of reliable values for *R*_G_ from the SLS measurements, evident by the mean aggregation number *N*_agg_ derived for **P4** in water by SLS (Table 1; see the Experimental Section in the Supporting Information for details). DLS, in contrast, showed an *R*_H_ of 4.3 ± 0.6 nm for **P4** in water, which is much smaller than the *R*_H_ of 10.1 nm in THF (Table 1 and Figure S59). Thus, hydrophobic interactions and intramolecular hydrogen-bonding interactions between BTA grafts cause **P4** to adopt a more compact conformation in water than in THF. On the basis of comparisons with previous systems, we conclude that **P4** forms SCPNs in water.^75,76^

**Table 1:** Hydrodynamic Radius (*R*_H_) and Radius of Gyration (*R*_G_) for **P4** before (BC) and after Cross-Linking (AC)^a^

polymer	solvent	*R*_H_ (nm)	no. of samples	*R*_G_ (nm)	*R*_avg_ (m^–1^)	*N*_agg_
**P4** BC	water	4.3 ± 0.6	6		0.026	6.7
**P4** AC	water	4.7 ± 0.8	5		0.029	8.6
**P4** BC	THF	10.1 ± 0.2	6	16.2	0.003	1.5
**P4** AC	THF	9.8 ± 0.5	5	13.6	0.003	1.8

a Coumarin dimerization was performed in water or THF with λ_irr_ = 365 nm for 6 h with *c*_polymer_ = 1 mg mL^–1^. The no. of samples is the number of separately prepared samples. *R*_avg_ is the averaged Rayleigh ratio obtained from 30° to 150°, and *N*_agg_ is the aggregation number of polymer chains per particle calculated from SLS.

After coumarin dimerization, the particle sizes remain almost the same. In THF, *R*_G_ is slightly smaller at 13.6 nm (Table 1 and Figure S58), but *R*_H_ is almost identical at 9.8 ± 0.5 (Table 1 and Figure S59). Also in water, the *R*_H_ of 4.7 ± 0.8 remains almost the same. The good agreement between the average Rayleigh ratio *R*_avg_ and the aggregation number *N*_agg_ in THF before and after cross-linking (Table 1) further corroborates that cross-linking occurs predominantly intramolecularly in both solvents. All in all, cross-linking of the coumarin grafts in **P4** in either water or THF does not significantly alter the size of the particles in solution. Whereas random coil-like conformations are formed in THF, compact SCPNs are obtained in water, before and after coumarin dimerization.

### Effect of Coumarin Dimerization on Particle Size

**P4** adopts a random coil-like conformation in THF before and after cross-linking and compact SCPNs in water. We were interested in how the cross-linked states obtained in both solvents affect the sizes of the particles when taken into water or buffered media. We first investigate the two folding pathways, PW1 and PW2, outlined in Scheme 1, in more detail using DLS in water and THF. Subsequently, we apply a combination of size exclusion chromatography (SEC) and DLS in the biologically relevant medium phosphate buffered saline (PBS). For a complete overview of the evolution of the *R*_H_ of **P4** measured in different relevant solvents, see Table S4.

**Scheme 3:**
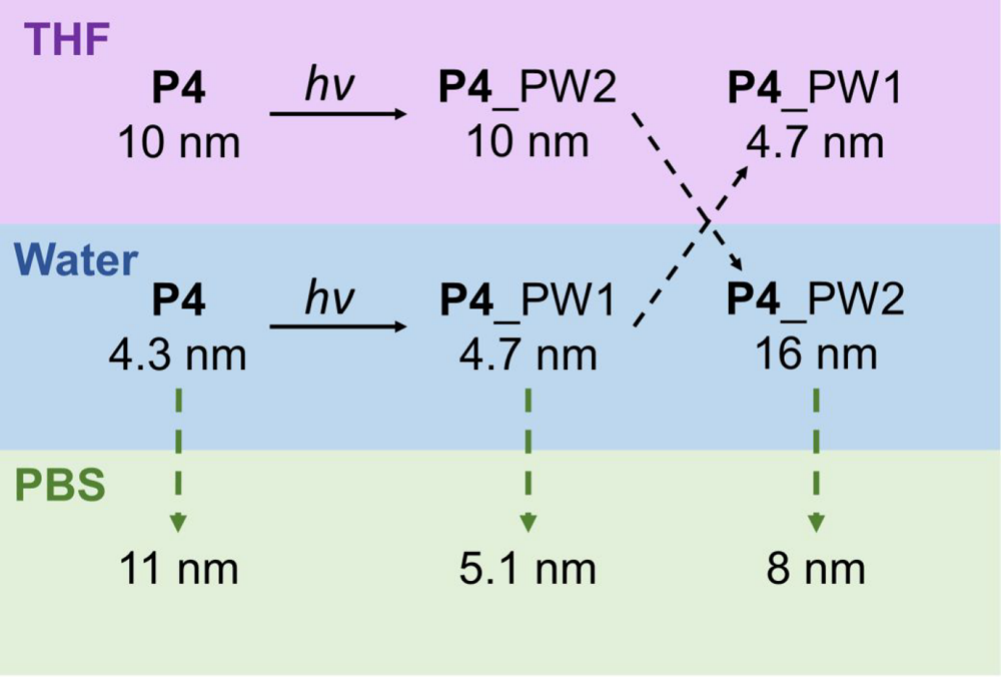
Overview of the Hydrodynamic Radii of **P4** as Determined by DLS before Cross-Linking, and after Cross-Linking via Pathway 1 (PW1) or 2 (PW2), Measured in the Corresponding Solvents THF (Top Row) or Water (Middle Row); Hydrodynamic Radii after Switching the Solvent to PBS (Bottom Row)

Cross-linking **P4** in water (SCPN) or THF (random coil) does not greatly affect the particle size, but particles formed in water are significantly smaller (∼5 nm) than those formed in THF (∼10 nm) (Table 1 and Scheme 3). Interestingly, when THF is removed and cross-linked **P4** (**P4**_PW2) is redissolved in water, *R*_H_ increases to 16 nm. In contrast, when **P4** cross-linked in water via PW1 (**P4**_PW1) is redissolved in THF, *R*_H_ stays at 4.7 nm (Scheme 3). The strong increase in size for **P4**_PW2 in water indicates the formation of multichain aggregates, whereas the compact conformation of **P4**_PW1 in water is retained in THF, a solvent in which hydrogen bonds are broken. Likely, cross-linking in THF results in unideal cross-links, which prevent the collapse/folding of the polymer chain in water due to mobility constraints put on the polymer backbone. As a result, the polymer can no longer sufficiently shield all hydrophobic grafts from the water phase, resulting in aggregation due to interactions between multiple polymer chains. In contrast, cross-linking a well-defined folded/collapsed state locks in the compact polymer conformation so that even in the absence of hydrogen bonding small particles are retained.

The particles prepared via both pathways were further investigated in PBS. Nile Red fluorescence spectra measured for **P4** in PBS (Figure S71a) show a clear blue-shift of the Nile Red emission compared to free Nile Red in PBS. This indicates that **P4** forms hydrophobic compartments, also in PBS (Figure S72). Remarkably, DLS measurements in PBS show significant differences in the particle size compared to water. **P4** before cross-linking forms larger particles (*R*_H_ = 11 nm) in PBS compared to water (*R*_H_ = 4.3 nm). **P4**_PW1, in contrast, remains small in PBS (*R*_H_ = 5.1 nm). Interestingly, **P4**_PW2 shows an *R*_H_ of 8 nm in PBS, which is significantly smaller than the *R*_H_ of 16 nm in water. Moreover, the *R*_H_ of **P4**_PW1 and **P4**_PW2 did not significantly change between 20 and 60 °C, indicating good size stability (Table S5). Thus, also in PBS cross-linked **P4**_PW1 forms compact conformations.

**Figure 3:**
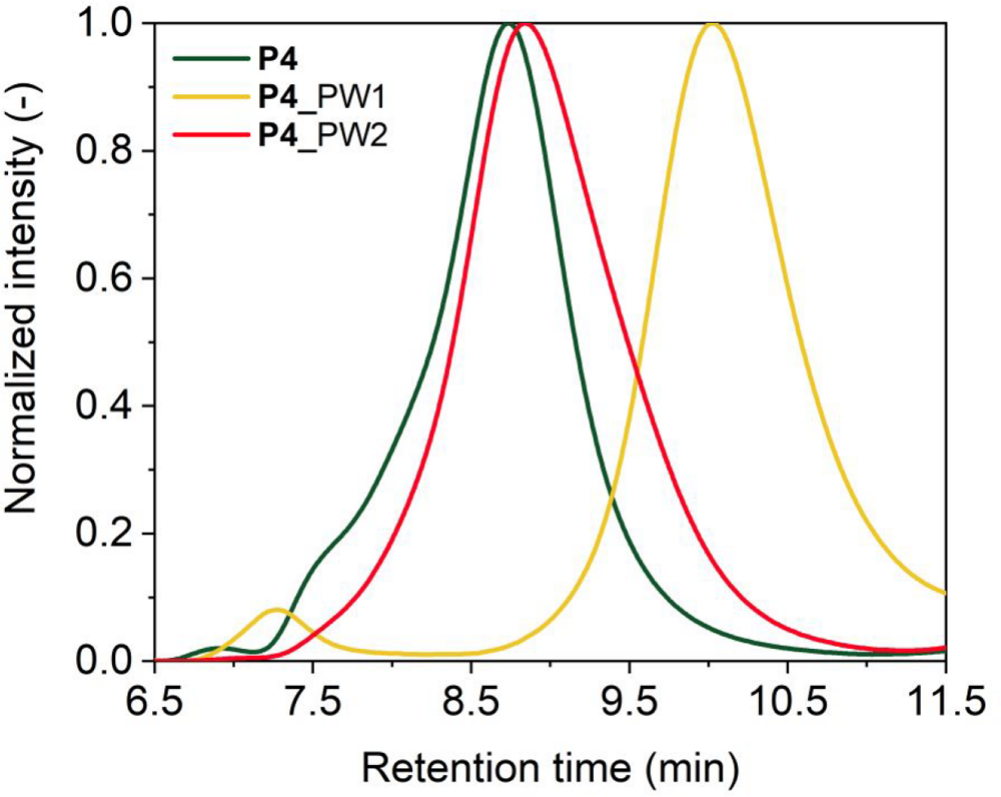
SEC traces in PBS of **P4** before cross-linking and after folding via pathway 1 (**P4**_PW1) or pathway 2 ((**P4**_PW2) with *c*_polymer_ = 1 mg mL^–1^. A higher retention time indicates a smaller apparent molecular weight.

The relative hydrodynamic radii of **P4** prepared via both pathways were further investigated by SEC in PBS. The normalized SEC traces (Figure 3) are monomodal, albeit a small shoulder is present for **P4** at low retention times. For **P4** before cross-linking, a retention time around 8.5 min is observed, corresponding to an apparent molecular weight of *M*_n,app_ = 46 kDa. This *M*_n,app_ is much larger than that observed for **P4**_PW1, with a shift to longer retention times corresponding to *M*_n,app_ = 9.5 kDa. Finally, **P4**_PW2 shows an *M*_n,app_ of 32 kDa, in between the other two measurements. The results obtained by SEC and DLS in PBS are consistent: coumarin dimerization in **P4** in a folded state in water stabilizes the particle and prevents aggregation in PBS, whereas in the absence of cross-linking, as well as for cross-linking in a random coil-like conformation in THF, larger particle sizes are observed. More details are given in the Supporting Information (Figures S60–S61 and Table S6).

### Effect of Coumarin Cross-Linking on Particle Stability

**Figure 4:**
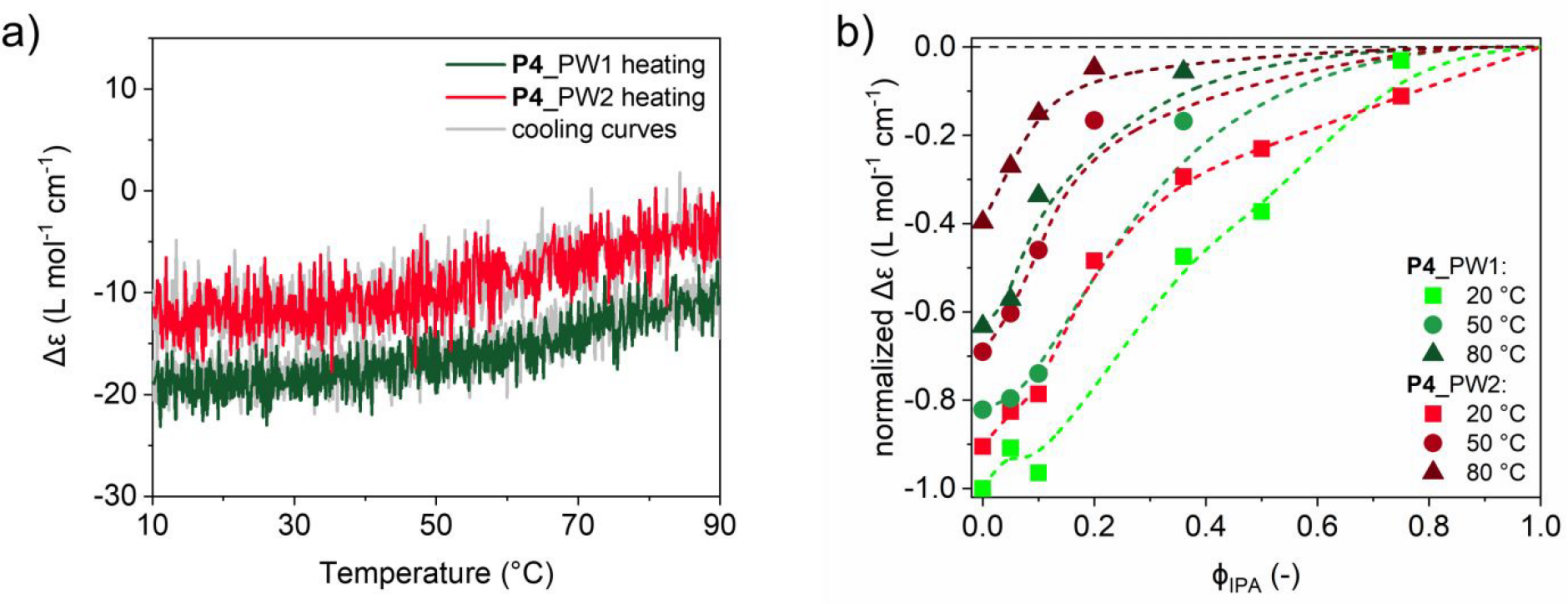
(a) CD heating and cooling curves of **P4** after folding via PW1 or PW2 recorded at λ = 225 nm in water. (b) Normalized Δε of **P4**_PW1 and **P4**_PW2 obtained from the CD cooling curves recorded at λ = 225 nm in water/IPA mixtures as a function of ϕ_IPA_ at different temperatures. The dashed lines are added to guide the eye. The Δε was normalized between −1 and 0, with −1 corresponding to the largest magnitude of the CD effect observed, which occurs at 10 °C and ϕ_IPA_ = 0. *c*_polymer_ = 1 mg mL^–1^ and *c*_BTA_ = 41 μmol.

Covalent cross-linking after folding into an SCPN allows the formation of particles that remain small in PBS and in a good solvent such as THF. We anticipate that the stability of the hydrophobic interior in the compact particles is higher than those in the less compact particles. This difference can be probed by evaluating the degree of BTA aggregation for the different folding pathways. Therefore, temperature-dependent heating and cooling curves of **P4**_PW1 and **P4**_PW2 were recorded at λ = 225 nm (Figure 4a). The absolute molar circular dichroism |Δε| at 10 °C is lower for **P4**_PW2 (Δε = −13 L mol^–1^ cm^–1^) than for **P4**_PW1 (Δε = −18 L mol^–1^ cm^–1^). Upon increasing the temperature to 90 °C, the BTAs partially disassemble, resulting in a decrease of 70% and 40% for **P4**_PW2 (Δε = −4 L mol^–1^ cm^–1^) and **P4**_PW1 (Δε = −11 L mol^–1^ cm^–1^), respectively. The BTA self-assembly process is fully reversible; in both cases the CD signal is regained upon cooling. The CD spectra of the different **P4** systems and temperature-dependent CD spectra of **P4** before cross-linking are included in the Supporting Information for comparison (Figures S62 and S63).

The stability of the hydrophobic interior was additionally studied in mixtures of water and isopropanol (IPA), a solvent that competes with hydrogen bonds between the BTA grafts, as evidenced by the absence of a CD effect in pure isopropanol (Figure S64).^75^ We simultaneously investigated the effect of solvent composition and temperature to understand the relative strength of the BTA self-assembly in **P4**_PW1 and **P4**_PW2. Figure 4b shows the normalized Δε values of both cross-linking pathways plotted as a function of IPA fraction (ϕ_IPA_) for 20, 50, and 80 °C (see the Supporting Information for details, Figures S64–S67). The normalized Δε of **P4**_PW1 and **P4**_PW2 decreases with increasing temperature and ϕ_IPA_. For all data points, the extent of BTA disassembly for **P4**_PW2 is higher than for **P4**_PW1. In fact, at any given solvent composition, **P4**_PW2 has a 30 °C penalty in temperature stability compared to **P4**_PW1. Likewise, at any given temperature, less IPA is needed to fully disrupt the BTA self-assembly for **P4**_PW2 than for **P4**_PW1. The results corroborate that PW1 results in SCPNs with a better structured and more stable hydrophobic interior because of the larger extent of BTA self-assembly compared to PW2, which forms less well-structured hydrophobic interiors.

### Reversibility of Coumarin Dimerization in Cross-Linked Nanoparticles

**Figure 5:**
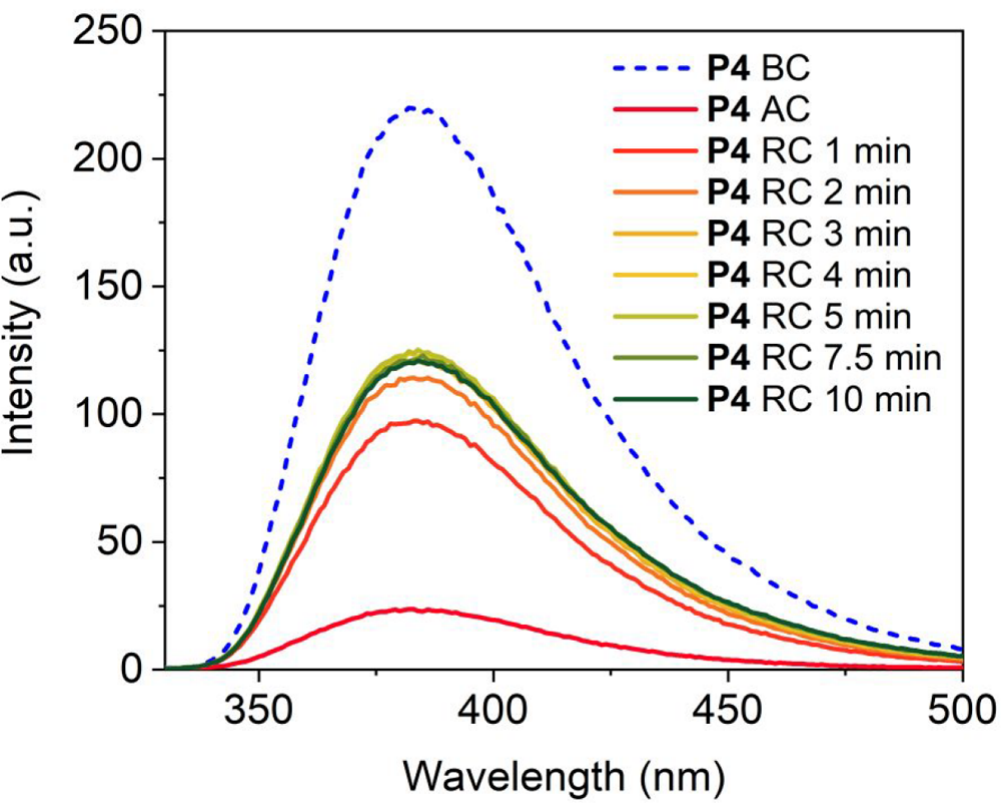
Fluorescence spectra of **P4** before (BC) and after cross-linking at λ_irr_ = 365 nm (AC) via PW1. The ring-opening cycloreversion (RC) of the coumarin dimers was then followed for 10 min during illumination with UV-light at λ = 254 nm (RC). *c*_polymer_ = 1 mg mL^–1^.

Coumarin dimers can revert back, at least partially, to their initial state when light of 254 nm is used, even when the coumarin dimers are embedded in polymeric systems.^84,85^ To assess the reversible nature of **P4**_PW1, ring-opening cycloreversion (RC) of the coumarin dimers was induced. Figure 5 shows the coumarin monomer fluorescence before (BC) and after cross-linking (AC) via PW1 as well as the increase in the fluorescence over time due to the RC. After 10 min, the fluorescence intensity started to decrease once more, indicative of polymer degradation or bleaching of the coumarin because of the harsh UV-light. From the corresponding absorbance spectra (Figure S69), we quantified that 15% of the coumarin monomeric species was regained, shown by the increase from 10% of the monomeric species left AC to 25% after the RC. As a comparison, the RC at λ = 254 nm of the model dimer **4** in acetonitrile led to the full recovery of the monomeric species within 10 min (Figure S68), without degradation. The rate difference between the reverse reaction of **P4**_PW1 compared to the model dimer **4** is interesting. Dimer **4** was present in a dilute solution, making it unlikely that the monomer can recombine after the RC occurred. This in contrast to **P4**, where the coumarin moieties are packed closely together in the hydrophobic pocket. As such, upon the RC, the two re-formed monomers remain in close proximity, greatly increasing the chance of recombination upon photoexcitation. Hence, the much higher local concentration of coumarin grafts in **P4** compared to the small molecule analogue likely shifts the reaction equilibrium toward the dimer state. SEC in PBS was performed to check for potential degradation of **P4**_PW1 following the RC (Figure S70). No significant change in the retention time was observed, indicating that **P4** did not degrade significantly within the 10 min experimental time frame.

### Encapsulation and Release of Model Compounds in Folded SCPNs

**Figure 6:**
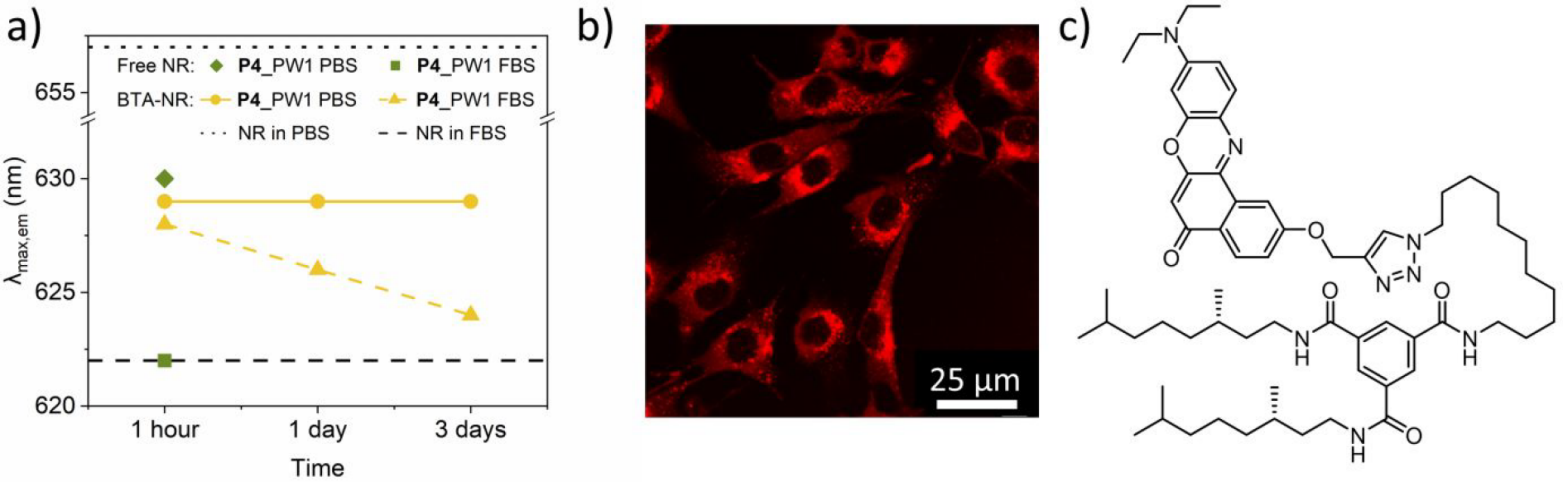
(a) Nile Red fluorescence of **P4**_PW1 samples in PBS and FBS-PBS plotted as the fluorescence maxima λ_max_ of Nile Red and BTA-NR against time. For NR measurements: *c*_polymer_ = 0.2 mg mL^–1^ and *c*_NR_ = 2 μM. For BTA-NR measurements: *c*_polymer_ = 1 mg mL^–1^ and *c*_BTA-NR_ = 5.55 μM. (b) Confocal microscopy image of **P4**_PW1 and BTA-NR incubated with HeLa cells. (c) Chemical structure of BTA-NR (**6**).

As **P4**_PW1 forms stable, structured, and compact SCPNs, we anticipate that they can retain their folded properties in complex media and hereby reduce undesired interactions of biological molecules with cargo embedded in the SCPN’s interior. To evaluate this, we focus on the encapsulation capacity of **P4**_PW1 using the dye Nile Red. To trap the dye firmly inside the hydrophobic interior, Nile Red was covalently attached to a BTA (BTA-NR (**6**), Figure 6c). Following our recent results,^86^ BTA-NR mixes into BTA aggregates of SCPNs via molecular recognition. The fluorescence maximum (λ_max,em_) of the dye can be used as a measure of the encapsulation capability and hereby the stability of **P4**_PW1 in increasingly complex media. If the SCPNs unfold, the polarity around the dye changes and the λ_max,em_ will change.

Figure 6a summarizes the observed λ_max,em_ of BTA-NR mixed with **P4**_PW1 in PBS buffer and PBS complemented with 20% FBS. The results are compared to λ_max,em_ of NR in pure water (dotted line) and FBS (dashed line). As a reference, the less compact SCPNs were evaluated (**P4** and **P4**_PW2) as well, and free Nile Red was mixed into the SCPNs (see the Supporting Information for more details; Figures S71–S76). After 1 h, BTA-NR mixed into **P4**_PW1 shows a λ_max,em_ around 627 nm in both PBS and FBS-PBS. These similar values indicate that the hydrophobic proteins in FBS do not interact with the interior of the SCPN, nor do they extract BTA-NR from the particle. This is in sharp contrast to free NR mixed into **P4**_PW1 where λ_max,em_ is 630 and 622 nm in PBS and FBS-PBS, respectively. These values indicate the rapid extraction of NR into the hydrophobic proteins present in FBS. In PBS, the λ_max,em_ of BTA-NR mixed into **P4**_PW1does not change over a duration of 3 days. This is expected because there are no competitive interactions present in the medium. In FBS, a slow decrease of λ_max,em_ is observed over 3 days, indicating slow exchange dynamics of the BTA-NR present inside **P4**_PW1 toward the FBS proteins. The other two polymer systems **P4** and **P4**_PW2 show similar behavior (Figure S75). Although the folding pathway influences the size and compactness of the nanoparticles, it does not result in a loss of dynamicity in either of the cross-linked systems. The observed slow release dynamics the BTA-NR might prove useful in designing drug release profiles for potential applications where stable, compact nanoparticles are desired. In addition, the results show that the interaction between the SCPN interior and FBS constituents is a slow process, which is promising in view of catalytic applications.

Finally, the stability of **P4**_PW1 was investigated by incubating **P4**_PW1 premixed with BTA-NR for 24 h in the presence of HeLa cells and recording the fluorescence spectra of NR by using confocal microscopy. Figure 6b shows that the SCPNs are taken up by the HeLa cells. The elongated shape of the cells is a promising indicator for biocompatibility, in line with the Jeffamine-based SCPNs we previously studied.^86^ The fluorescence spectra extracted from the confocal microscopy images show a blue-shift of the λ_max,em_ BTA-NR in **P4**_PW1 (Figure S77), indicative of a lowering of the polarity around the BTA-NR dye. The highly competitive environment of the living cells affects **P4**_PW1 in a similar fashion as we observed before for similar systems where Nile Red was covalently attached to the polymer.^86^ The results show that although the stability of the SCPNs improves as a result of the covalent cross-links, the dynamic nature of the system is retained, also intracellularly.

## Conclusions

In this work, two different self-assembly pathways in the preparation of SCPNs were systematically studied by using a combination of CD, UV–vis, and fluorescence spectroscopy as well as DLS and PBS-SEC in aqueous media. Amphiphilic acrylamide-based copolymers were prepared via a postfunctionalization approach, yielding polymers comprising different fractions of hydrophilic Jeffamine@1000, hydrophobic BTA, and dodecyl chains as well as cross-linkable coumarin grafts. The combination of 5% BTA and 5% coumarin found in **P4** gave the best combination of cross-linking potential and BTA self-assembly. In pathway 1, the polymer was covalently cross-linked after supramolecular self-assembly via hydrogen bonding and hydrophobic collapse. In pathway 2, the polymer was first covalently cross-linked in a random coil state, after which the supramolecular self-assembly was induced. Upon cross-linking, the polymers formed SCPNs in water with a small *R*_H_ of 5 nm and a high BTA self-assembly in the case of PW1 and a larger size of *R*_H_ = 16 nm and a lower BTA self-assembly in the case of PW2. Only SCPNs formed via PW1 remained small when taken into PBS, as determined by DLS and SEC. These SCPNs additionally contained hydrophobic interiors with higher temperature and solvent stability compared to those prepared by PW2, as probed by temperature-dependent CD measurements using the competitive hydrogen-bonding solvent IPA. Our results are reminiscent of the folding of ribonuclease S in nature, in which only the correct folding following first hydrogen-bonding and hydrophobic interactions, and then the formation of disulfide bridges, results in the active species.

The fluorescence emission maximum of Nile Red was then used to probe interactions with increasingly complex media. Introducing supramolecular recognition as stabilizing factor via BTA-NR resulted in excellent short-term stability in 20% FBS in PBS, while simultaneously showing that SCPNs formed via PW1 retain good dynamicity, corroborated by the slow BTA-NR exchange dynamics over a 3 day period. Lastly, the current system shows good biocompatibility as the polymer is nontoxic and taken up by HeLa cells.

This research highlights the importance of preorganization during covalent cross-linking and the effect it has on particle structure and dynamic properties. Additional covalent cross-linking following folding through hydrogen-bond formation provides SCPNs with advantageous properties, but only when the polymer is correctly folded prior to cross-linking. In the absence of such preorganization, covalent cross-linking will freeze in a suboptimal conformation, preventing optimal folding of the SCPNs, leading to a decrease in the desired properties. Therefore, the folding pathway of SCPNs is crucial in controlling and determining the final system properties and should be taken into account when designing new SCPN systems toward biological applications that require high levels of control over size and stability, while retaining dynamic properties.

## Supporting Information Available

Detailed synthetic procedures and characterization, ^1^H, ^13^C, and ^19^F NMR spectroscopy, infrared spectroscopy, gel permeation chromatography, UV–vis absorption spectroscopy, fluorescence emission spectroscopy, circular dichroism spectroscopy, and static and dynamic light scattering (PDF)

## Abbreviations

AC: after cross-linking
BC: before cross-linking
RC: ring-opening cycloreversion
SCPN: single-chain polymeric nanoparticle
BTA: benzene-1,3,5-tricarboxamide
PFPA: pentafluorophenyl acrylate
pPFPA: poly(pentafluorophenol acrylate)
DMEM: Dulbecco’s modified eagle’s serum
FBS: fetal bovine serum
PBS: phosphate buffered saline
NR: Nile Red.
