# Supplementary material for: En Route to Stabilized Compact Conformations of Single-Chain Polymeric Nanoparticles in Complex Media
Source: Macromolecules. 2022 Jul 13;55(14):6220–30. doi: 10.1021/acs.macromol.2c00930 (PMC9330768; doi:10.1021/acs.macromol.2c00930)
Supplement: Supplementary file 1 — ma2c00930_si_001.pdf [file ma2c00930_si_001.pdf]

Supporting Information for:

***En route* to stabilized compact conformations of single chain polymeric nanoparticles in complex media.**

Stefan Wijker[1], Linlin Deng[1], Fabian Eisenreich[1], Ilja K. Voets[2], Anja R. A. Palmans*[1]

[1]Institute for Complex Molecular Systems, Laboratory of Macromolecular and Organic Chemistry, Eindhoven University of Technology, P.O. Box 513, 5600 MB, Eindhoven, The Netherlands

[2]Laboratory of Self-Organizing Soft Matter, Department of Chemical Engineering and Chemistry, Institute for Complex Molecular Systems, P.O. Box 513, 5600 MB, Eindhoven, The Netherlands

Contents

1. Experimental section 3
2. Small molecule synthesis and characterization 7
Synthesis of 7-ethoxy-4-methyl-coumarin (3): 7
Synthesis of 7-ethoxy-4-methyl-coumarin dimer (4): 10
Characterization of (S,S)-BTA-C_{11}-amine (5): 12
(S,S)-BTA-NR (6): 13
Characterization of 7-(2-aminoethoxy)-4-methyl-coumarin (TFA-salt) (7): 13
3. Polymer synthesis and characterization 15
Synthesis of pentafluorophenyl acrylate (8): 15
Synthesis of poly(pentafluorophenyl acrylate) (9) (pPFPA): 17
End-group modification of poly(pentafluorophenyl acrylate) (10): 20
Amine post-functionalization of poly(pentafluorophenyl acrylate) (P1-P7): 22
UV-vis spectrum of P1 – P6: 35
^{1}H NMR of P5 in D_2O: 35
Nile Red fluorescence in P1 – P7: 36
CD heating and cooling curves of P3 – P6: 37
4. Sample preparation of polymer solutions 39
5. Sample filtration 39
6. Cross-linking setup 40
7. Coumarin cross-linking and spectroscopic properties 40
8. P4 characterization 43
CD spectra of P4 in water and THF 43
Light scattering data of P4 before and after cross-linking 46
UV-vis absorbance spectra of P4 before and after cross-linking 43
DLS characterization of P4 before and after cross-linking ..... **Error! Bookmark not defined.**

SEC characterization of P4 and P6 before and after cross-linking 48
CD characterization of P4 before and after cross-linking...50
CD characterization of P4 before and after cross-linking in water / IPA 51
9. Reverse cross-linking experiments of coumarin..53
10. Nile Red emission spectra of P4 before and after cross-linking...54
11. Nile Red emission spectra of P4 in HeLa cells...57
12. References...58

1. Experimental section

Materials. All commercial reagents were purchased from Acros Organics, Fischer Scientific, Merck, TCI, or Sigma-Aldrich, except for Jeffamine® M-1000 (Jeffamine, M_w = 1000, propylene oxide/ethylene oxide ratio of 3/19), which was purchased from Huntsman Holland BV. Solvents were purchased from Biosolve, except for anhydrous 1,4-dioxane, which was purchased from Sigma-Aldrich, and trichloroethylene, which was purchased from Merck. Dry diethylether and THF were obtained using an MBRAUN SPS-800 solvent purification system. PBS tablets were purchased from Sigma-Aldrich, DMEM (Gibco's DMEM, high glucose, HEPES, no phenol red) and FBS (Gibco's FBS, qualified) were purchased from Thermo Fischer. Deuterated solvents were purchased from Cambridge Isotope Laboratories. AIBN was recrystallized from methanol. All other materials were used as received. N^1-(11-aminoundecyl)-N^3,N^5-bis((S)-3,7-dimethyloctyl)benzene-1,3,5-tricarboxamide ((S,S)-BTA-C_{11}-amine, Figures S6 - S7) (**5**)[1], N1-(11-(4-(((9-(diethylamino)-5-oxo-5H-benzo[a]phenoxazin-2-yl)oxy)methyl)-1H-1,2,3-triazol-1-yl)undecyl)-N3,N5-bis((S)-3,7-dimethyloctyl)benzene-1,3,5-tricarboxamide (BTA-NR) (**6**)[2], and 7-(2-aminoethoxy)-4-methyl-coumarin (coumarin amine, Figures S8 - S9) (**7**)[3] were synthesized previously according to literature procedures.

Nuclear Magnetic Resonance (NMR) Spectroscopy. ^{1}H NMR, ^{13}C NMR, and ^{19}F NMR spectra were recorded on a Bruker Varian 400MR 400 MHz or a Bruker Varian Mercury Vx 400 MHz (400 MHz for ^{1}H NMR, 100 MHz for ^{13}C NMR, and 375 MHz for ^{19}F NMR). ^{19}F NMR used a recycle delay of 10 seconds. ^{1}H NMR and ^{13}C NMR chemical shifts are reported in ppm downfield from tetramethylsilane (TMS) as internal reference. For ^{19}F NMR, a recycle delay of 10 seconds was used. The deuterated solvent used is indicated for each spectrum. For the abbreviations used: s = singlet, d = doublet, dd = double doublet, t = triplet, q = quartet, m = multiplet.

Infrared (IR) Spectroscopy. Fourier transform infrared (FT-IR) spectra were recorded in ATR mode on a PerkinElmer FT-IR Spectrum Two equipped with a PerkinElmer UATR Two (4000 – 450 cm^{-1}, 16 scans).

Matrix Assisted Laser Desorption/Ionization Time of Flight Mass Spectra (MALDI-TOF-MS). MALDI-TOF-MS spectra were recorded on a Bruker Autoflex Speed MALDI-TOF instrument equipped with a 355 nm Nd:YAG smartbeam laser. The laser had a maximum repetition rate of 1 kHz. α-cyano-4-hydroxycinnamic acid (CHCA) and trans-2-[3-(4-tert-butylphenyl)-2-methyl-2-propenylidene]malononitrile (DCTB) were used as sample matrices.

Size Exclusion Chromatography (SEC): SEC measurements in DMF were carried out on a Shimadzu prominence-I LC-2030C 3D system operated at 50 °C equipped with a Shimadzu RID-10A RI detector and a LC-2030/2040 PDA detector on a Shodex SEC-KD-804 column (exclusion limit = 400 kDa, i.d. = 0.8 cm, L = 300 mm), with DMF as eluent containing 10 mM LiBr at a constant flow rate of 1 mL min^{-1}. The column was calibrated against poly(ethylene oxide) (PEO) (Polymer Laboratories). SEC measurements in THF were carried out on a Shimadzu prominence-I LC-2030C 3D system operated at 40 °C equipped with a Shimadzu RID-10A RI detector and a LC-2030/2040 PDA detector on a PLgel 5 mm mixed-C (200 – 2000 kDa) and PLgel 5 mm mixed-D (200 – 40 kDa) column combined in series (exclusion limit = 2000 kDa, i.d. = 0.75 cm, L = 300 mm), with THF as eluent at a constant flow rate of 1 mL min^{-1}. The column was calibrated against poly(styrene) (Polymer Laboratories). SEC measurements in 1x PBS (pH = 7.4) were carried out on a Shimadzu CBM-20A System at 20 °C equipped with a Shimadzu RID-10A RI detector, SIL-20A autosampler, and 2 LC-20AD pumps on a Shodex OHpak SB-804 HQ column (exclusion limit = 1000 kDa, i.d. = 0.8 cm, L = 300 mm) with a TSKgel SW_{XL} type guard column (i.d. = 0.6 cm, L = 40 mm), with PBS as an eluent at a constant flow rate of 0.8 mL min^{-1}. The column was calibrated against poly(ethylene oxide) (PEO) (Polymer Laboratories).

UV-vis Spectroscopy. UV-vis spectra were recorded on a Jasco V-650 spectrophotometer or a Jasco V-750 spectrophotometer (200 nm min^{-1} scanning speed, 3 accumulations). Quartz cuvettes with a pathlength of 1 cm were used. Standard baseline corrections were performed.

Fluorescence Spectroscopy. Fluorescence spectra were recorded on a Varian Cary Eclipse fluorescence spectrophotometer. Excitation wavelength was either 320 nm for coumarin measurements (medium scanning speed, 5 nm slit width) or 560 nm for Nile Red measurements (slow scanning speed, 5 nm slit width). Quartz cuvettes with a pathlength of 1 cm x 1 cm were used, except for BTA-NR measurements, which used 1 cm x 0.2 cm pathlength cuvettes instead. The sensitivity was chosen appropriately per experiment. For Nile Red measurements, in order to more accurately determine the emission maxima, Lowess smoothing was performed and the emission maxima was chosen as the wavelength corresponding to the maximum emission intensity after smoothing.

Circular Dichroism (CD) Spectroscopy. Circular Dichroism spectra were recorded on a Jasco J-815 CD spectrophotometer equipped with a PFD-425S/15 Peltier-type temperature controller (scanning speed was 0.25 nm sec^{-1}, 3 accumulations). Standard baseline corrections were performed. Quartz cuvettes with a pathlength of 5 mm were used. For temperature-dependent measurements, the CD signal was monitored at λ = 225 nm, with a heating and

cooling rate of 1 °C min^{-1}, and a 5 minute equilibration time between heating and cooling. The molar circular dichroism $\Delta\varepsilon$ was calculated according to $\Delta\varepsilon = (CD)/(32982cl)$, with CD the recorded Cotton effect in mdeg, c the BTA concentration in mol L^{-1} (c_{BTA} = 41 μM in all cases), and l the pathlength in cm.

Static Light Scattering (SLS). Static Light Scattering measurements were recorded on an ALV CGS-3 instrument equipped with an ALV-7004 digital correlator and a λ = 532 nm laser in glass cuvettes (i.d. = 0.8 cm) after sample filtration to remove dust. The scattering intensity was recorded as a function of the scattering vector $q = 4\pi sin(\theta/2)/\lambda$, with θ the scattering angle in degrees and λ the wavelength of the laser in nm. The scattering intensity was recorded at scattering angles of 30° to 150° with a step size of 10°, at 6 measurements of 15 seconds per angle. The average scattering intensity was used to calculate the radius of gyration R_G via the Guinier approximation in a plot of lnR_θ against q^2 according to $ln\Delta R_\theta = 1 - \frac{R_G^2 \cdot q^2}{3}$, where only the measurements at scattering angles such that the condition $qR_G < 1{,}3$ was met, were used. R_θ is the Rayleigh ratio of the sample, calculated as: $R_\theta = \frac{I_{sample} - I_{solvent}}{I_{toluene}} \cdot (\frac{n_{solvent}}{n_{toluene}})^2 \cdot R_{\theta,\,toluene}$, $q = \frac{4\pi n_{solvent}}{\lambda} \cdot \sin\frac{\theta}{2}$, and I the average scattering intensity at each angle for the sample, solvent, and toluene respectively. n is the refractive index of the solvent and of toluene (at λ = 532 nm and T = 20 °C, n_{THF} = 1.376[4] and $n_{toluene}$ = 1.5019[5]; n_{water} = 1.335, 1.333, and 1.329 at 20, 40, and 60 °C respectively.). $R_{\theta,\,toluene}$ is the Rayleigh ratio of toluene (0.0021 m^{-1}). R_G follows from the slope in the Guinier plot: $R_G = \sqrt{3 * slope}$. The molecular weight of the ensemble of scattering particles, regardless of their association state, was approximated using the partial Zimm equation $\frac{Kc}{R_\theta} = \frac{1}{M_w}(1 + \frac{q^2 R_g^2}{3})$, by extrapolation of the SLS data to q = 0 for low concentration samples (1 mg mL^{-1}), for which we assume P(0) = 1 and S(q) = 1. From this it follows that $\lim_{q \to 0} \frac{Kc}{R_\theta} = \frac{1}{M_w}$, with $K = \frac{4\pi n_0^2 (\frac{dn}{dc})^2}{N_A \lambda^4}$. c is the scatterer concentration, R_θ is the Rayleigh ratio of the sample, n_o is the refractive index of the solvent, $\frac{dn}{dc}$ is the specific refractive index increment, N_A is Avogadro's number, and λ is the wavelength of the laser in nm. The specific refractive index increments of our polymers in water and THF were estimated from literature for poly(ethylene oxide) homopolymers as $\frac{dn}{dc}$ = 0.135 mL g^{-1} in water[6] and $\frac{dn}{dc}$ = 0.067 mL g^{-1} in THF.[7] The amount of polymers per particle was calculated as $N_{agg} = \frac{M_w}{M_{w,polymer}}$ with $M_{w,polymer}$ the theoretical molecular weight of one polymer (180 000 g mol^{-1}).

Dynamic Light Scattering (DLS). Dynamic Light Scattering measurements were recorded in tandem with the SLS data on an ALV CGS-3 instrument equipped with an ALV-7004 digital correlator and a λ = 532 nm laser in glass cuvettes (i.d. = 0.8 cm) after sample filtration to remove dust. The scattering intensity was recorded as a function of the scattering vector $q = 4\pi sin(\theta/2)/\lambda$, with θ the scattering angle in degrees and λ the wavelength of the laser in nm. The scattering intensity was recorded at scattering angles of 30° to 150° with a step size of 10°, at 6 measurements of 15 seconds per angle. First the second order autocorrelation curve was calculated from the scattering intensity of the sample I_{sample} according to $g^2(\tau) = \frac{<I(t)\cdot I(t+\tau)>}{<I(t)>^2}$, at time t with delay times τ, from which the first order autocorrelation function is calculated using the Siegert equation according to $g^1(\tau) = \sqrt{\frac{g^2(\tau)-1}{\beta}}$, with β a machine specific correction factor. The obtained correlation function depends on the decay rate Γ of the particles in solution. For polydisperse samples, $g^1(\tau) = \int G(\Gamma)e^{-\Gamma\tau}d\Gamma$. The fluctuations in the scattering intensity were analyzed by applying the CONTIN algorithm to the first order autocorrelation function derived from the trace of the scattering intensity using the After-ALV software by Dullware Inc. to obtain the decay rate Γ of the particles in solution. The CONTIN algorithm uses an inverse Laplace transform to find a distribution of Γ best describing $g^1(\tau)$. The diffusion coefficient of the particles D is derived from the slope in a plot of Γ as a function of q^2, which is used to calculate the hydrodynamic radius R_H via the Stokes-Einstein equation as $R_H = k_BT/(6\pi\eta D)$, with k_B the Boltzmann constant, T the temperature of the solution in K, and η the solvent viscosity (η_{water}[8] = 1.0016 mPa s (293 K), 0.6527 (313K), 0.4660 (333 K), η_{THF}[9] = 0.49 (293 K), η_{DMF}[10] = 0.92 (293 K)). A typical value of R^2 for the linear fit is 0.9.

Dialysis. Dialysis was performed using Spectra/Por® Standard RC Dry Dialysis membranes (M_W cutoff 6 – 8 kDa) after soaking them in demineralized water for 30 minutes.

PBS. 1x PBS (pH = 7.4) was prepared using PBS tablets. The pH was adjusted using 6M HCl or 6M NaOH solutions.

NR sample preparation. Nile Red (NR) was added to 1 mg mL^{-1} polymer solutions by injection from an ethanol stock solution (1 mM) to a NR concentration of 10 μM, upon which the samples were diluted 5 times with either water, PBS, DMEM, or 10 vol% FBS in DMEM (FBS-DMEM) to a final polymer concentration of 0.2 mg mL^{-1} and a final NR concentration of 2 μM. Samples were equilibrated for 1 h before characterization.

BTA-NR sample preparation. P4, **P4**_PW1, and **P4**_PW2 solutions were prepared via the standard procedures and the polymers were dried. To 0.5 mg polymer, BTA-NR was added

from a chloroform stock solution (0.15 mM) to a concentration of 1 molecule BTA-NR per polymer. Upon drying the samples, 0.25 mL PBS was added, and the samples were heated at 80 °C for 45 minutes and equilibrated overnight. Finally, the samples were diluted 2 times with either PBS or 20 vol% FBS in PBS (FBS-PBS) to a final polymer concentration of 1 mg mL^{-1} and BTA-NR concentration of 5.55 μM and equilibrated for 1 h before characterization.

Delivery of Nanoparticles to HeLa cells. HeLa cells were seeded in a μ-Slide 8-well (Ibidi) plate at a density of 25000 cells per well containing 200 μL of cell culture medium (DMEM supplemented with 10% FBS). The plate was then placed at 37 °C with 5% CO_2 flow in an oven for 24 h. After 24 h, the cell culture medium was replaced with DMEM supplemented with 10% FBS containing 2 mg mL^{-1} nanoparticles prepared by **P4** and **P4**_PW1. The plate was then placed back in the oven. After 24 h, the medium was discarded and PBS was added to wash the cells before taken confocal microscopy images.

Confocal Microscopy Imaging. Live cell images were taken by a Leica TCS SP5 AOBS equipped with a 63× water immersion objective. The excitation wavelength was set at 552 nm, and emission sequence images were collected from 570 to 770 nm using the high-sensitivity HyD detector under xyλ scan mode. The sequence images were plotted in Image J to show the emission spectra.

2. Small molecule synthesis and characterization

Synthesis of 7-ethoxy-4-methyl-coumarin (3):

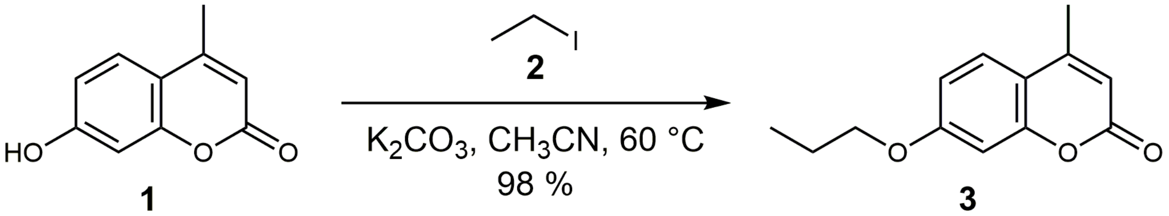

704.7 mg 7-hydroxy-4-methyl coumarin (**1**) (4.0 mmol, 1 eq) was dissolved in 30 mL of CH_3CN. 1.11 g K_2CO_3 (8.0 mmol, 2 eq.) and 0.64 mL ethyl iodide (**2**) (8.0 mmol, 2 eq.) were added. The reaction mixture was stirred at 60 °C for 48 h. After removal of the solvent, the crude product was washed with water and recrystallized from ethanol to obtain the desired compound (**3**) as a colorless solid with a yield of 800 mg (98%).

^1H NMR (400 MHz, $CDCl_3$): δ 7.49 (d, 1H, $^3J_{H,H}$ = 8.8 Hz), 6.85 (dd, 1H, $^3J_{H,H}$ = 8.8 Hz, $^4J_{H,H}$ = 2.5 Hz), 6.8 (d, 1H, $^4J_{H,H}$ = 2.5 Hz), 6.13 (d, 1H, $^4J_{H,H}$ = 0.8 Hz), 4.09 (q, 2H, $^3J_{H,H}$ = 7.0 Hz),

2.39 (d, 3H, $^4J_{H,H}$ = 0.8 Hz), 1.45 (t, 3H, $^3J_{H,H}$ = 7.0 Hz) ppm. ^{13}C NMR (100 MHz, $CDCl_3$): δ 162.2, 161.5, 155.5, 152.7, 125.6, 113.6, 112.8, 112.0, 101.5, 64.3, 18.8, 14.7 ppm. MALDI-TOF-MS: m/z calc.: 204.1; found: 205.0 (M + H^+), 227.0 (M + Na^+), 242.9 (M + K^+).

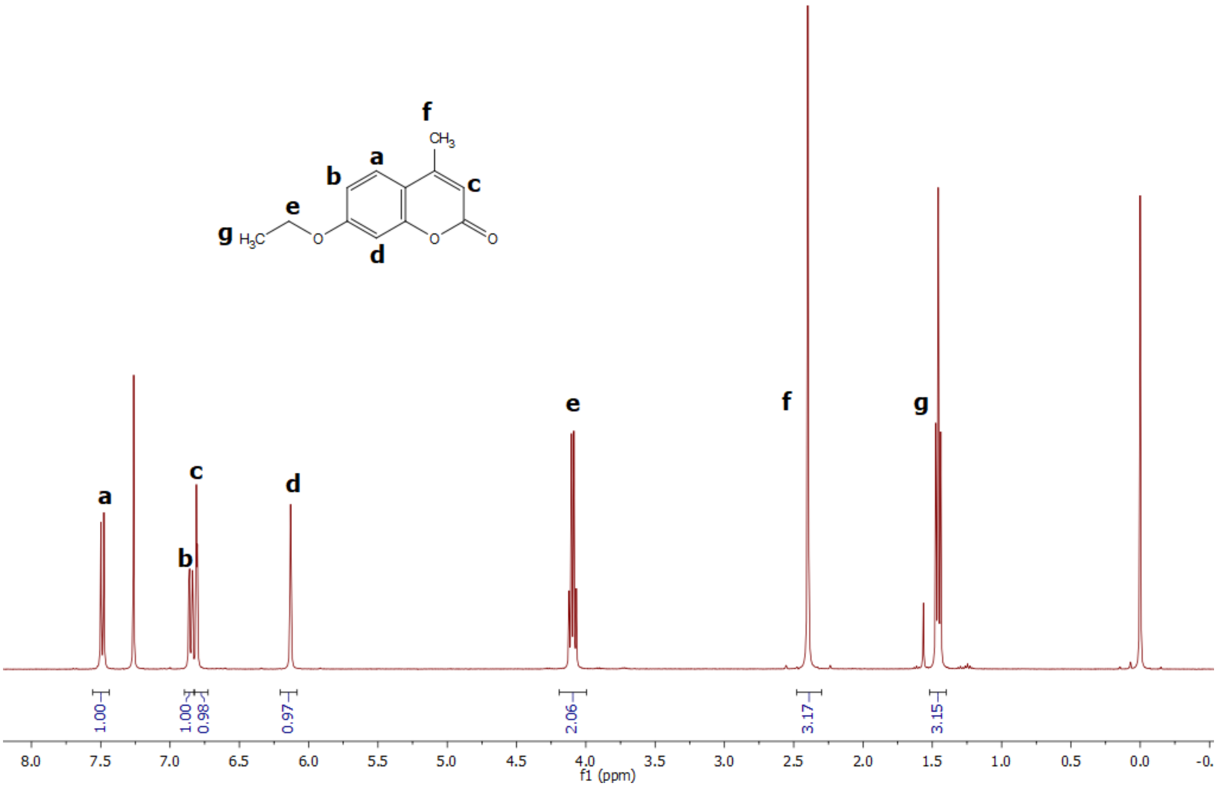

Figure S1: ^{1}H NMR spectrum of **3** in $CDCl_3$.

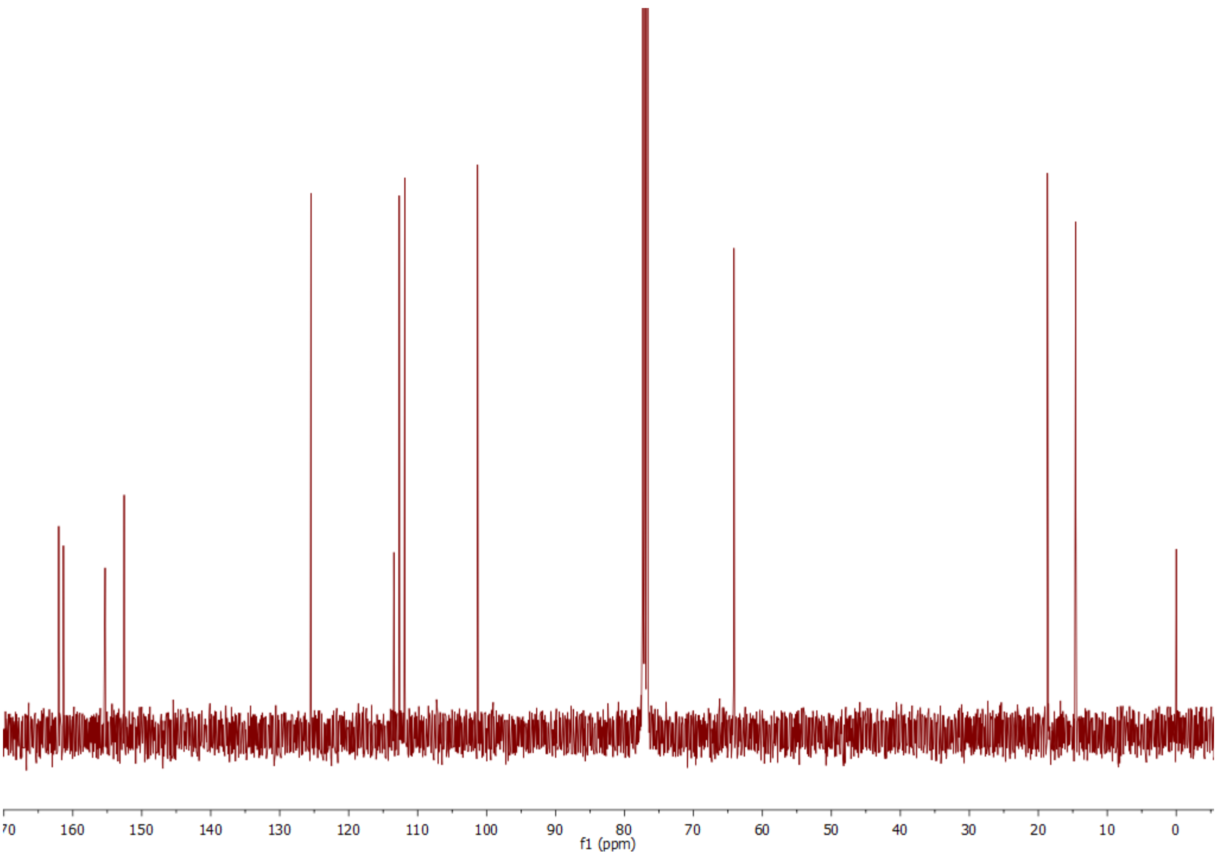

Figure S2: ^{13}C NMR spectrum of **3** in $CDCl_3$.

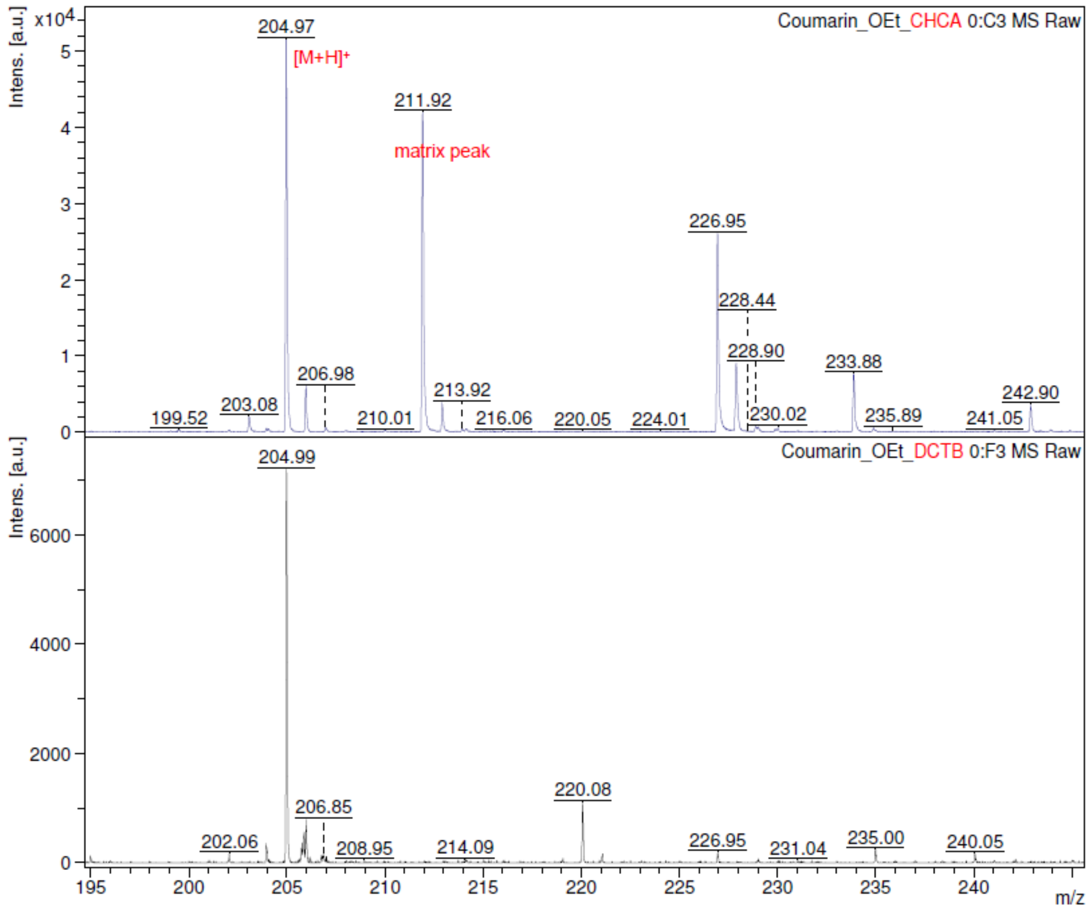

Figure S3: MALDI-TOF-MS spectrum of **3**.

Synthesis of 7-ethoxy-4-methyl-coumarin dimer (4):

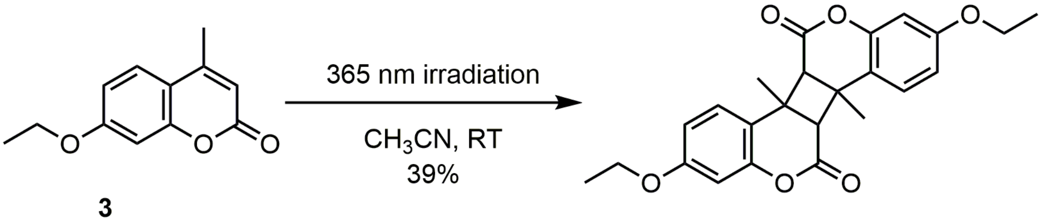

40.9 mg 7-ehoxy-4-methyl coumarin (**3**) (0.2 mmol) was dissolved in 2 mL of CH_3CN and illuminated with a 365 nm LED (Thorlabs) for 18 h while stirring rapidly. After removal of the solvent, the product (**4**)i was purified by flash column chromatography (SiO_2, heptane/ethyl acetate) and isolated as a colorless solid with a yield of 16 mg (39%).

^{1}H NMR (400 MHz, $CDCl_3$): δ 7.07 (d, 1H, $^3J_{H,H}$ = 8.7 Hz), 6.63 (d, 1H, $^3J_{H,H}$ = 8.7 Hz), 6.04 (s, 1H), 3.87 (m, 2H), 3.41 (s, 1H), 1.67 (s, 3H), 1.33 (t, 3H, $^3J_{H,H}$ = 6.9 Hz) ppm. ^{13}C NMR (100 MHz, $CDCl_3$): δ 164.8, 159.5, 150.3, 127.4, 113.6, 112.3, 102.3, 63.7, 55.3, 41.1, 31.6, 14.5 ppm.

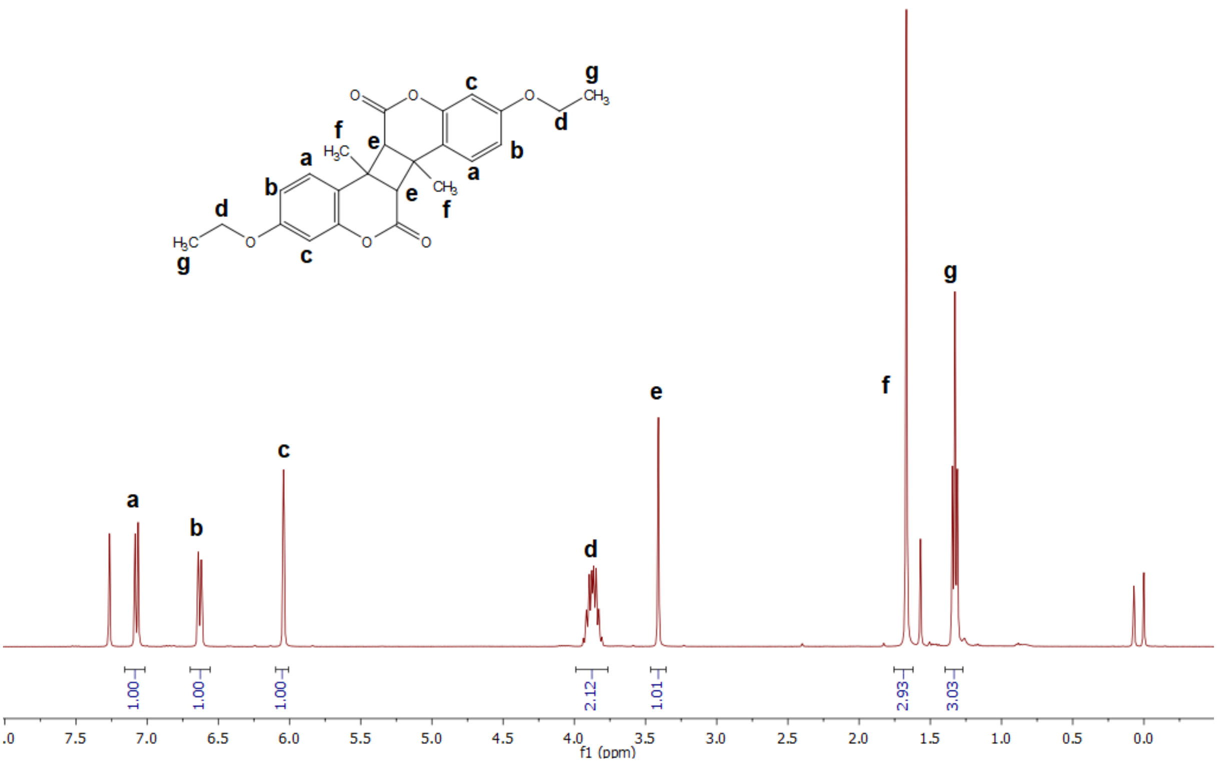

Figure S4: ^{1}H NMR spectrum of **4** in $CDCl_3$.

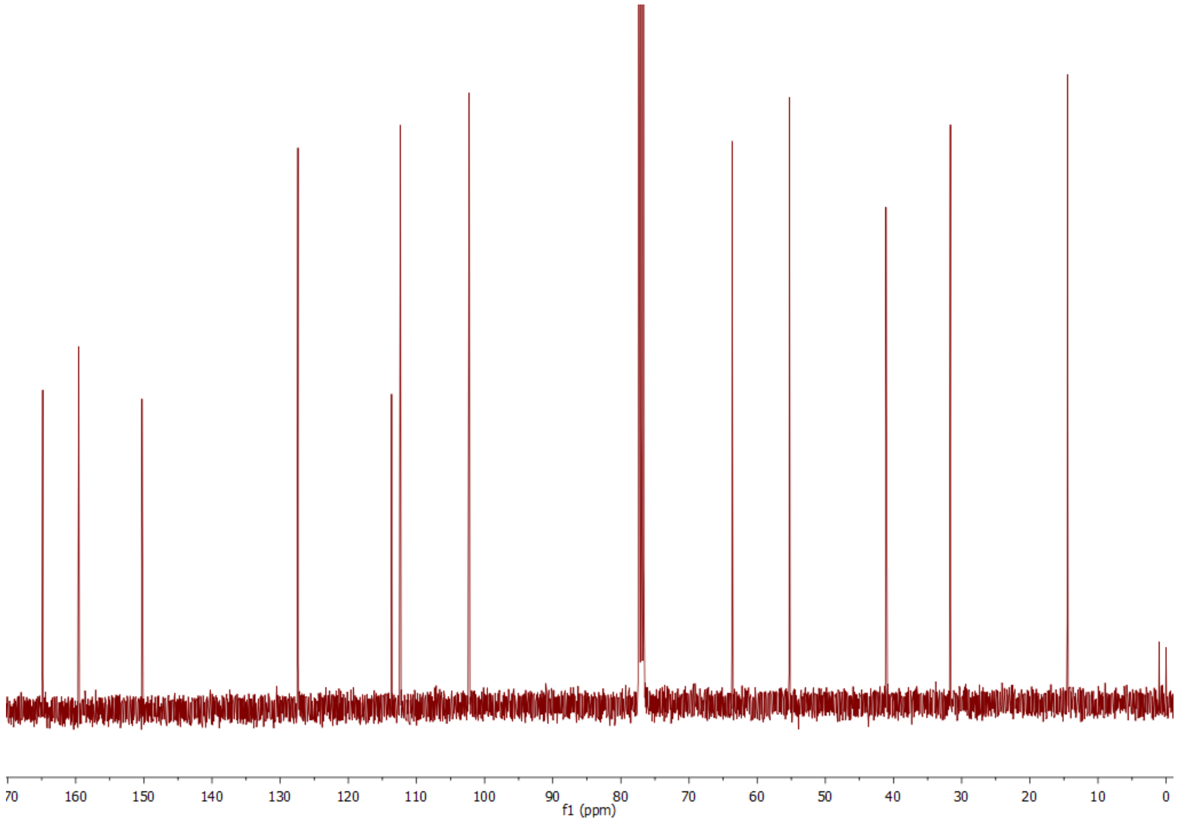

Figure S5: ^{13}C NMR spectrum of **4** in $CDCl_3$.

Characterization of (S,S)-BTA-C_{11}-amine (5):

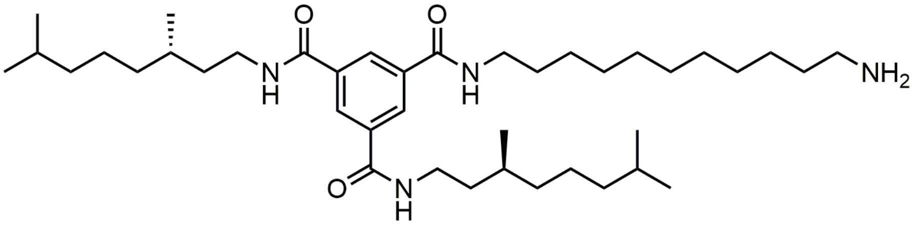

^{1}H NMR (400 MHz, $CDCl_3$): δ 8.41 – 8.25 (m, 3H), 6.93 – 6.72 (m, 3H), 3.55 – 3.33 (m, 6H), 2.73 (t, *J* = 7.1 Hz, 2H), 1.76 – 1.05 (m, 38H), 0.94 (d, *J* = 6.5 Hz, 6H), 0.86 (d, *J* = 6.6 Hz, 12H). FT-IR (ATR): n (cm^{-1}) = 3238, 3072, 2953, 2924, 2854, 1637, 1556, 1465, 1439, 1382, 1366, 1297, 1145, 906, 799, 729, 692.

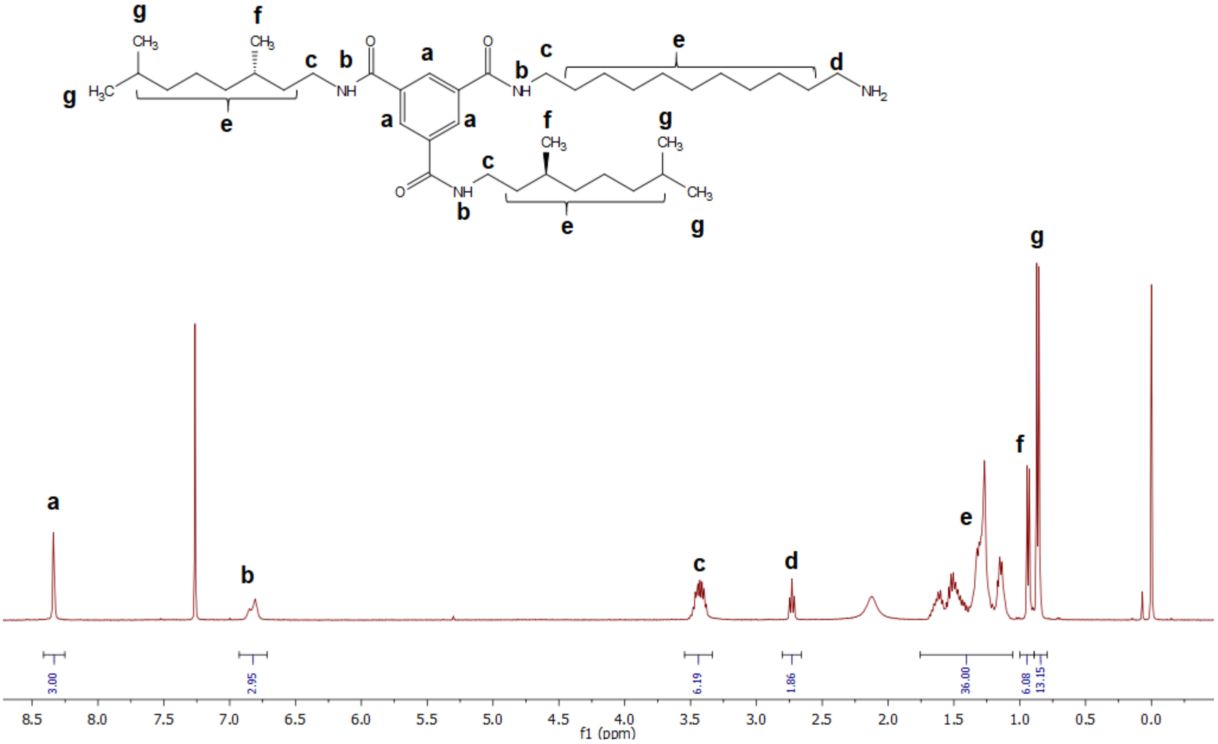

Figure S6: ^{1}H NMR spectrum of **5** in $CDCl_3$.

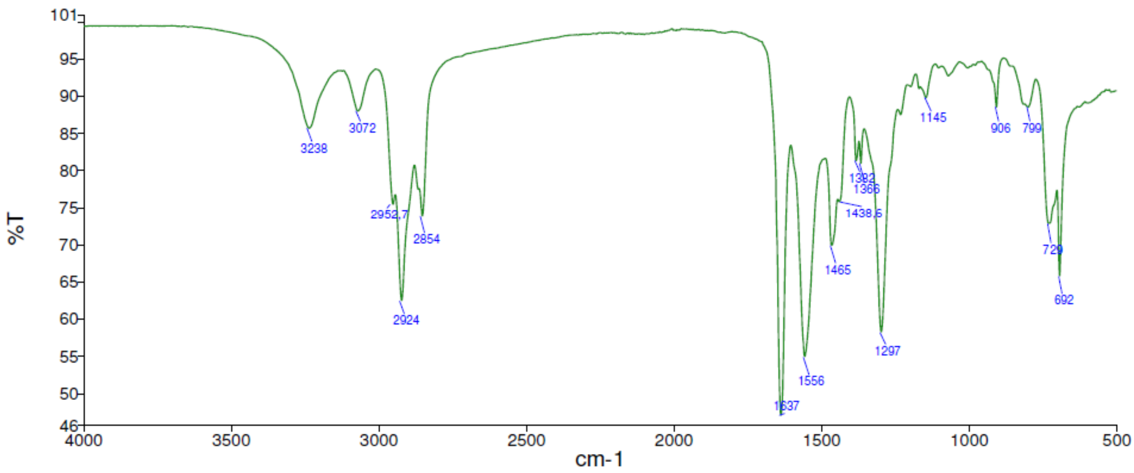

Figure S7: FT-IR spectrum in ATR mode of **5**.

(S,S)-BTA-NR (6):

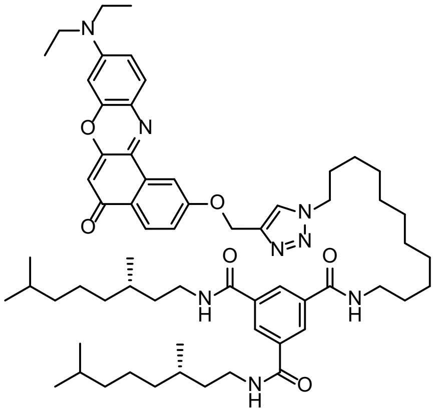

The full synthesis procedure and characterization of BTA-NR (**6**) by ^{1}H-NMR and MALDI-TOF-MS is provided in the recent publication of Linlin Deng *et al.*[2]

Characterization of 7-(2-aminoethoxy)-4-methyl-coumarin (TFA-salt) (7):

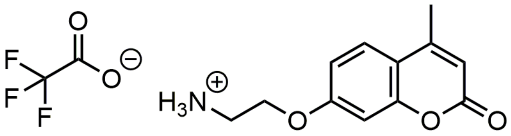

^{1}H NMR (400 MHz, DMSO-d_6): δ 8.10 (s, 3H), 7.73 (d, J = 8.6 Hz, 1H), 7.07 – 6.96 (m, 2H), 6.24 (s, 1H), 4.28 (t, J = 5.0 Hz, 2H), 3.27 (t, J = 5.0 Hz, 2H), 2.41 (s, 3H). FT-IR (ATR): n (cm^{-1}) = 3061, 1723, 1674, 1616, 1512, 1491, 1465, 1433, 1392, 1365, 1349, 1283, 1265, 1200, 1175, 1156, 1132, 1075, 1062, 1018, 998, 987, 880, 856, 836, 821, 797, 750, 721, 712, 680, 635, 593, 538, 518.

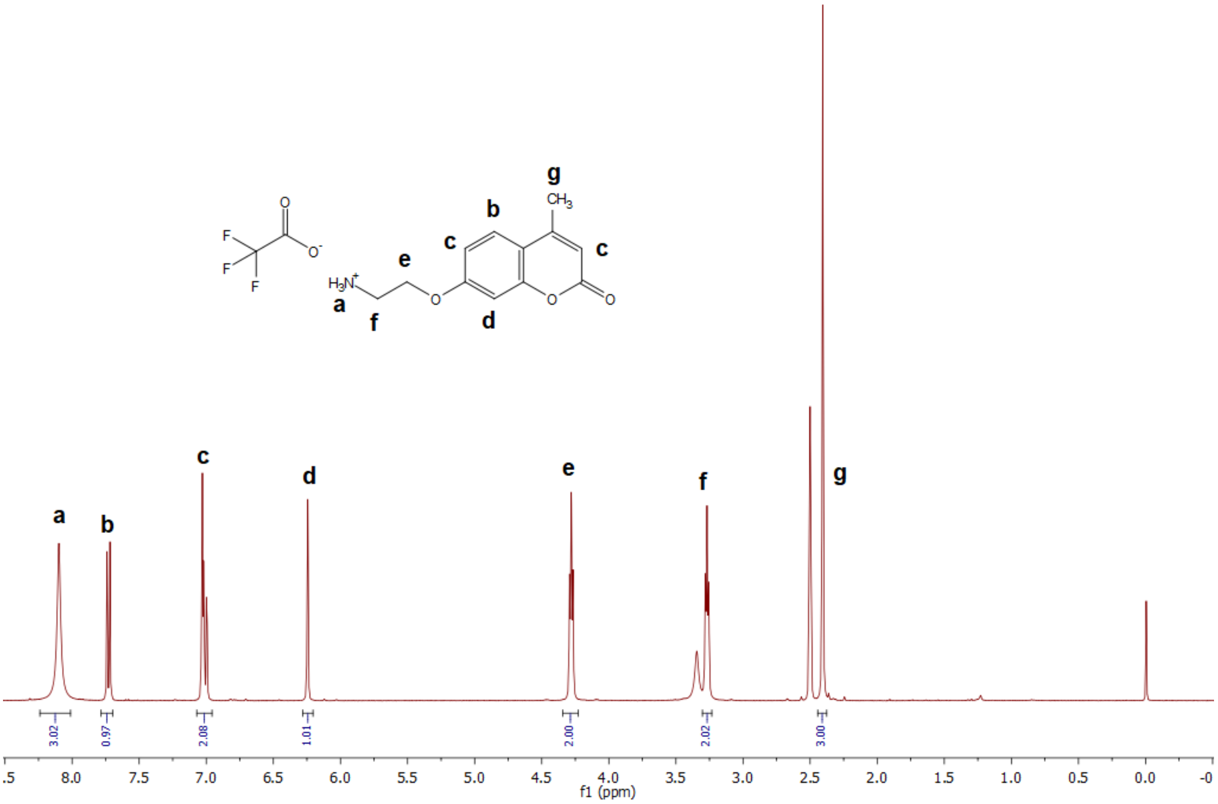

Figure S8: ^{1}H NMR spectrum of **7** in DMSO-d_6.

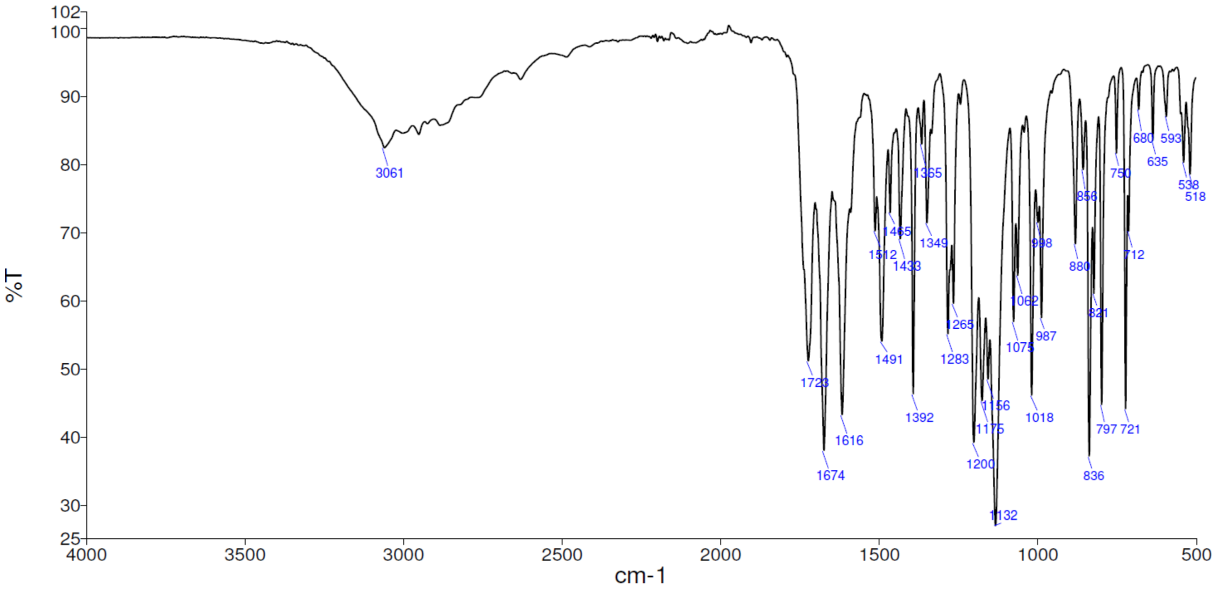

Figure S9: FT-IR spectrum of **7** in ATR mode.

3. Polymer synthesis and characterization

Synthesis of pentafluorophenyl acrylate (8):

10.12 g pentafluorophenol (54.4 mmol) was placed in an oven-dried 500 mL round-bottom flask with stirring bean and dissolved in 100 mL dry diethyl ether. The solution was placed under an argon atmosphere and 9.3 mL triethylamine (66.4 mmol, 1.2 eq.) was added. The flask was cooled using an ice bath before the dropwise addition of 5.5 mL acryloyl chloride (65.3 mmol, 1.2 eq.) in 50 mL dry diethylether while stirring. The solution turns white and opaque. Remove the icebath and stir overnight. Filter the reaction mixture (2x) and wash the filtrate with diethyl ether (20 mL) and remove the solvent *in vacuo* to obtain a bright yellow solution. Purify the crude product using column chromatography (silica, n-heptane) and evaporate the solvent *in vacuo* to obtain the product (**8**) as a slightly yellow oil. The obtained yield was 7.53 g (58%)

^{1}H NMR (399 MHz, $CDCl_3$): δ 6.72 (dd, J = 17.3, 1.1 Hz, 1H), 6.37 (dd, J = 17.3, 10.5 Hz, 1H), 6.18 (dd, J = 10.5, 1.1 Hz, 1H). ^{13}C NMR (100 MHz, $CDCl_3$): δ 161.69, 142.58, 140.82, 139.91, 139.23, 138.30, 136.72, 135.52, 125.38. ^{19}F NMR (376 MHz, $CDCl_3$): δ -152.35 – -152.78 (m), -157.93 (t, J = 21.7 Hz), -162.11 – -162.59 (m). FT-IR (ATR): n (cm^{-1}) = 2463, 1771,1655, 1634, 1515, 1472, 1406, 1293, 1218, 1147, 1111, 1070, 1030, 992, 980, 870, 797, 732, 694, 636, 618, 577, 548.

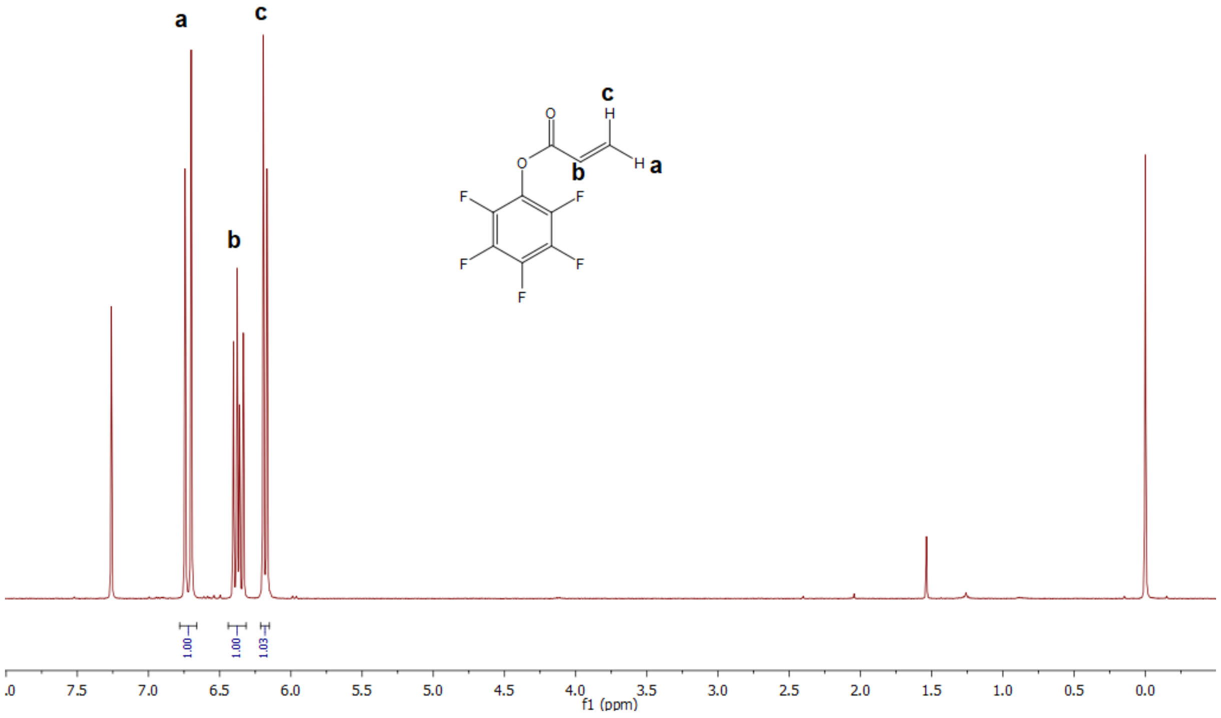

Figure S10: ^{1}H NMR spectrum of **8** in $CDCl_3$.

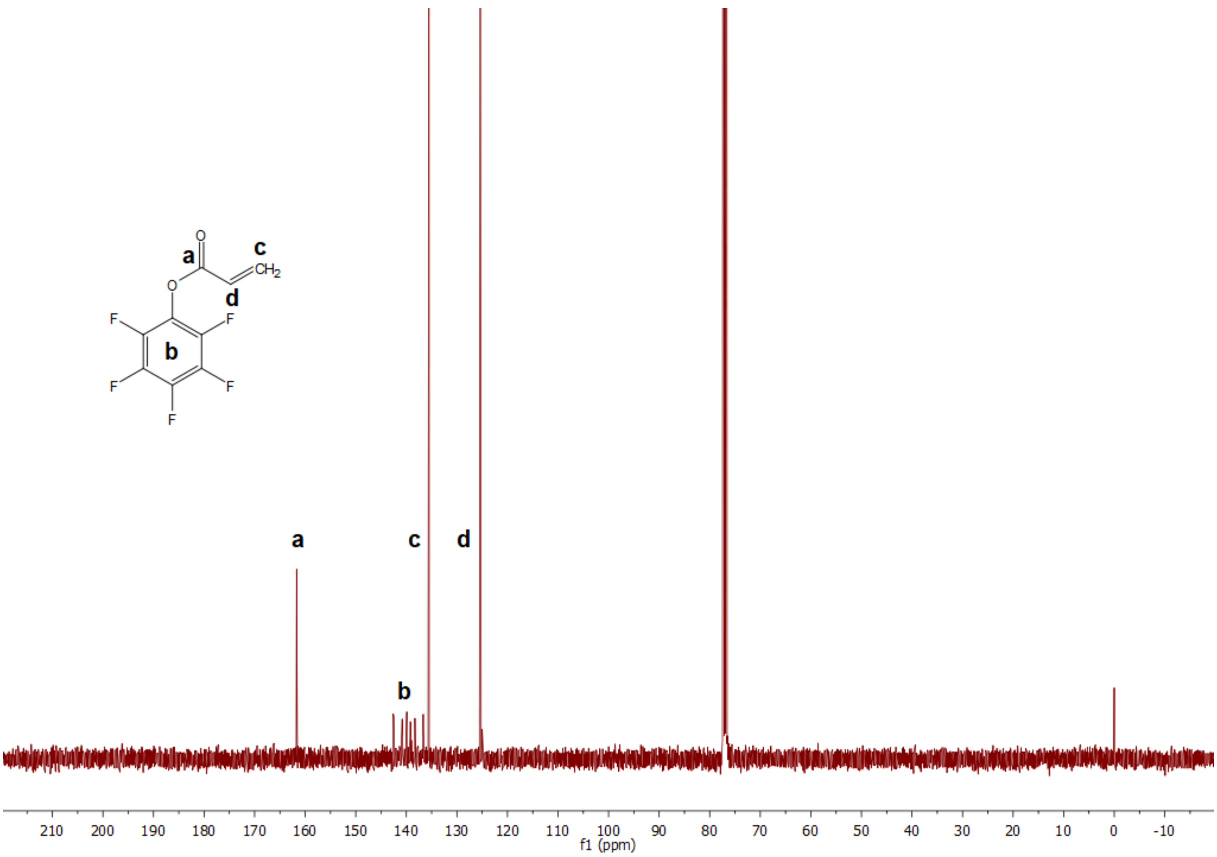

Figure S11: ^{13}C NMR spectrum of **8** in $CDCl_3$.

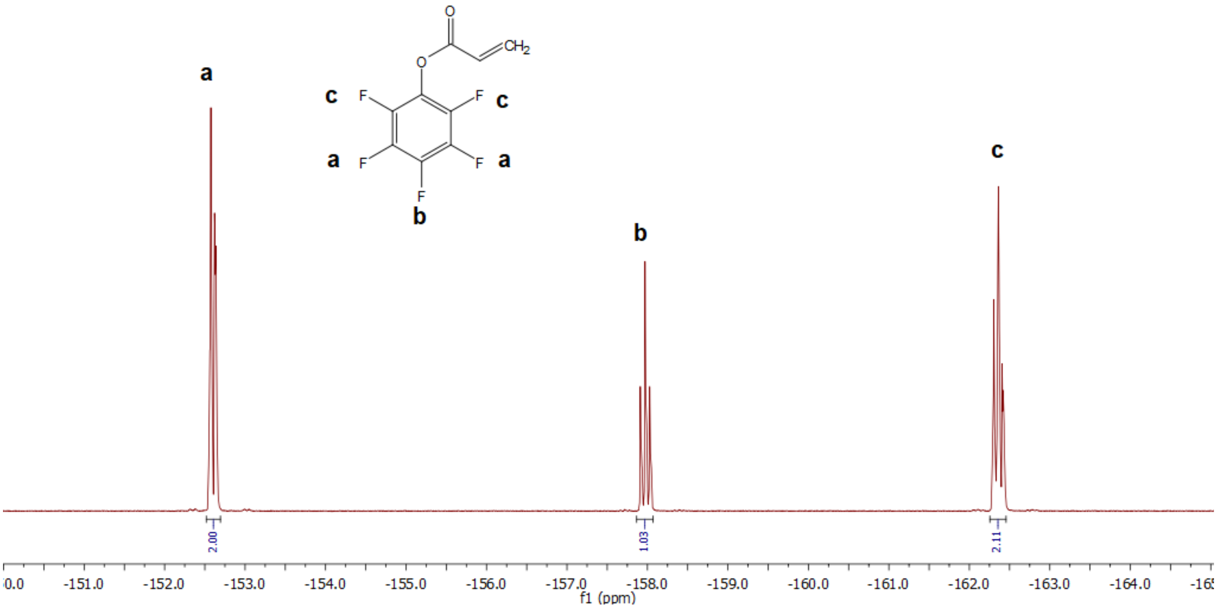

Figure S12: ^{19}F NMR spectrum of **8** in $CDCl_3$.

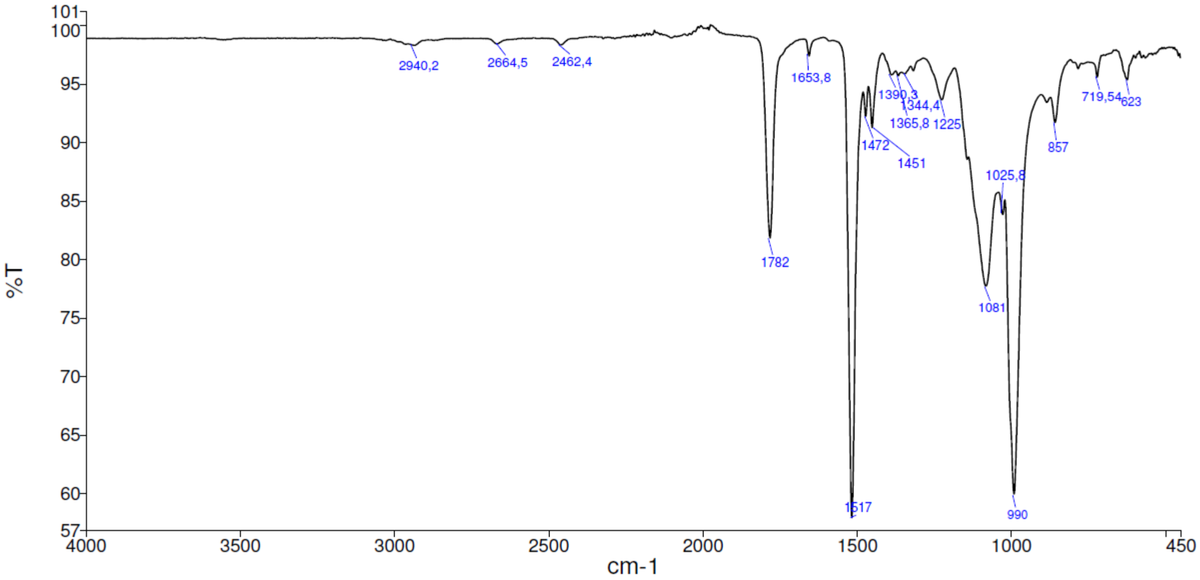

Figure S13: FT-IR spectrum of **8** in ATR mode.

Synthesis of poly(pentafluorophenyl acrylate) (9) (pPFPA):

3.107 g pentafluorophenyl acrylate (**8**) (13.05 mmol), 0.766 mg 2,2'-azobis(2-methylpropionitrile) (AIBN, 4.6 µmol, 0.0035 eq.), and 12.769 mg 4-cyano-4(phenylcarbonothioylthio)pentanoic acid (45.7 µmol, 0.00035 eq.) as chain transfer agent (RAFT agent) were dissolved in 0.8 mL anhydrous 1,4-dioxane and added to an oven-dried 50 mL round-bottom Schlenk flask with stirring bean placed under an argon atmosphere. The vials were washed twice with an additional 2 mL of anhydrous 1,4-dioxane, which was added to the Schlenk flask. The solution was degassed by argon bubbling for 45 minutes while stirring the solution. The Schlenk flask was kept under argon atmosphere and placed in a preheated oil bath at 80 °C. The polymerization reaction was monitored by ^{19}F NMR and the reaction was quenched by placing the reaction mixture in liquid nitrogen after 135 minutes. After thawing of the reaction mixture, the final conversion of the resulting viscous pink liquid was determined to be 65% by ^{19}F NMR (Figure S14). The reaction mixture was dissolved in DCM at 40 °C and precipitated in 800 mL old n-pentane (washed three times) to obtain the target compound (**9**) as a fluffy light pink powder after evaporation of the solvent in vacuo. The successful removal of residual monomer was confirmed by ^{19}F NMR (Figure S16). The obtained yield was 1.84 g (91% w.r.t. conversion). The degree of polymerization DP was calculated as $DP = [M_{PFPA}] / [M_{RAFT}] \, x \, conversion$ = 13.05 / 0.046 x 0.65 = 186, with M_{PFPA} and M_{RAFT} the molarity of the PFPA monomer and RAFT agent respectively. The theoretical number-average molecular weight was calculated as $M_{n,th} = DP \, x \, M_{w,PFPA} + M_{w,RAFT}$ = 186 x 238.11 + 279.37 = 44.6 kDa, with $M_{w,PFPA}$ and $M_{w,RAFT}$ the molecular weight of PFPA and the RAFT agent respectively.

^{1}H NMR (399 MHz, $CDCl_3$): δ (ppm) = 3.09 (br), 2.49 (br), 2.06 (br). ^{19}F NMR (376 MHz, $CDCl_3$): δ (ppm) = -153.24 (br), -156.78 (br), -162.20 (br). SEC (THF): M_n = 34.4 kDa, Đ = 1.18 (RI detector). FT-IR (ATR): n (cm^{-1}) = 1782, 1655, 1516, 1472, 1451, 1389, 1366, 1346, 1227, 1080, 1026, 990, 886, 857, 783, 720, 623.

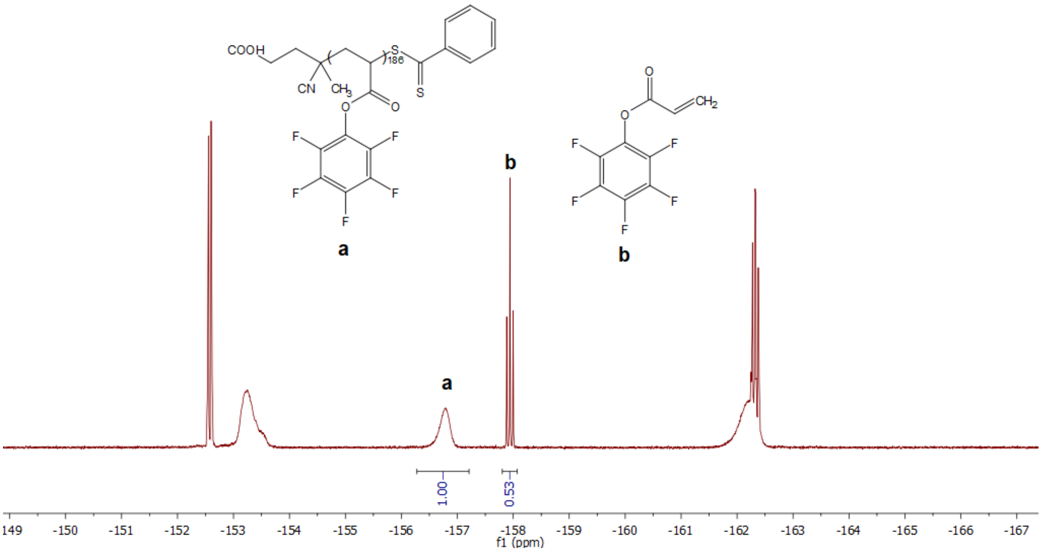

Figure S14: ^{19}F NMR spectrum to determine the conversion of **8** in $CDCl_3$ after polymerization. Conversion is 65%.

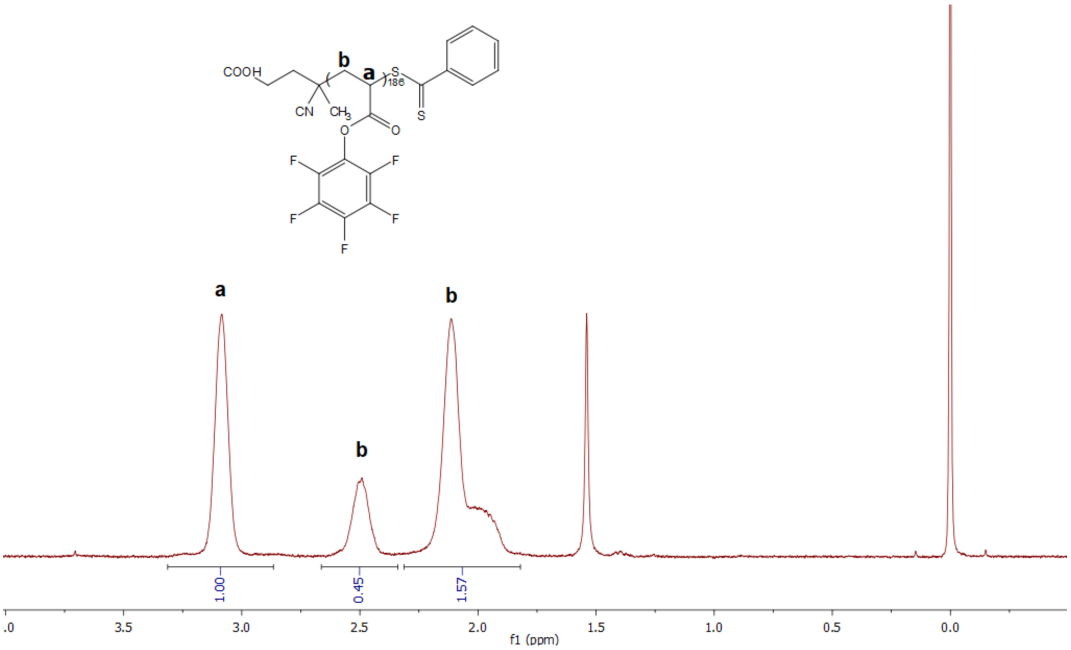

Figure S15: ^{1}H NMR spectrum of **9** in $CDCl_3$.

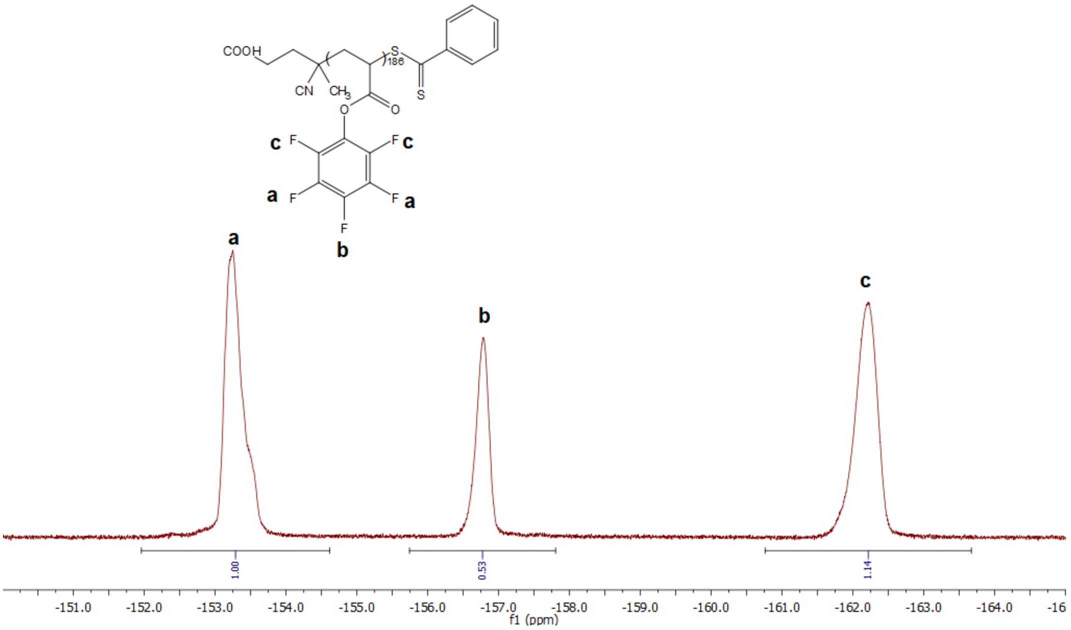

Figure S16: ^{19}F NMR spectrum of **9** in $CDCl_3$.

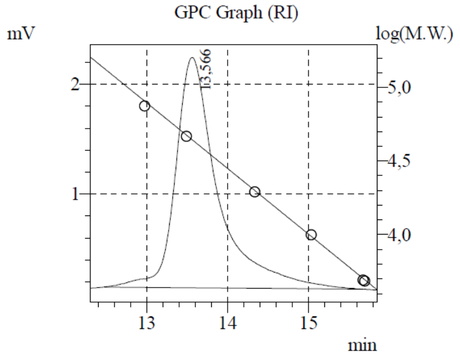

Figure S17: SEC trace of **9** in THF.

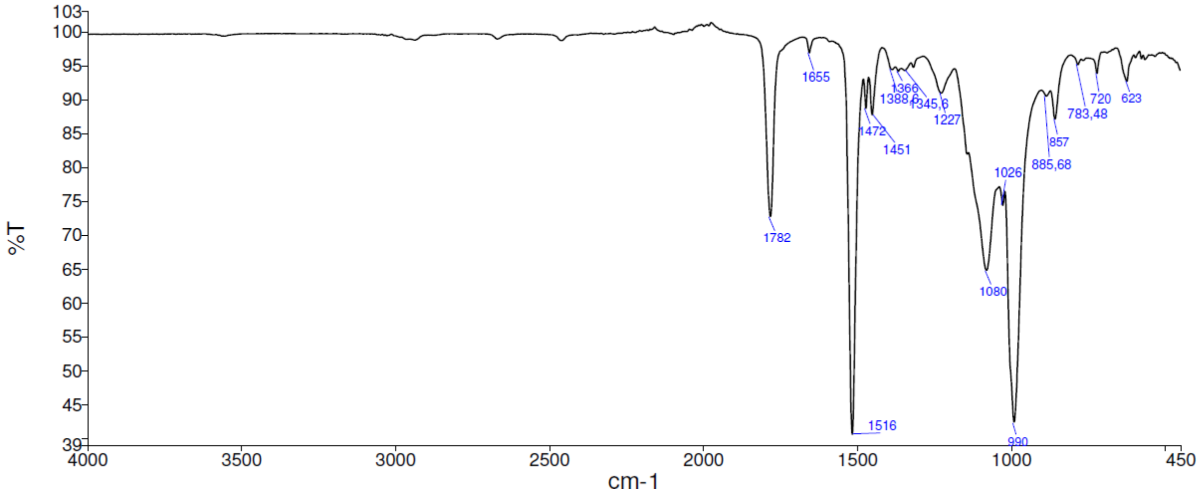

Figure S18: FT-IR spectrum of **9** in ATR mode.

End-group modification of poly(pentafluorophenyl acrylate) (10):

1.7774 g pPFPA (**9**) (0.040 mmol, 1 eq.), 133.77 mg AIBN (0.80 mmol, 20 eq.), and 32.86 mg lauroyl peroxide (0.080 mmol, 2 eq.) were weighed into vials and added to an oven-dried, 50 mL round-bottom Schlenk flask in addition to a stirring bean, which was kept under an argon atmosphere. The vials were washed four times with 1 mL anhydrous 1,4-dioxane and added to the Schlenk flask. An additional 9 mL anhydrous 1,4-dioxane was added to the Schlenk flask and the solids were dissolved by stirring to give a transparent pink solution. Four freeze-pump-thaw cycles were performed to degas the solution after which the Schlenk was kept under an argon atmosphere and placed in a preheated 80 °C oil bath. The solution turned colorless after 1 hour. The reaction was quenched by placing the reaction mixture in liquid nitrogen after 135 minutes. Evaporate the solvent *in vacuo* to end up with a transparent / white solid. Dissolve the crude product in 6 mL DCM and precipitate in 800 mL cold n-hexane (washed three times). Repeat the precipitation from DCM in 1 L cold n-pentane (washed three times). Evaporate the solvent *in vacuo* and remove solvent traces using a high-vacuum line. The polymer (**10**) was obtained as a white powder with a yield of 1293 mg (73%). The theoretical number-average molecular weight was calculated as $M_{n,th} = DP \times M_{w,PFPA} + M_{w,endgroup}$ = 186 x 238.11 + 194.23 = 44.5 kDa.

^{1}H NMR (399 MHz, $CDCl_3$): δ (ppm) = 3.08 (br), 2.49 (br), 2.11 (br). ^{19}F NMR (376 MHz, $CDCl_3$): δ (ppm) = -152.58 – -153.90 (br), -156.12 – -157.44 (br), -161.54 – -162.86 (br). SEC (THF): M_n = 37.6 kDa, Đ = 1.14 (RI detector). FT-IR (ATR): n (cm^{-1}) = 1782, 1655, 1515, 1472, 1451, 1387, 1366, 1345, 1315, 1227, 1079, 1027, 989, 884, 857, 782, 720, 623.

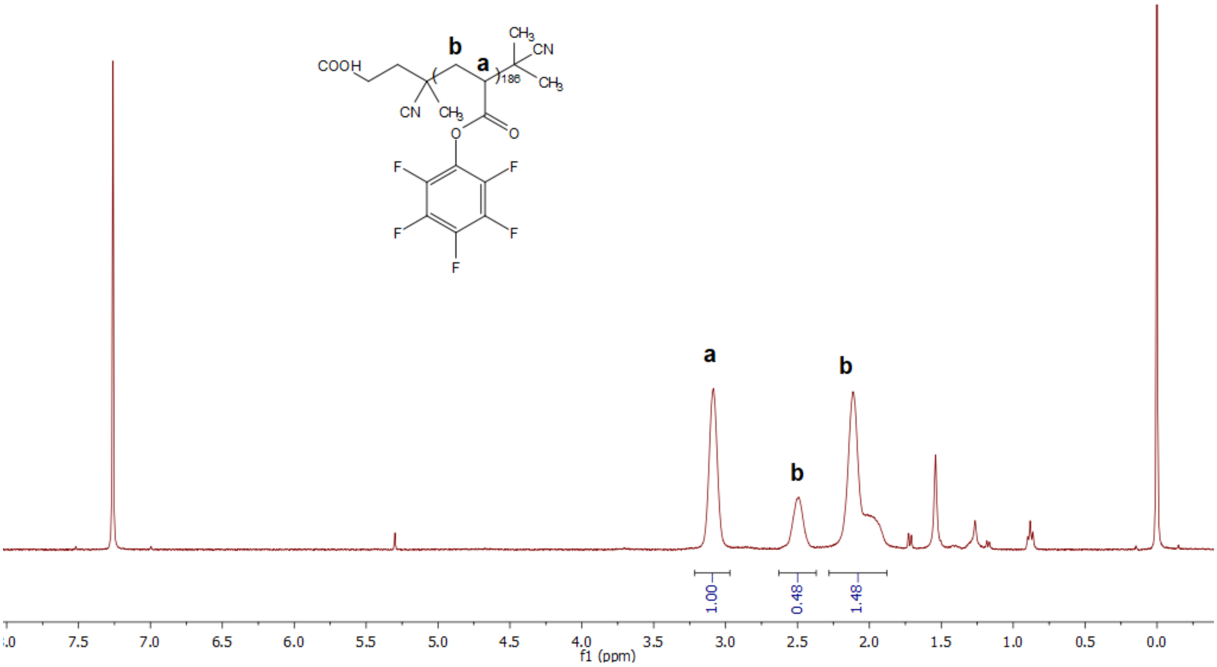

Figure S19: ^{1}H NMR spectrum of **10** in $CDCl_3$.

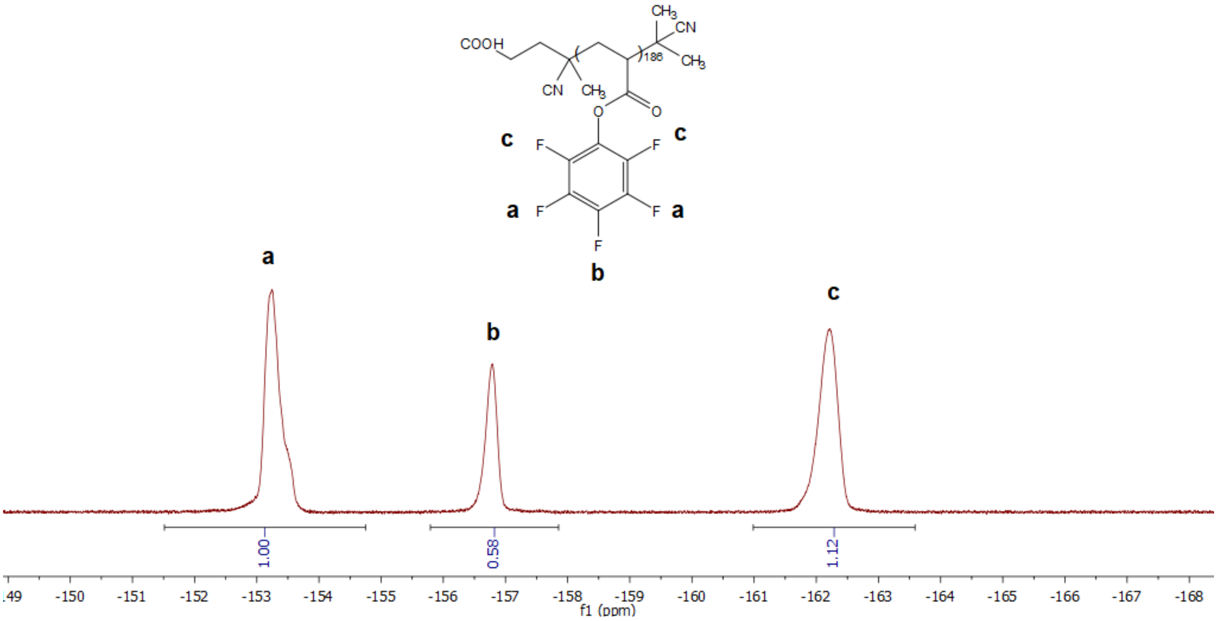

Figure S20: ^{19}F NMR spectrum of **10** in $CDCl_3$.

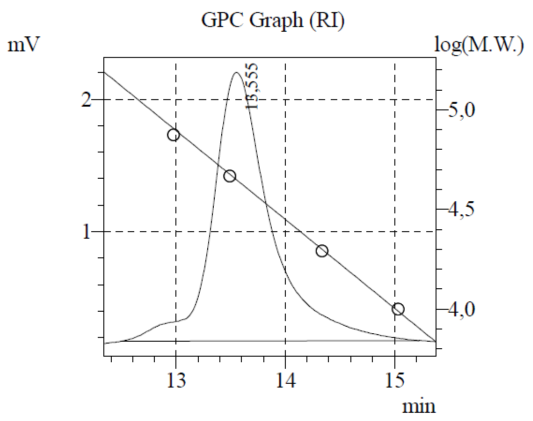

Figure S21: SEC trace of **10** in THF.

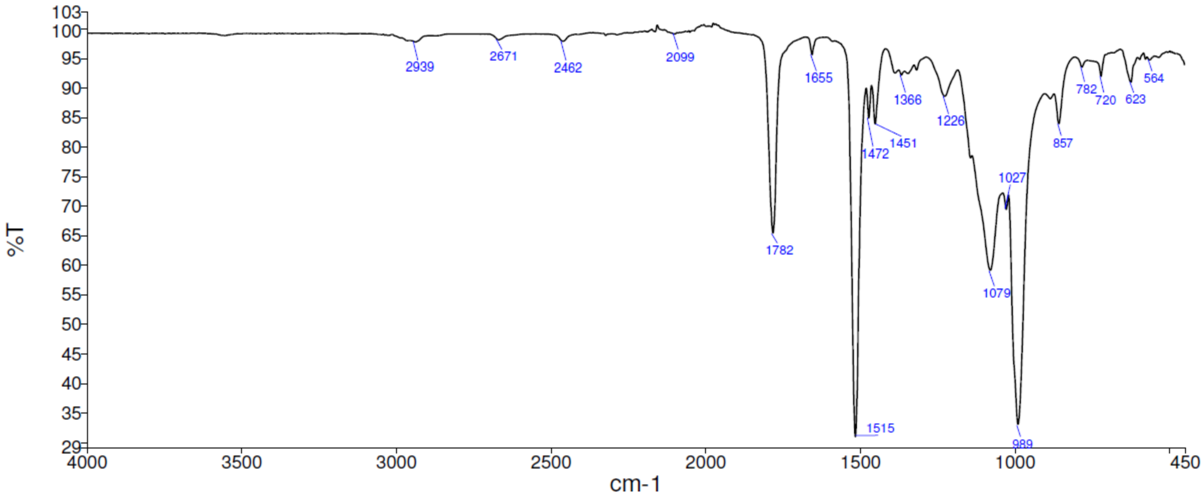

Figure S22: FT-IR spectrum of **10** in ATR mode.

Amine post-functionalization of poly(pentafluorophenyl acrylate) (P1-P7):

The synthesis of **P1** to **P7** from pPFPA (**10**) followed a sequential amine post-functionalization procedure. The synthesis of **P4** is given below as illustration. The incorporation of amine was monitored by periodically taking an aliquot of the reaction mixture and using ^{19}F NMR in $CDCl_3$ to determine the ratio of pPFPA to free pentafluorophenol (Figure S23). The NMR sample was evaporated using a nitrogen stream and returned to the reaction mixture in 0.5 mL dry THF. In this way, the amount of polymer in the reaction mixture remained constant throughout the functionalization. In all cases, the amine incorporation ratio agreed well with the feed ratio, see Table S1.

175.7 mg (3.9 µmol, 1 eq.) end-group modified pPFPA (**9**), 12.27 mg (36.6 µmol, 5 eq.) coumarin amine (TFA-salt) (**7**) were weighed into vials and, together with 6.12 µL triethylamine (36.6 µmol, 5 eq.) added to an oven-dried, 50 mL round-bottom Schlenk flask charged with a stirring bean, which was kept under an argon atmosphere. The vials were washed with 5 mL dry THF and added to the Schlenk flask. The Schlenk flask was lowered into a

preheated oil bath at 50 °C under continuous stirring. Functionalization took 3 hours, after which 24.06 mg (36.6 µmol, 5 eq.) S,S-BTA-C_{11}-amine (**5**) in 1 mL dry THF was added to the Schlenk flask. Functionalization took 5 hours, after which 13.90 mg (73.2 µmol, 10 eq.) dodecylamine in 1 mL THF was added to the Schlenk flask and stirred overnight. 1.6 g (1.56 mmol, 372 eq., 1.5x excess) Jeffamine was dried over P_2O_5 at 50 °C overnight. The next day, the Jeffamine was added as liquid to the reaction mixture directly from the oven. After 2.5 hours, the functionalization was complete. The final reaction mixture was a transparent yellow solution. The reaction mixture was dialyzed twice against 1 L THF for a day each, followed by dialysis twice against 1 L MeOH for a day each. The reaction mixture after dialysis was almost colorless. The solvent was evaporated *in vacuo* and the resulting sticky solid was dissolved in minimum DCM. The polymer was precipitated in 800 mL cold n-pentane as a sticky while solid (washed two times). The polymer was dissolved in DCM and transferred to a sample vial, upon which the solvent was removed *in vacuo* at 50°C. The polymer (**P4**) was obtained as a slightly yellowish solid with a yield of 304 mg (44%).

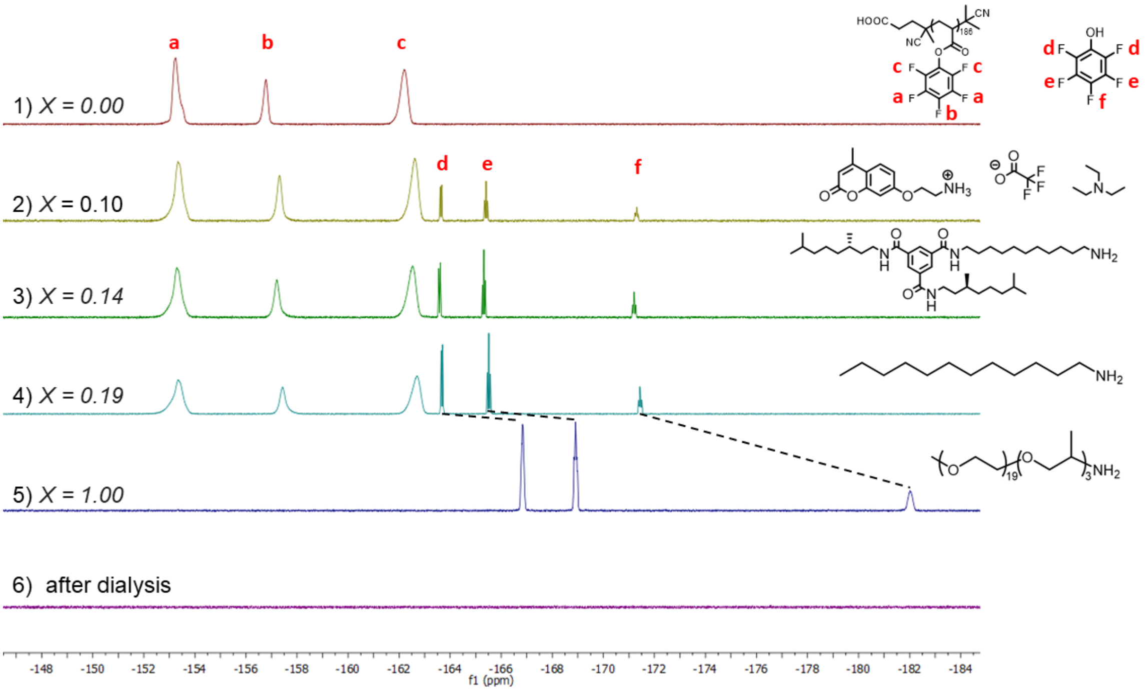

Figure S23: ^{19}F NMR spectra of 1) pPFPA$_{186}$ in $CDCl_3$, and after amine post-functionalization with 2) coumarin-amine, 3) S,S-BTA-C_{11}-amine, 4) dodecylamine, and 5) Jeffamine. 6) All pentafluorophenol was removed after dialysis. This spectra corresponds to polymer **P5**. *X* is the conversion calculated from the integrated signals according to X = d / (c + d).

Table S1: Incorporation ratios of the different grafts of polymers **P1** – **P7** as determined by ^{19}F-NMR during the amine post-functionalization procedure. Deviations from the feed ratio are given between brackets.

**Polymer**	**BTA amine**	**Coumarin amine**	**Dodecyl amine**	**Jeffamine**
**P1**	0	0	0.20	0.8
**P2**	0	0.10	0.10	0.8
**P3**	0.04 (-0.01)	0	0.15	0.81
**P4**	0.04 (-0.01)	0.05	0.10	0.81
**P5**	0.04 (-0.01)	0.10	0.05	0.81
**P6**	0.04 (-0.01)	0.15	0	0.81
**P7**	0	0	0	1.00

Synthesis of P1:

^{1}H NMR (399 MHz, $CDCl_3$): δ (ppm) = 6.68 (br), 4.95 – 2.76 (m), 2.76 – 0.29 (m). SEC (DMF): M_n = 56 kDa, Đ = 1.28 (RI detector). FT-IR (ATR): n (cm^{-1}) = 3315, 2869, 1648, 1535, 1466, 1344, 1280, 1242, 1103, 963, 843, 527. Yield = 336 mg (50%).

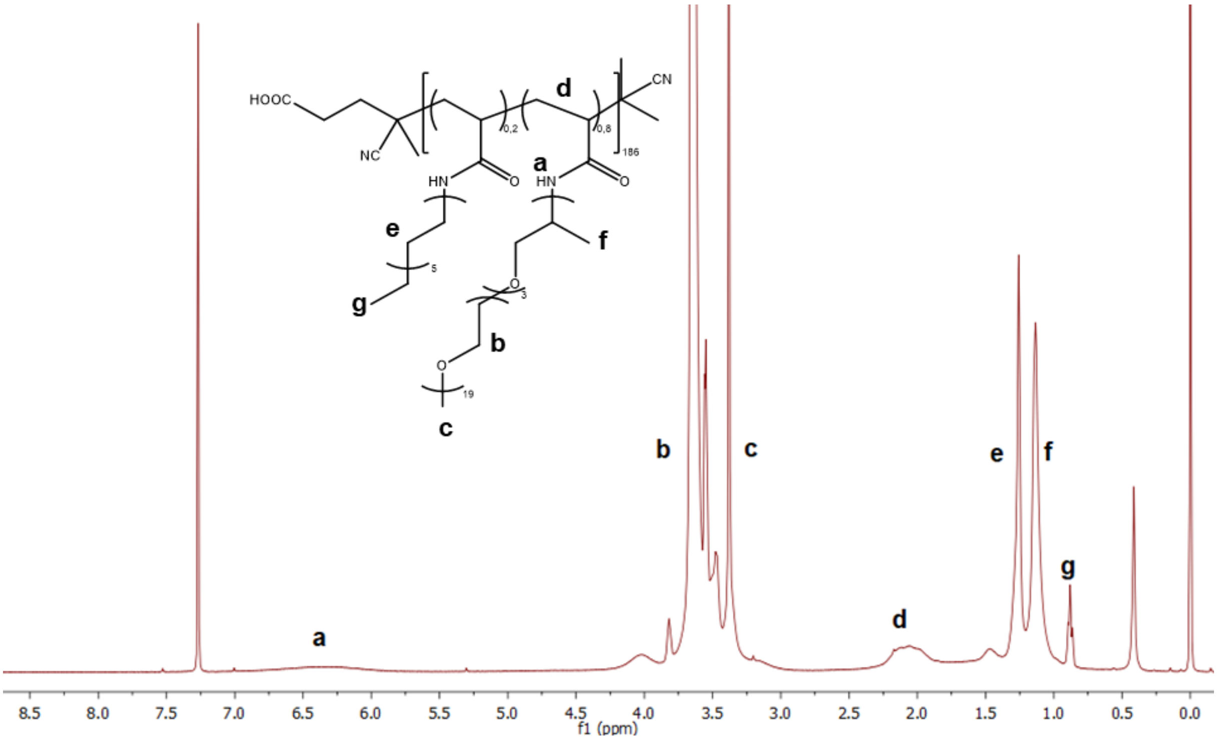

Figure S24: ^{1}H NMR spectrum of **P1** in $CDCl_3$.

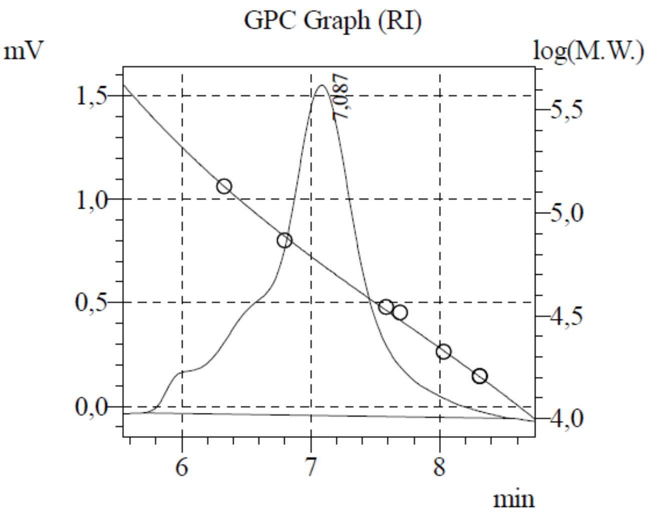

Figure S25: SEC trace of **P1** in DMF.

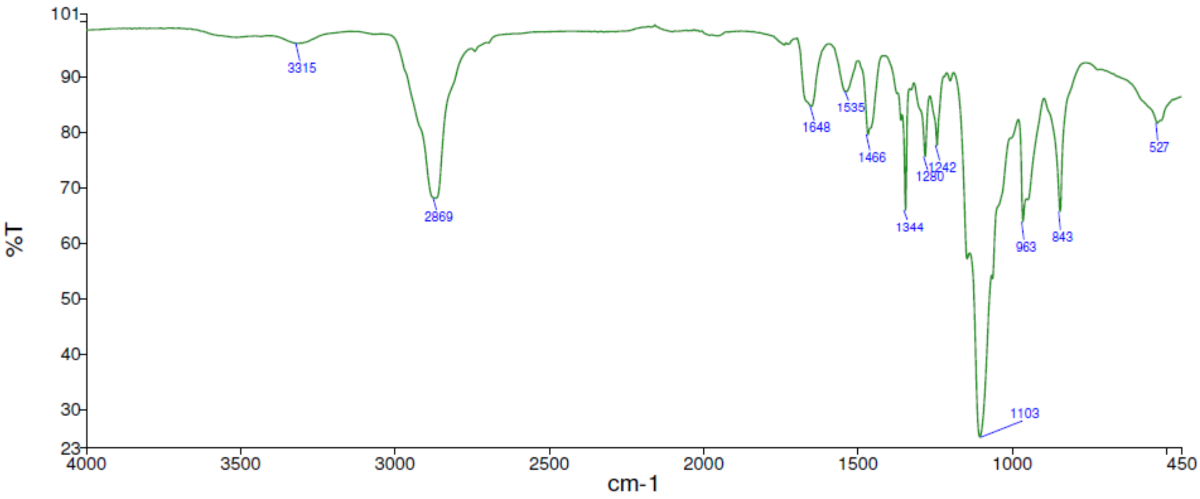

Figure S26: FT-IR spectrum of **P1** in ATR mode.

Synthesis of P2:

^{1}H NMR (399 MHz, $CDCl_3$): δ (ppm) = 7.83 – 5.68 (m), 5.03 – 2.82 (m), 2.81 – 0.27 (m). SEC (DMF): M_n = 55 kDa, Đ = 1.35 (RI detector). FT-IR (ATR): n (cm^{-1}) = 3315, 2867, 1726, 1649, 1535, 1466, 1344, 1281, 1242, 1102, 963, 843, 527. Yield = 273 mg (40%).

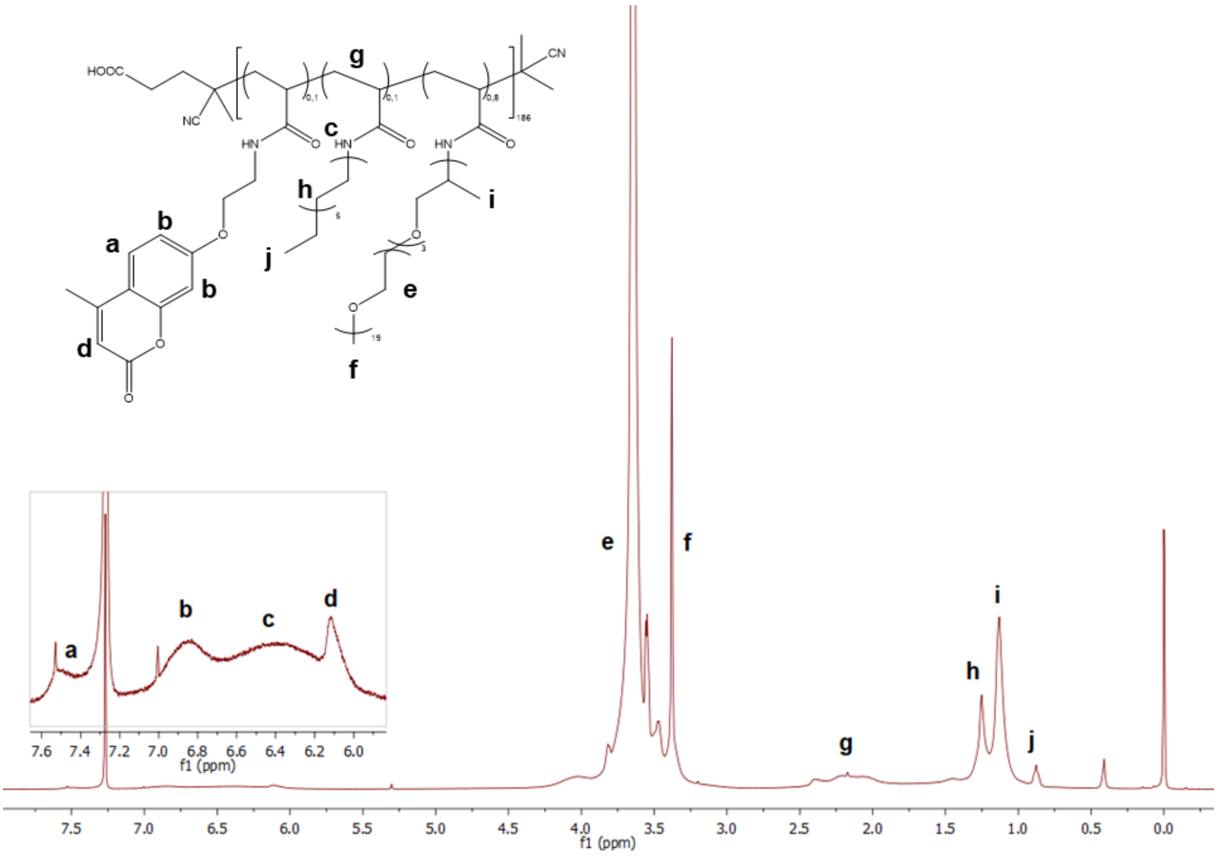

Figure S27: ^{1}H NMR spectrum of **P2** in $CDCl_3$.

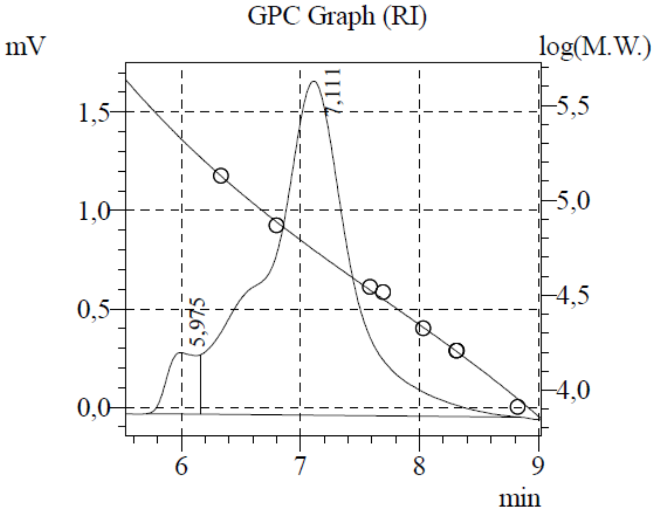

Figure S28: SEC trace of **P2** in DMF.

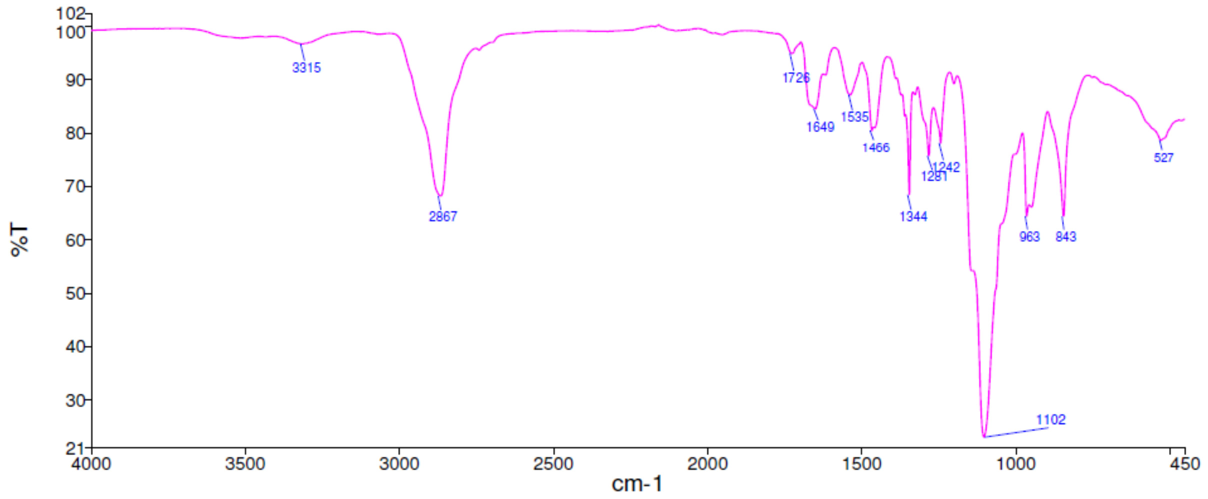

Figure S29: FT-IR spectrum of **P2** in ATR mode.

Synthesis of P3:

^{1}H NMR (399 MHz, $CDCl_3$): δ (ppm) = 8.46 (s), 7.00 (br), 4.94 – 2.80 (m), 2.80 – 0.24 (m). SEC (DMF): M_n = 51 kDa, Đ = 1.27 (RI detector). FT-IR (ATR): n (cm^{-1}) = 3317, 2867, 1649, 1536, 1466, 1344, 1281, 1242, 1102, 963, 843, 526. Yield = 304 mg (44%).

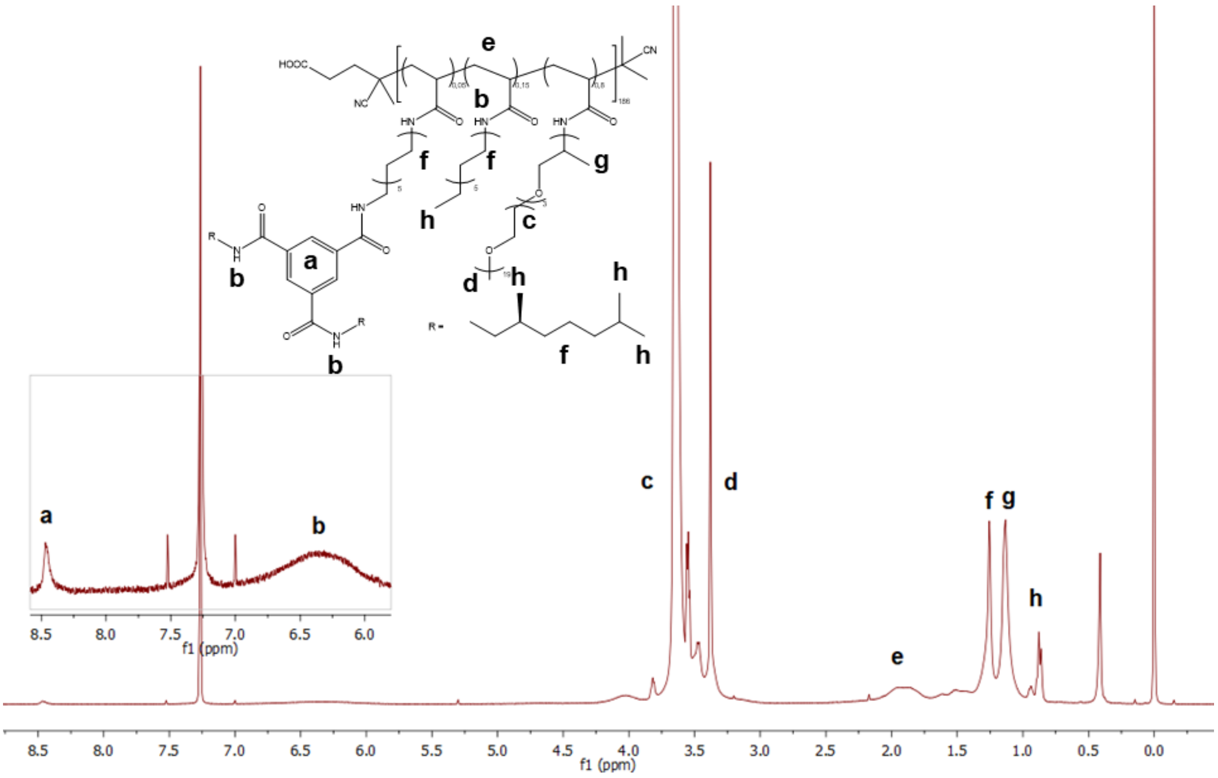

Figure S30: ^{1}H NMR spectrum of **P3** in $CDCl_3$.

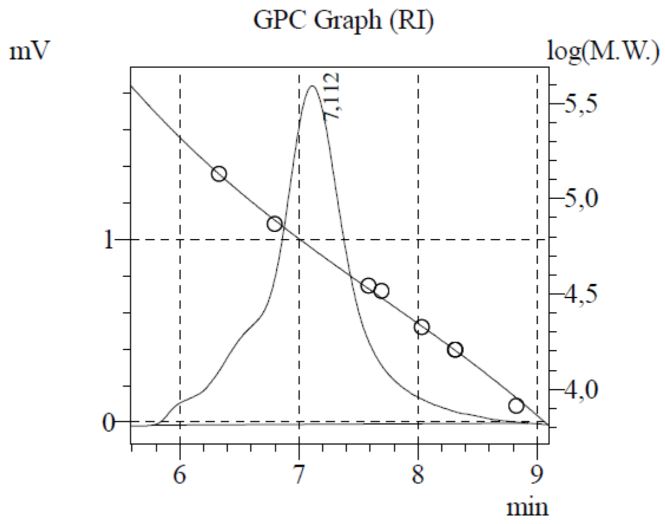

Figure S31: SEC trace of **P3** in DMF.

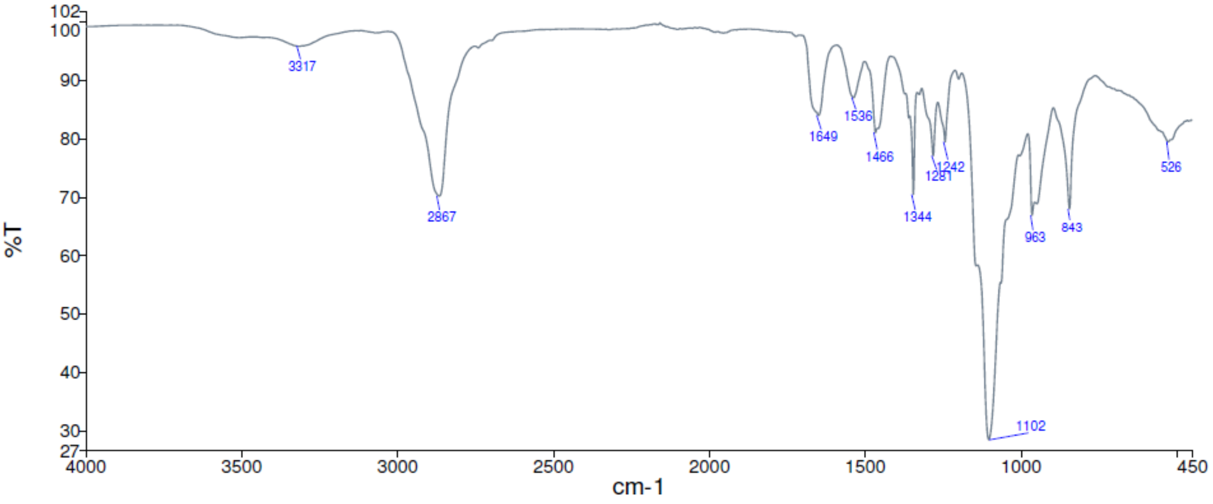

Figure S32: FT-IR spectrum of **P3** in ATR mode.

Synthesis of P4:

^{1}H NMR (399 MHz, $CDCl_3$): δ (ppm) = 8.46 (s), 8.00 – 5.62 (m), 5.58 – 2.83 (m), 2.83 – 0.22 (m). SEC (DMF): M_n = 59.0 kDa, Đ = 1.35 (RI detector). FT-IR (ATR): n (cm^{-1}) = 3315, 2869, 1648, 1536, 1466, 1344, 1280, 1242, 1101, 963, 843, 527. Yield = 304 mg (44%).

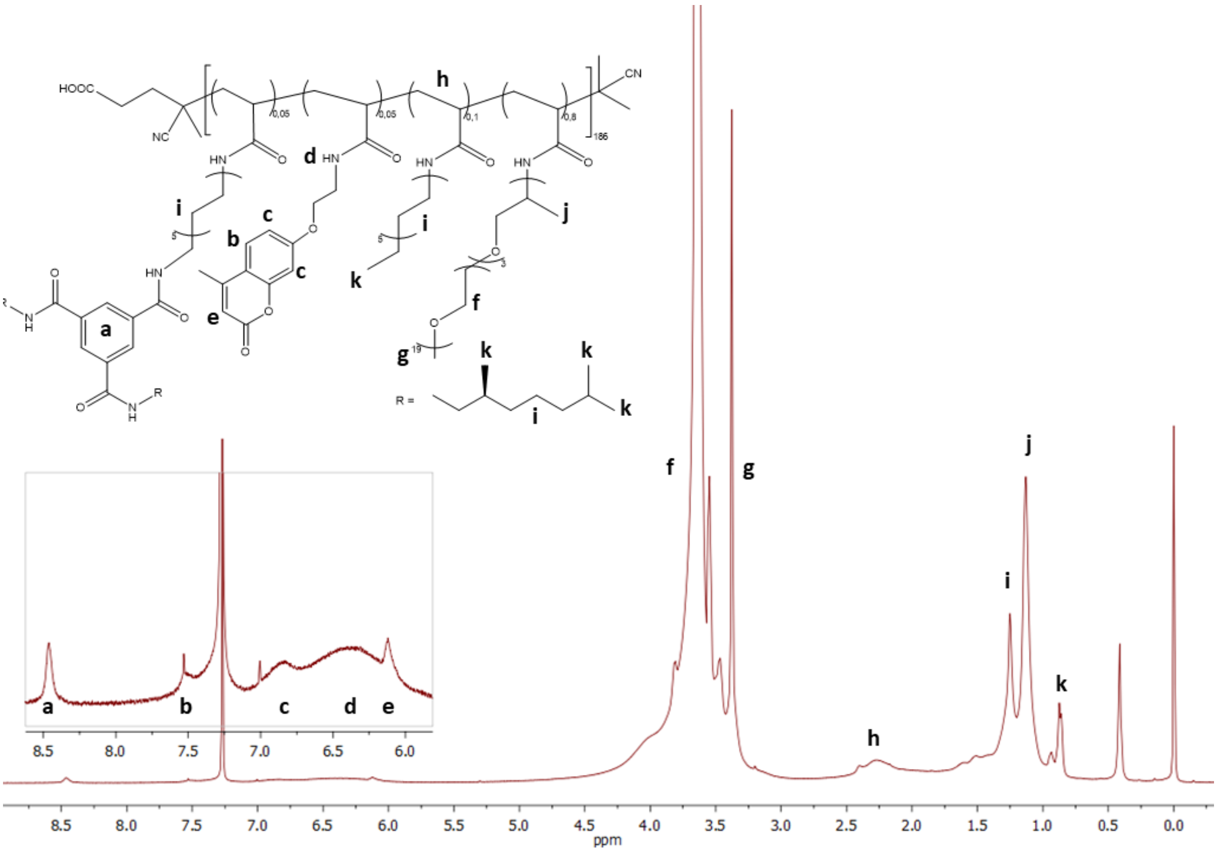

Figure S33: ^{1}H NMR spectrum of **P4** in $CDCl_3$.

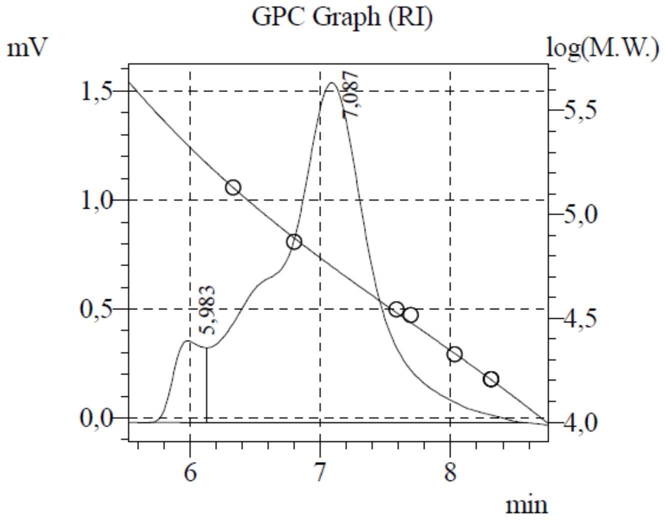

Figure S34: SEC trace of **P4** in DMF.

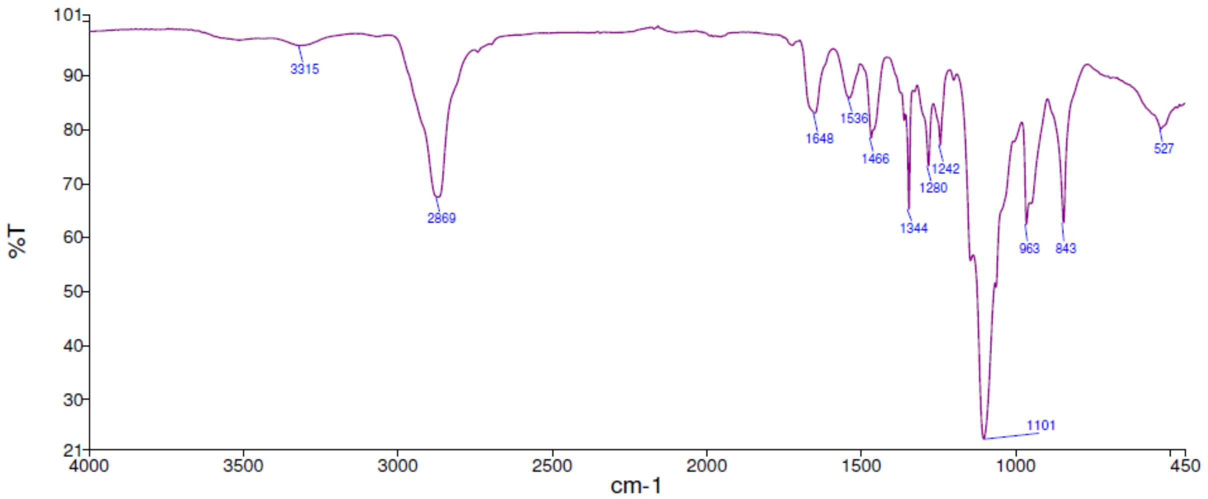

Figure S35: FT-IR spectrum of **P4** in ATR mode.

Synthesis of P5:

^{1}H NMR (399 MHz, $CDCl_3$): δ (ppm) = 8.47 (s), 7.76 – 5.54 (m), 4.51 – 2.71 (m), 2.65 – 0.19 (m). SEC (DMF): M_n = 62 kDa, Đ = 1.53 (RI detector). FT-IR (ATR): n (cm^{-1}) = 3314, 2866, 1725, 1649, 1535, 1455, 1345, 1281, 1243, 1102, 948, 844, 528. Yield = 312 mg (45%).

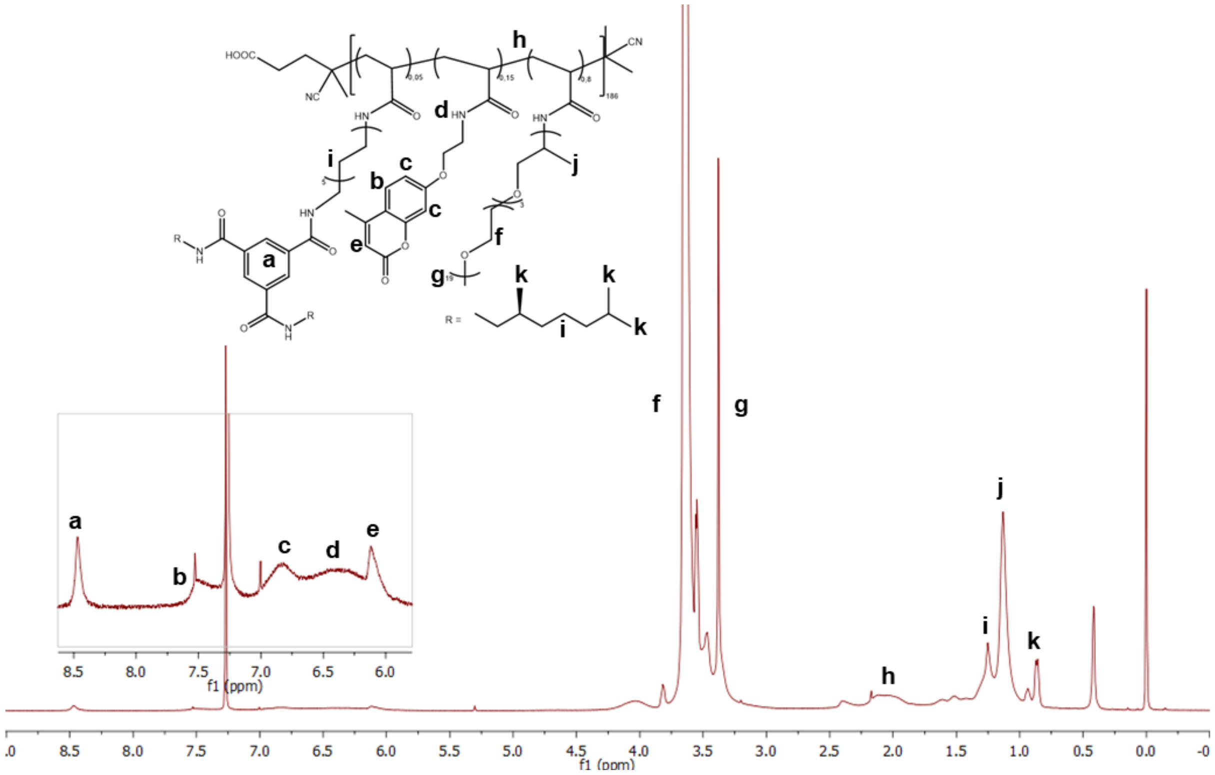

Figure S36: ^{1}H NMR spectrum of **P5** in $CDCl_3$.

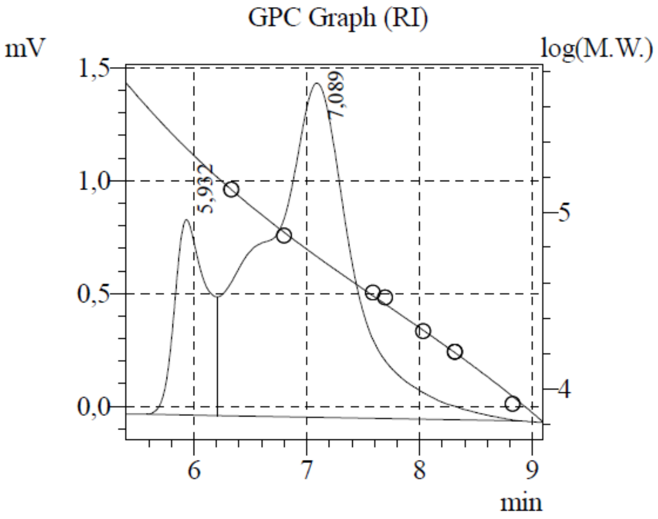

Figure S37: SEC trace of **P5** in DMF.

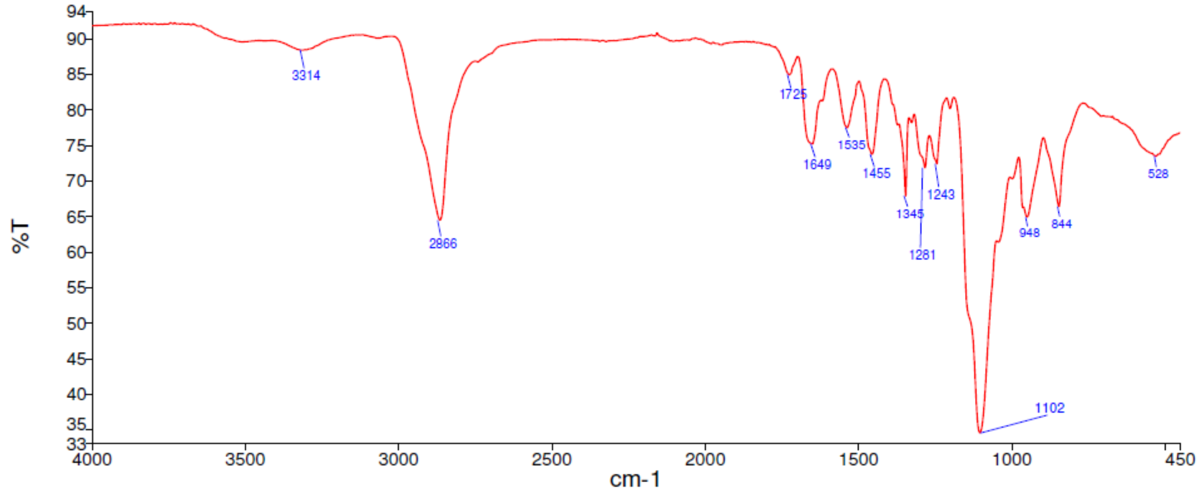

Figure S38: FT-IR spectrum of **P5** in ATR mode.

Synthesis of P6:

^{1}H NMR (399 MHz, $CDCl_3$): δ (ppm) = 8.47 (s), 7.90 – 5.52 (m), 5.16 – 2.80 (m), 2.78 – 0.21 (m). SEC (DMF): M_n = 56 kDa, Đ = 1.32 (RI detector). FT-IR (ATR): n (cm^{-1}) = 3319, 2966, 1725, 1651, 1536, 1455, 1345, 1281, 1243, 1200, 1100, 947, 844, 527. Yield = 419 mg (60%).

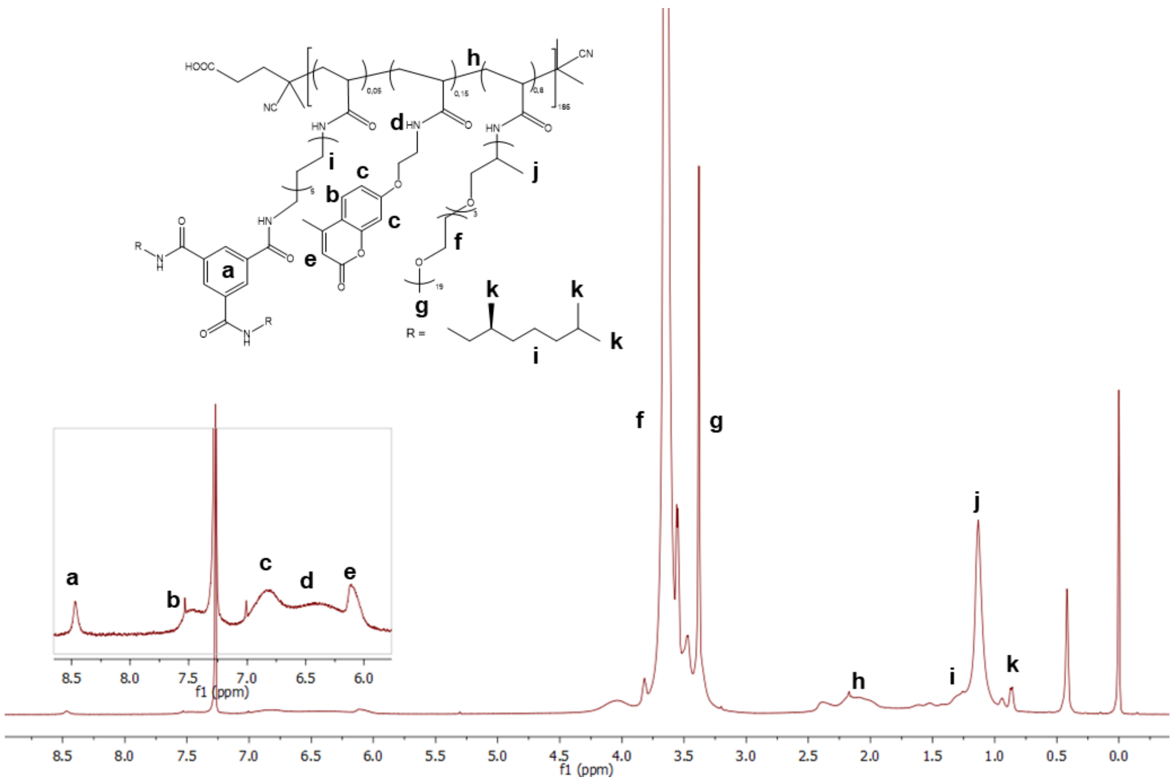

Figure S39: ^{1}H NMR spectrum of **P6** in $CDCl_3$.

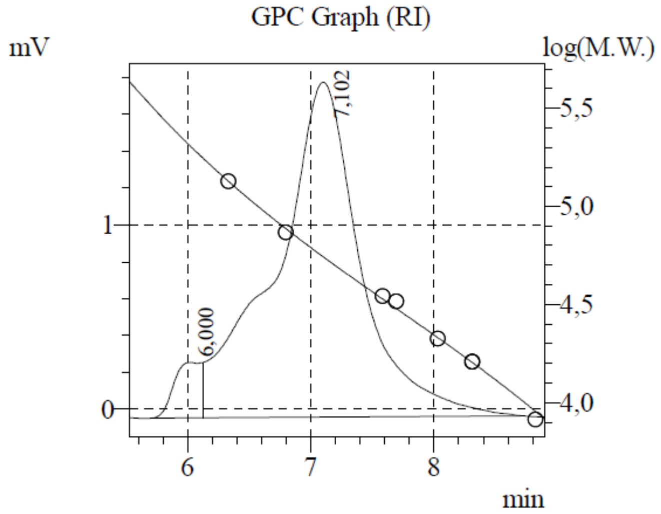

Figure S40: SEC trace of **P6** in DMF.

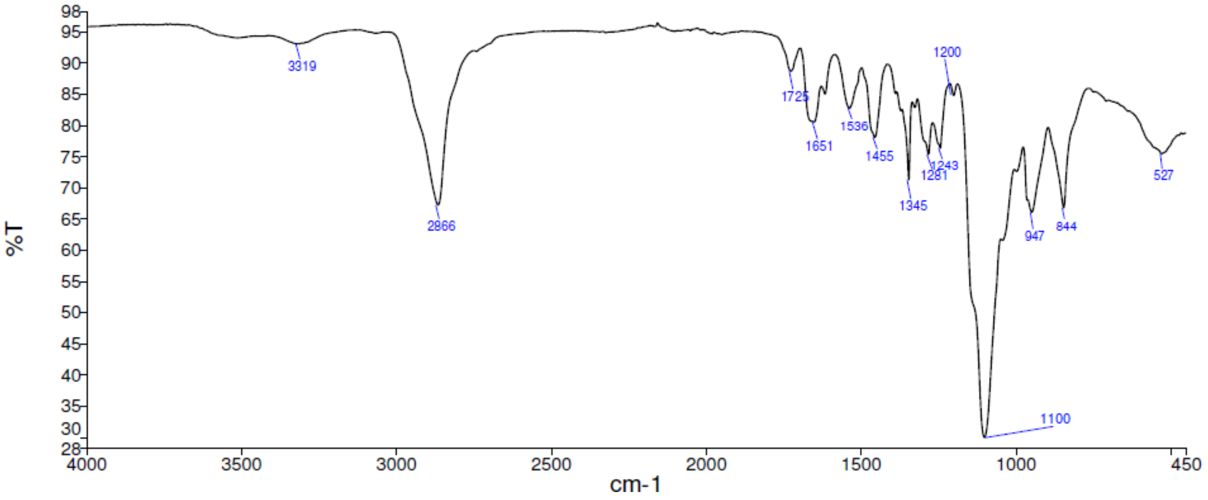

Figure S41: FT-IR spectrum of **P6** in ATR mode.

Synthesis of P7:

^{1}H NMR (399 MHz, $CDCl_3$): δ (ppm) = 6.37 (s), 4.38 – 2.84 (m), 2.13 – 1.55 (m), 1.52 – 0.63 (m). SEC (DMF): M_n = 59 kDa, Đ = 1.32 (RI detector). FT-IR (ATR): n (cm^{-1}) = 2866, 1650, 1536, 1455, 1349, 1297, 1249, 1095, 947, 849, 520. Yield = 327 mg (54%).

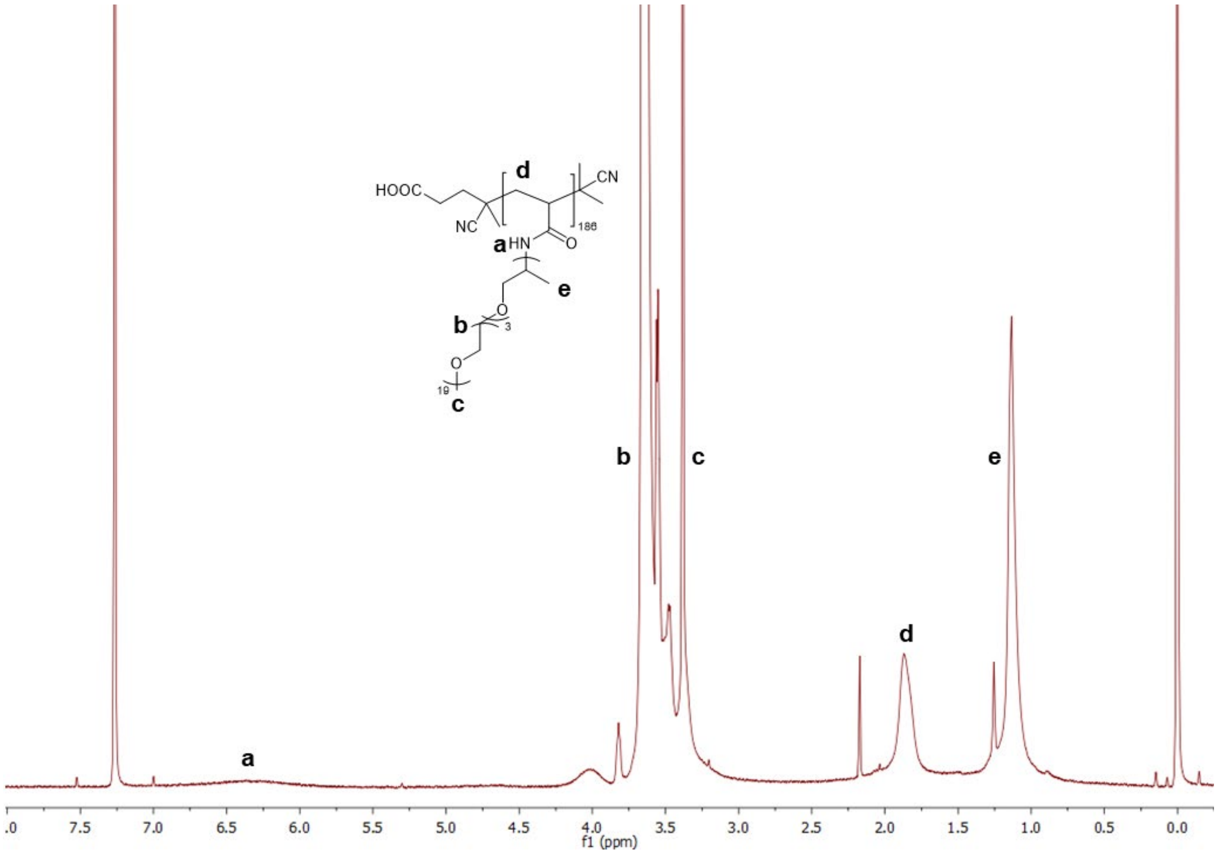

Figure S42: ^{1}H NMR spectrum of **P7** in $CDCl_3$.

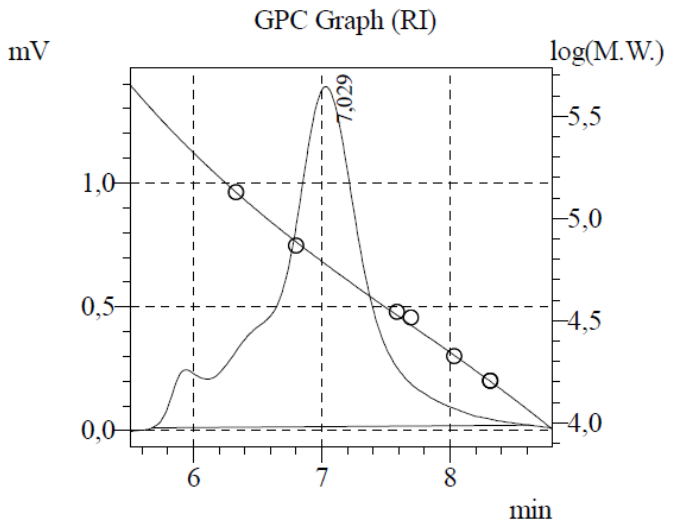

Figure S43: SEC trace of **P7** in DMF.

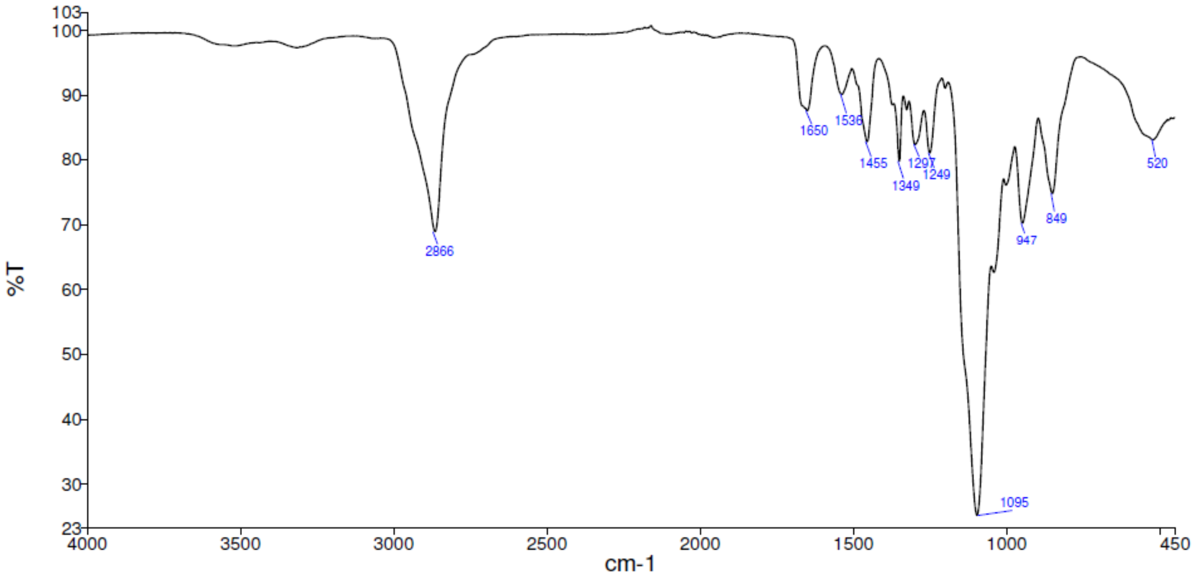

Figure S44: FT-IR spectrum of **P7** in ATR mode.

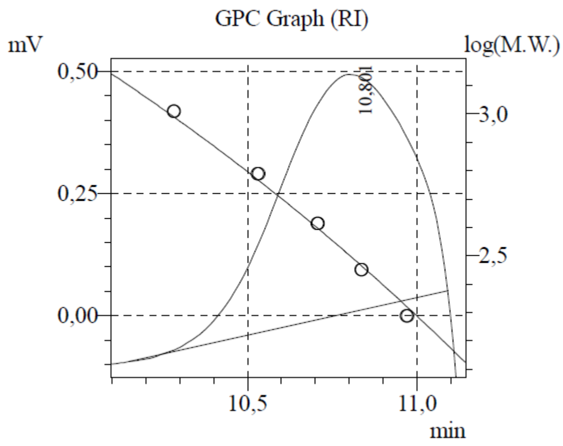

Figure S45: SEC trace of Jeffamine in DMF, absent in **P1** – **P7**.

UV-vis spectrum of P1 – P6:

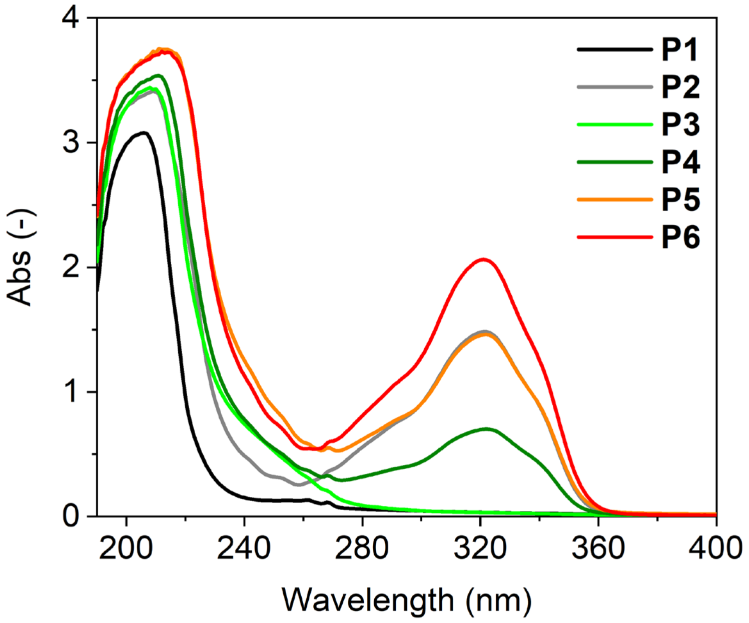

Figure S46: UV-vis absorbance spectra of **P1** – **P6** for 1 mg mL^{-1} solutions in water. The absorption at λ = 320 nm originates from the coumarin monomer graft and nicely follows the incorporation ratio.

^{1}H NMR of P5 in D_2O:

^{1}H NMR (400 MHz, D_2O): δ (ppm) = 8.37 (s), 7.89 – 5.76 (m), 4.32 – 2.64 (m), 2.63 – 0.42 (m).

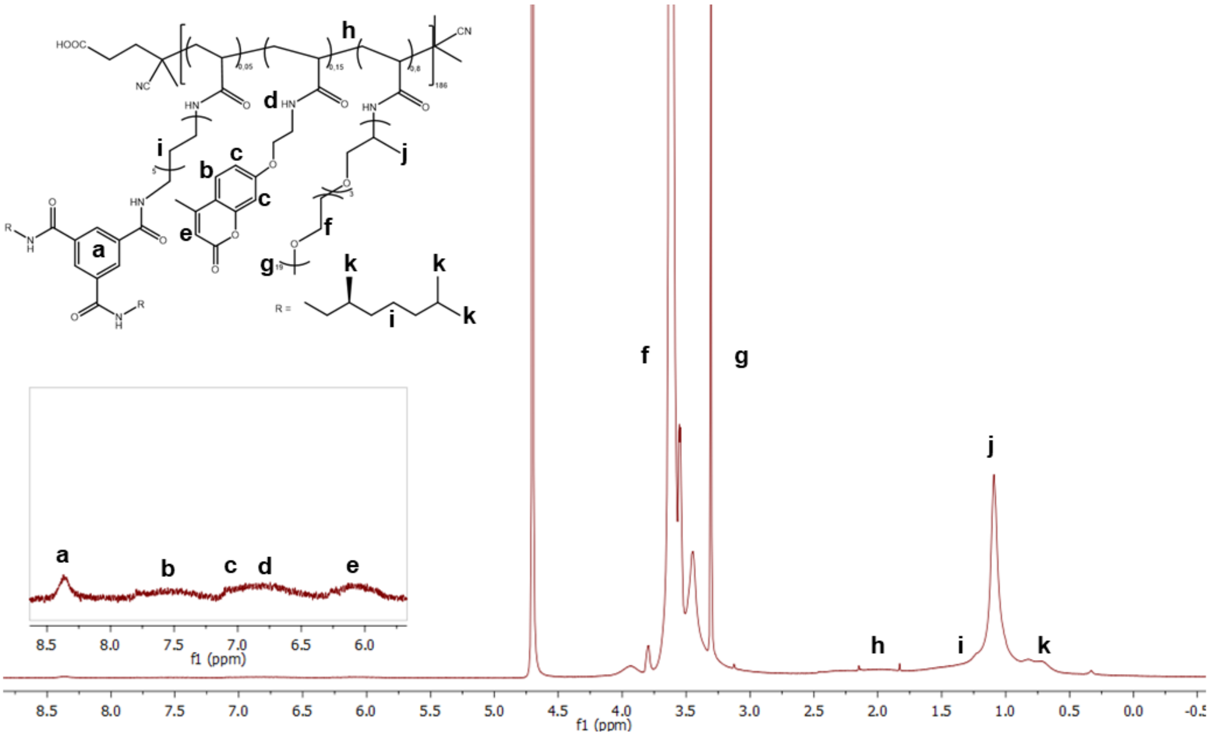

Figure S47: ^{1}H NMR spectrum of **P5** in D_2O.

Nile Red fluorescence in P1 – P7:

The formation of a hydrophobic pocket in **P1** to **P7** upon SCPN formation in water was probed using the hydrophobic dye Nile Red. Due to its solvatochromic nature, the emission maxima of Nile Red will shift depending on its microenvironment. A blueshift corresponds to more hydrophobic environments. Nile Red was added to polymer solutions by injecting from an ethanol stock solution (1 mM) to a final Nile Red concentration of 10 µM, after which the Nile Red emission spectra were recorded. Without polymer, the emission maxima of Nile Red lies at λ_{em} = 656 nm. In **P7**, consisting of only hydrophilic Jeffamine, a small blue-shift to λ_{em} = 652 nm is observed, indicating a very water-rich environment. Hence **P7** does not form a hydrophobic pocket. **P1** and **P2** show the largest blue-shifts, indicating that these polymers contain the most pronounced hydrophobic pockets. Polymers that incorporate coumarin grafts have a less well-defined hydrophobic pocket. For the coumarin bearing polymers, **P4** has the highest blue shift, followed by decreasing blueshifts in **P6, P2**, and **P5**. The observed blueshift is in line with the hydrophobic content of each polymer, where we count dodecyl as one hydrophobic graft, and BTA as three, owing to the three hydrophobic sidechains.

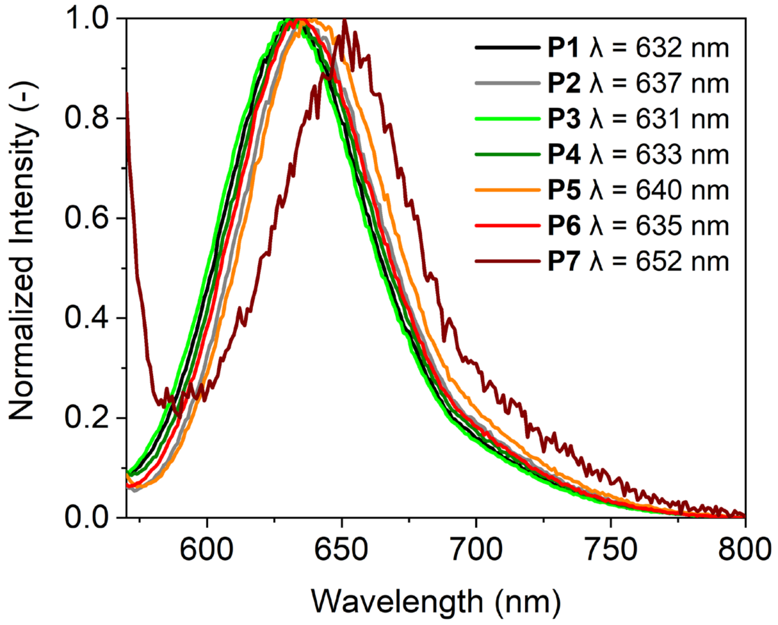

Figure S48: Normalized fluorescence emission spectra of 10 µM Nile Red in 1 mg mL^{-1} of **P1** – **P7** in water. The emission maxima are given in the legend.

CD heating and cooling curves of P3 – P6:

Circular dichroism (CD) heating and cooling curves were measured from 10 to 90 °C by monitoring the Cotton effect at λ = 225 nm and expressed as the molar circular dichroism. All heating curves and cooling curves overlap completely, indicating the reversibility of the BTA self-assembly within the polymers. The strength of the Cotton effect decreases in the order of **P3** to **P6**, corresponding to increasing coumarin content / decreasing hydrophobic content in the polymer series. At 90 °C, more than half of the CD signal is left for **P3** and **P4**, indicating the high stability of these systems. In contrast, **P5** is almost fully disassembled at 90 °C, suggesting that the higher amount of coumarin grafts decreases the temperature stability of the

self-assembled BTAs. **P6** shows only very limited BTA self-assembly.

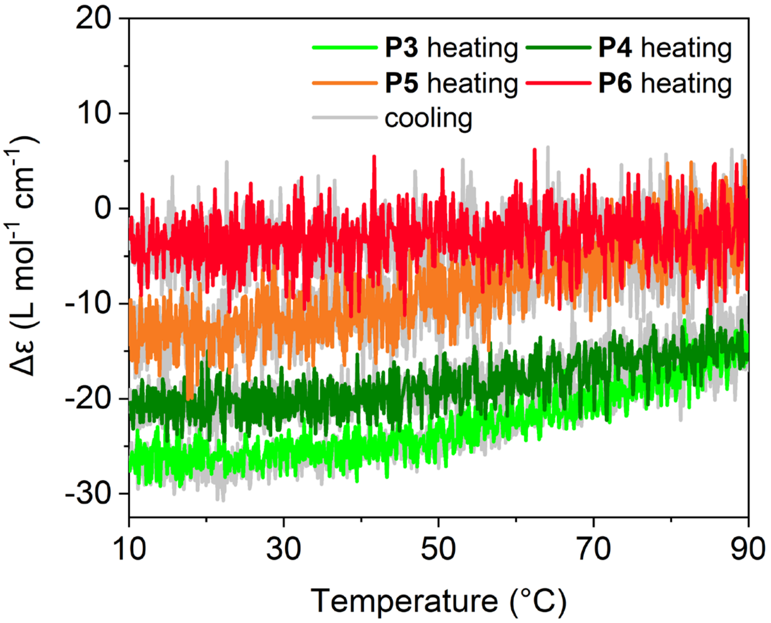

Figure S49: CD heating and cooling curves of **P3** – **P6** in water at a concentration of 1 mg mL^{-1}. c_{BTA} = 41 µM.

4. Sample preparation of polymer solutions

The sample preparation of amphiphilic polymers is incredibly important in order to obtain reproducible particle sizes, especially when incorporating hydrogen-bonding BTA grafts[11]. In this work, aqueous polymer samples were prepared in glass vials from 5 mg mL^{-1} stock solutions in trichloroethylene. After evaporation of the solvent, ultrapure water was added to a polymer concentration of 1 mg mL^{-1} and the polymer was dissolved by vortexing for 20 seconds. Then, the samples were sonicated for 45 minutes using a sonication bath (Branson 2800). Subsequently, the samples were heated in a preheated oven at 90 °C for 45 minutes, allowed to cool in air and equilibrated overnight. All polymer solutions were prepared at a concentration of 1 mg mL^{-1}. This protocol resulted in nanoparticles of reproducible size. For polymer solutions in organic solvent, no sample preparation procedure was performed.

5. Sample filtration

Before cross-linking experiments, DLS measurements, and SEC measurements, samples were filtered to remove dust particles. Aqueous samples were filtered using an Acrodisc 0.2 μm PVDF syringe minispike filter. Samples with organic solvents were filtered using a Whatman 0.2 μm PTFE syringe filter. DMF samples were filtered using a 0.2 μm regenerated cellulose syringe filter. In all cases, the first 3 drops were discarded. A filtration test to check whether the polymer adsorbed to the filter membrane was performed on a 1 mg mL^{-1} **P5** solution in water. The below UV-vis absorbance spectra shows a marginal decrease in the absorbance at λ = 320 nm, which means that only a very small amount of polymer is removed from the solution.

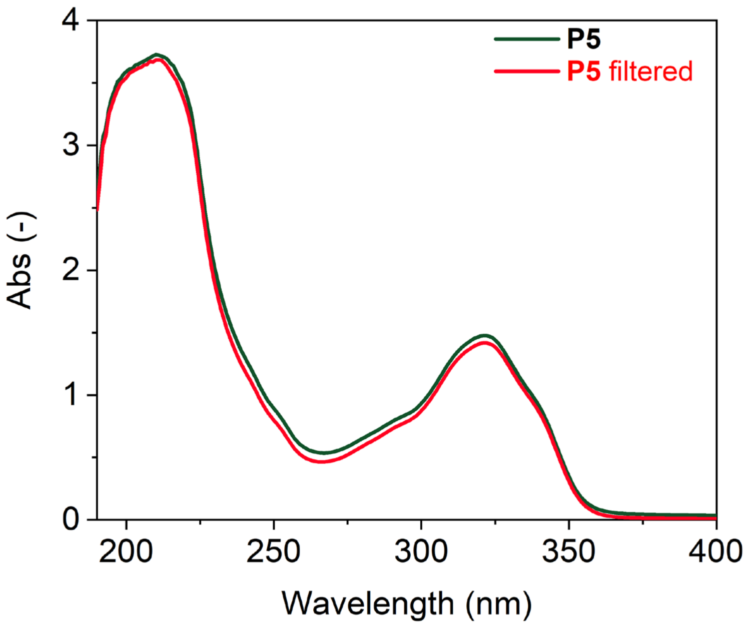

Figure S50: UV-vis absorbance spectra of **P5** in water before and after filtration.

6. Cross-linking setup

3 mL polymer solutions at a concentration of 1 mg mL^{-1} were placed in quartz fluorescence cuvettes (pathlength 1cm x 1cm) and continuously stirred during the cross-linking process. The cuvettes were cooled with an air or nitrogen stream. Cross-linking was performed using a 365 nm mounted led from Thorlabs (M365L2), using the DC4100 driver. The light was focused on the solution using a collimator at a distance of 10 cm. The final cross-linking intensity was 420 mW cm^{-2}, as determined by the RM12 radiometer (Opsitec Dr. Gröbel) using an RM-12 UV-A detector. Cross-linking was performed for a duration of 6 hours.

7. Coumarin cross-linking and spectroscopic properties

The spectroscopic properties of the coumarin dimer were determined by dimerization of 7-ethoxy-4-methyl-coumarin (**3**). Scheme S1 depicts the photodimerization process upon UV-light $\lambda > 300$ nm illumination. The coumarin dimerization is a [2 + 2] cycloaddition between the double bonds next to the lactone moiety,[12–14] forming a cyclobutane ring in the process and resulting in loss of the fluorescent properties.[15] The ring-opening cycloreversion occurs upon $\lambda < 300$ nm illumination.[16,17] Monomer **3** was cross-linked in acetonitrile using a 365 nm led (Thorlabs) and the formed coumarin dimer **4** was isolated using column chromatography. Figures S501 and S52 show the absorbance and fluorescence spectra of a

concentration series of monomer **3** and dimer **4**. The extinction coefficients were determined using the Lambert-Beer law at different wavelengths, summarized in Table S2. The molar absorptivity at $\lambda = 319$ nm of the monomer ($\varepsilon = 13000$ L mol^{-1} cm^{-1}) is a factor 100 higher than that of the dimer ($\varepsilon = 135$ L mol^{-1} cm^{-1}). As the other components of the polymers do not absorb significantly at this wavelength, the absorbance spectra can be used to accurately quantify the monomer conversion. The fluorescence spectra reveal a non-linear trend with concentration, and can therefore not be used for quantification purposes. The small amount of dimer fluorescence measured is believed to correspond to roughly 0.5 mol% monomeric impurity in the dimer sample, which is too low to observe by ^{1}H NMR.

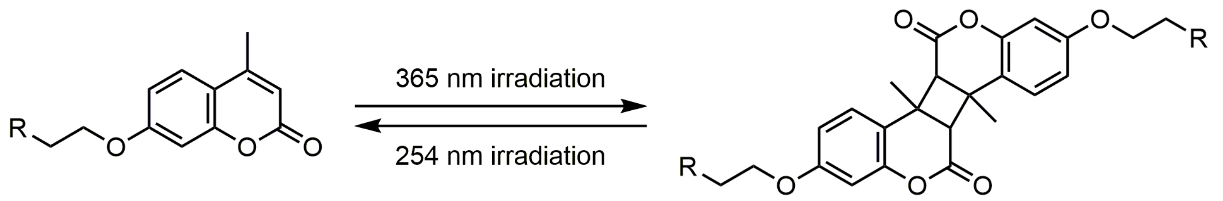

Scheme S1: Cross-linking scheme of 7-ethoxy-4-methyl-coumarin analogues upon irradiation with 365 nm UV-light, or the reverse reaction upon irradiation with 254 nm UV-light. The dimer given here is in the head-to-tail conformation. For the small molecule analogues, R = H; for the polymer series, R = the amide linkage attached to the polymer backbone.

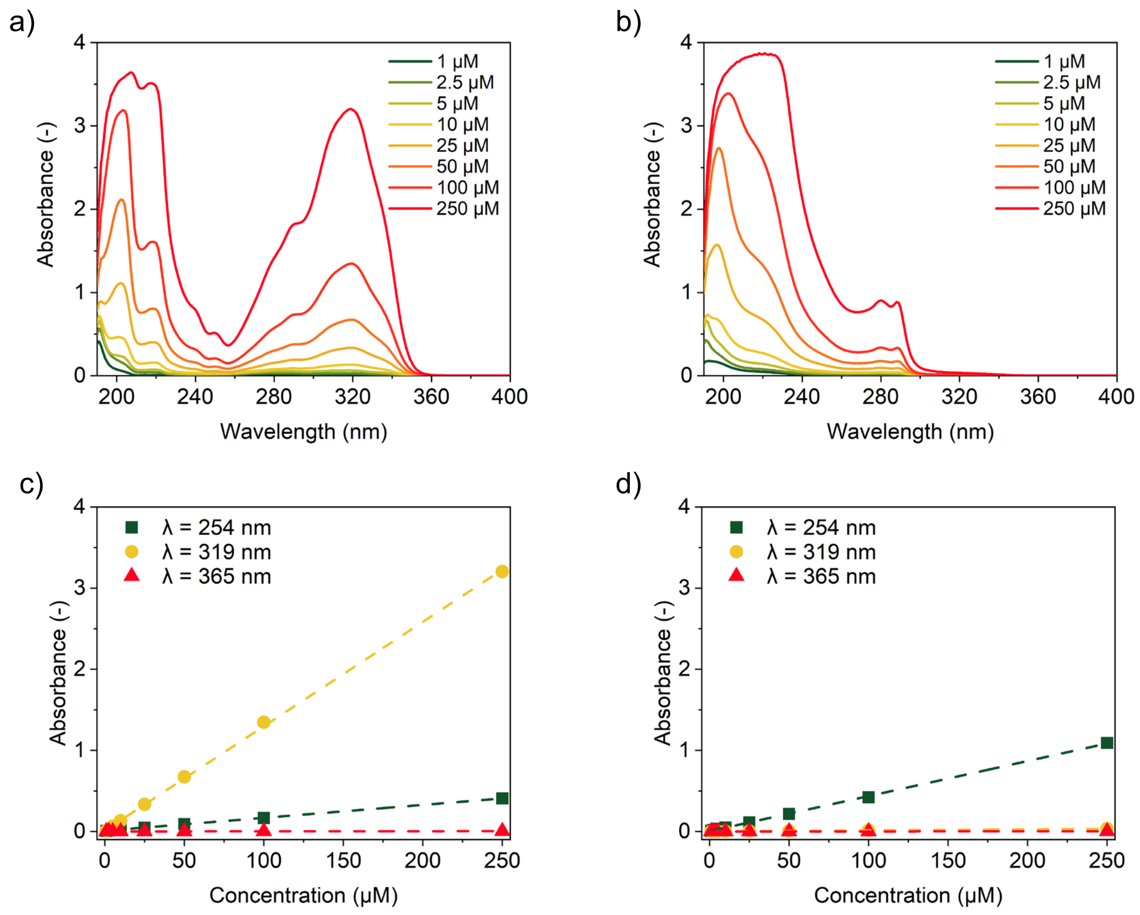

Figure S51: UV-vis absorbance spectra of a concentration series of a) the coumarin monomer **3** and b) the corresponding dimer **4** in acetonitrile. The derived calibration curves for the calculation of the molar absorptivity at different wavelength of c) the coumarin monomer **3** and d) the corresponding dimer **4**. The dashed lines are added to guide the eye.

Table S2: Molar absorptivity ε of compounds **3** and **4** at various wavelengths derived from the concentration series given in Figure S51.

	compound **3**		compound **4**	
λ (nm)	$\varepsilon_{monomer}$ (L mol^{-1} cm^{-1})	R^2	ε_{dimer} (L mol^{-1} cm^{-1})	R^2
254	1600	1.000	4600	1.000
319	13000	0.999	135	0.998
365	15	0.984	7	0.938

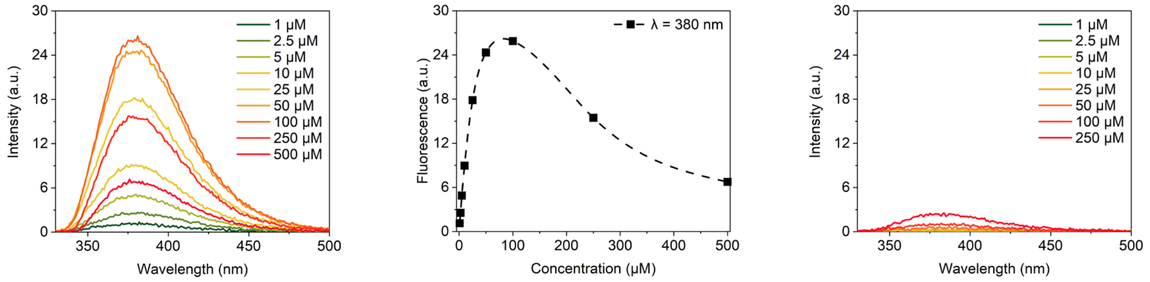

Figure S52: Fluorescence spectra of a concentration series of a) the coumarin monomer **3**, with b) the derived fluorescence at λ = 380 nm as a function of concentration, and c) the corresponding dimer **4** in acetonitrile. The dashed line in b) is added to guide the eye.

8. P4 characterization

CD spectra of P4 in water and THF

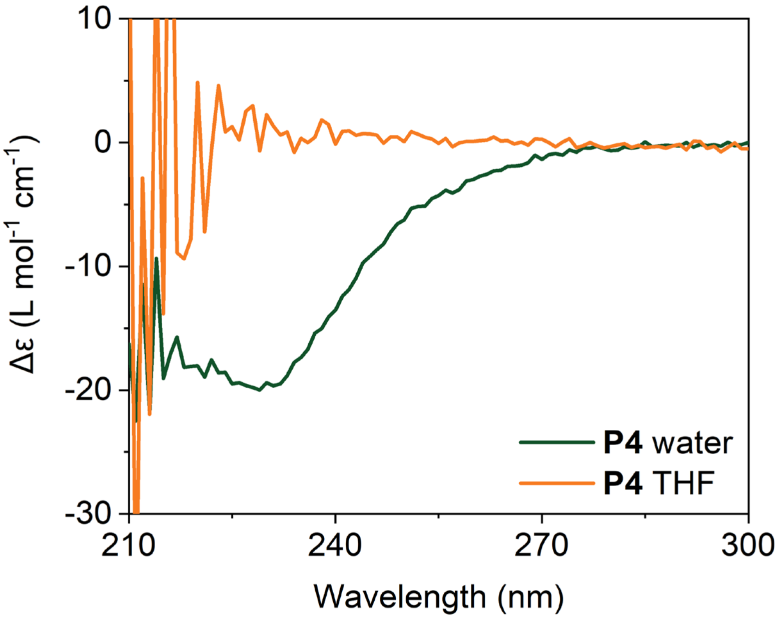

Figure S53: CD spectra of **P4** in water and in THF at a concentration of 1 mg mL^{-1}.

UV-vis absorbance spectra of P4 before and after cross-linking

The conversion of the coumarin monomer to the coumarin dimer can be calculated from the determined molar absorptivity $\varepsilon_{monomer}$ *and* ε_{dimer} at λ = 319 nm using the Lambert-Beer law: $A = \varepsilon cl$, with A the absorbance, c the concentration in M, and l the optical pathlength in cm. l is 1 cm. Before cross-linking (BC), only the coumarin monomer absorbs and thus: $A_1 = \varepsilon_{mono}c_1$, with c_1 the monomer concentration before cross-linking. After cross-linking (AC), the residual monomer and the formed dimer both absorb, and thus: $A_2 = \varepsilon_{monomer}c_2 + \varepsilon_{dimer}(c_1-c_2)/2$, with c_2

the monomer concentration after cross-linking. Using algebra, the monomer conversion can be calculated as:

$$Conversion = (c_1\text{-}c_2)/c_1 \times 100\% = \frac{\varepsilon_{monomer}(A_1-A_2)}{A_1(\varepsilon_{monomer}-0.5\varepsilon_{dimer})} \times 100\% \qquad (1)$$

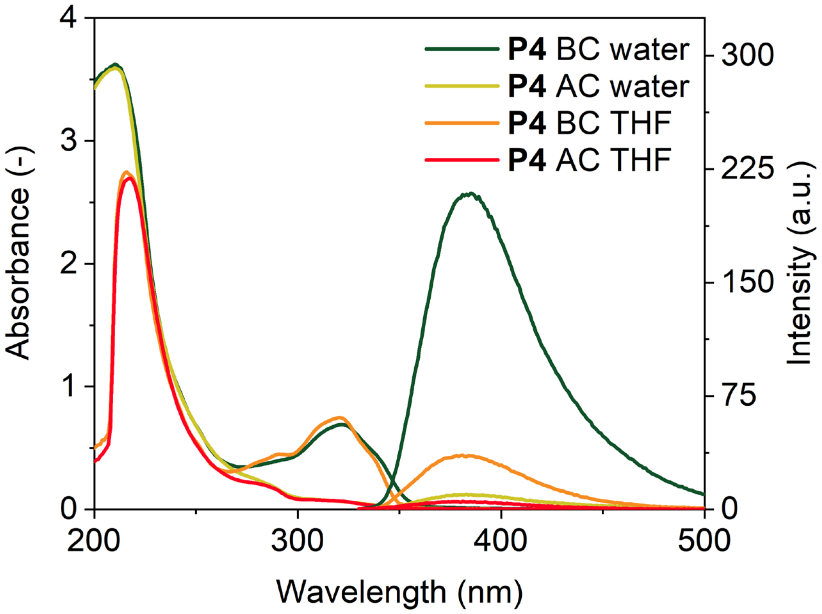

Figure S54: UV-vis absorbance and fluorescence spectra of **P4** before (BC) and after cross-linking (AC) in water or THF.

Table S3: Overview of the calculated coumarin conversion from the absorbance (A) for **P4** cross-linked in water or THF, using equation 1; and fluorescence intensity (I).

**Sample**	**$A_{BC,\,320nm}$ (-)**	**$A_{AC,\,320nm}$ (-)**	**Conversion (%)**	**$I_{BC,\,383nm}$ (a.u.)**	**$I_{AC,\,383nm}$ (a.u.)**	**Change (%)**
**P4 water**	0.68	0.074	91%	221	15	93%
**P4 THF**	0.73	0.057	94%	36	7.6	79%

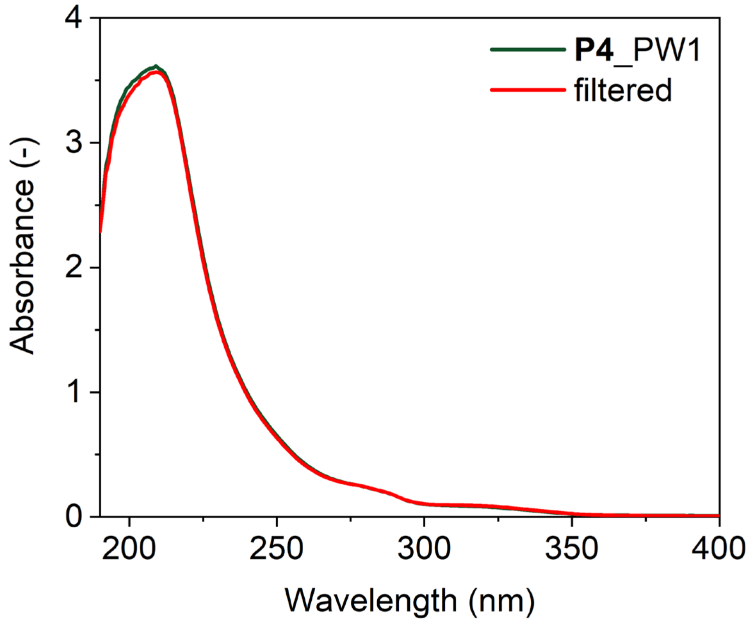

Figure S55: UV-vis absorbance spectra of **P4**_PW1 before (green) and after filtration (red).

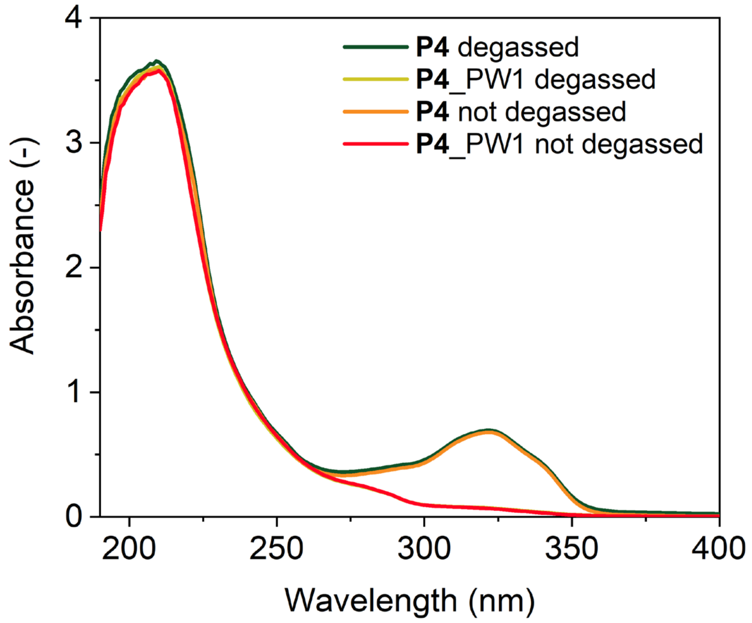

Figure S56: UV-vis absorbance spectra of **P4** and **P4**_PW1 for degassed (argon bubbling) and not degassed solutions.

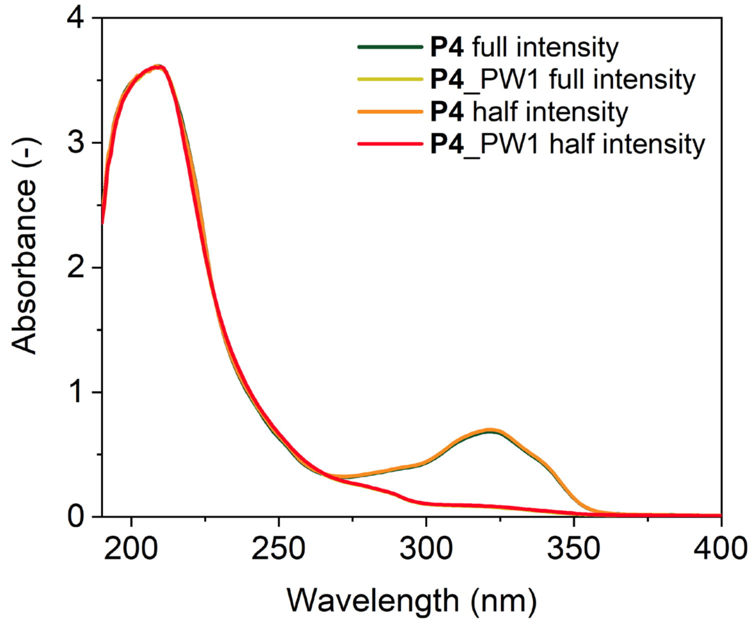

Figure S57: UV-vis absorbance spectra of **P4** and **P4**_PW1 at full and half light intensity.

Light scattering data of P4 before and after cross-linking

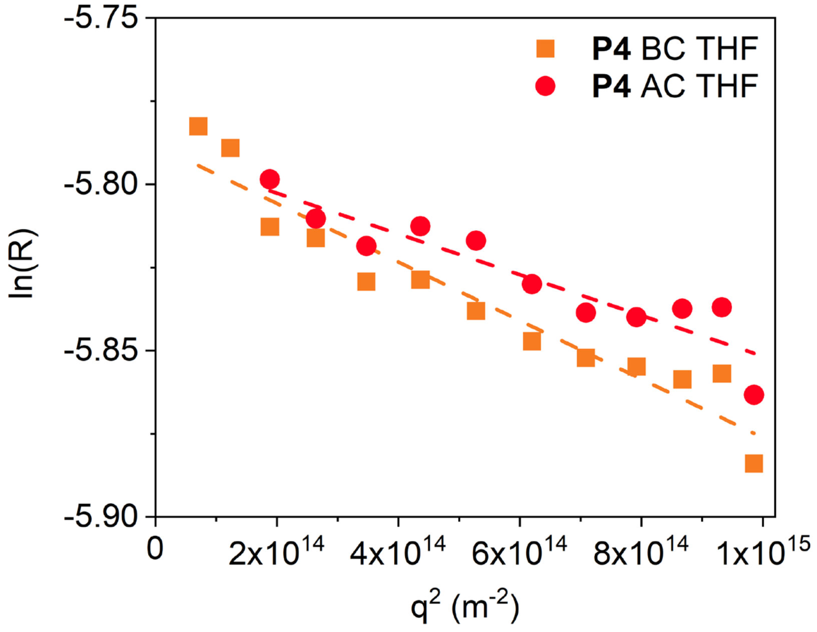

Figure S58: Fit (dashed lines) to the Guinier plot derived from the SLS data obtained for **P4** in THF before (BC) and after cross-linking (AC) at a concentration of 1 mg mL^{-1}.

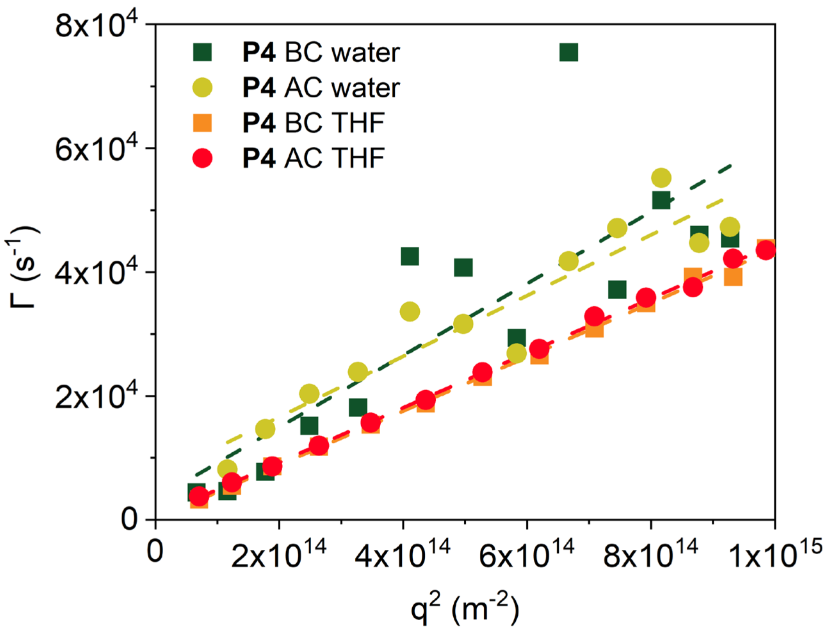

Figure S59: Einstein-Stokes fit (dashed lines) to the DLS data obtained for **P4** in water and THF before (BC) and after cross-linking (AC) at a concentration of 1 mg mL^{-1}.

Table S4 shows the measured hydrodynamic radius R_H of **P4** in the different solvents as mentioned in the main text. R_H was additionally determined by DLS in DMF, which is the hereto standard SEC solvent used to characterize the size of SCPNs. **P4** BC (R_H = 10 - 11 nm) as well as **P4**_PW2 (R_H = 8 – 11 nm) formed particles with much larger R_H in DMF than what is observed in water (R_H = 4 nm), while **P4**_PW1 is the only polymer that retained its small size in PBS and DMF (R_H = 4 nm). Table S5 shows the R_H of **P4**_PW1 and **P4**_PW2 at different temperatures.

Table S4: Overview of hydrodynamic radii (R_H) determined by DLS for **P4,** before and after folding via pathway 1 (PW1) or 2 (PW2) at a concentration of 1 mg mL^{-1}. The resulting solutions are measured in different solvents after a solvent-switch protocol.

**Sample**	**Solvent**	**R_H (nm)**	**Nr. samples**
**P4**	Water	4.3 ± 0.6	6
**P4_PW1**	Water	4.7 ± 0.8	5
**P4_PW1**	Water (redissolved)	4.5	1
**P4_PW2**	Water	15.7 ± 0.8	4
**P4**	THF	10.1 ± 0.2	6

**P4_PW1**	THF	4.7	1
**P4_PW2**	THF	9.8 ± 0.5	5
**P4**	PBS	11.4 ± 0.7	2
**P4_PW1**	PBS	5.1 ± 1.3	2
**P4_PW2**	PBS	8.0 ± 0.8	2
**P4**	DMF (10 mM LiBr)	9.3	1
**P4_PW1**	DMF (10 mM LiBr)	3.4	1
**P4_PW2**	DMF (10 mM LiBr)	9.9	1

Table S5: Overview of hydrodynamic radii (R_H) determined by DLS for **P4**_PW1 and **P4**_PW2 at a concentration of 1 mg mL^{-1}. All solutions are measured in water at 20, 40, and 60 °C.

Sample	Solvent	Temperature (°C)	R_H (nm)	R^2
**P4_PW1**	Water	20	4.2	0.85
**P4_PW1**	Water	40	4.2	0.87
**P4_PW1**	Water	60	4.1	0.91
**P4_PW2**	Water	20	16.0	1.00
**P4_PW2**	Water	40	15.8	1.00
**P4_PW2**	Water	60	16.3	1.00

SEC characterization of P4 and P6 before and after cross-linking

The SEC traces in DMF of **P4** prepared via both pathways are given in Figure S60. Similar to SEC in PBS, **P4**_PW1 has a much higher retention time compared to **P4** and **P4**_PW2, corresponding to a smaller hydrodynamic size for the former. From the overlapping SEC traces, the latter have larger but identical hydrodynamic size. Hence, PW1 results in smaller particles in DMF compared to **P4** and **P4**_PW2, which have identical, larger sizes. The SEC traces in PBS and DMF of **P6** prepared via both pathways are given in Figure S61. **P6** incorporates 15% coumarin grafts, up from 5% in **P4**. The relative trend of the **P6** particle size in PBS for the different pathways is the same as for **P4**. **P6** forms the largest particles, followed by **P6**_PW2, and finally **P6**_PW1 has the smallest particle size. The higher amount of cross-links in **P6** results in a larger shift for **P6**_PW2 than was observed for **P4**_PW2. The trend in relative particle size in DMF is also consistent with the previous **P4** results. Both **P6** and **P6**_PW2 for larger particles with identical size, whereas **P6**_PW1 forms much smaller particles. The obtained apparent molecular weights and dispersity index are given in Table S6.

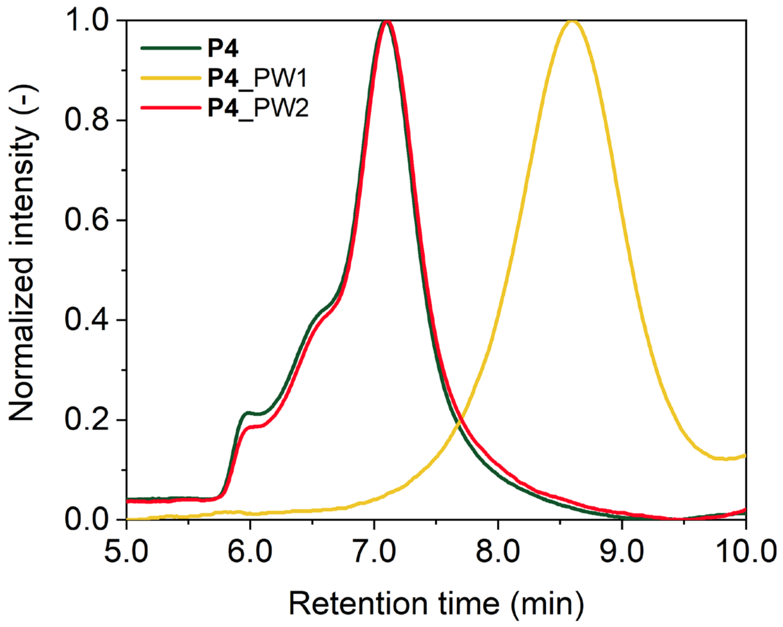

Figure S60: SEC traces in DMF of **P4** before cross-linking, and after folding via pathway 1 (**P4**_PW1) or pathway 2 ((**P4**_PW2) with $c_{polymer}$ = 1 mg mL^{-1}.

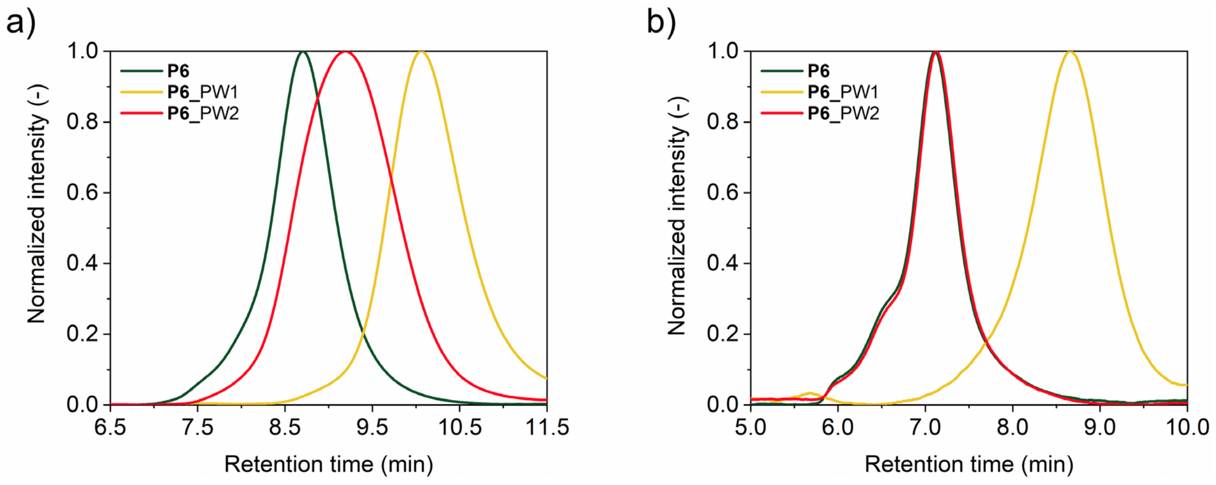

Figure S61: SEC traces in of **P6** before cross-linking, and after folding via pathway 1 (**P6**_PW1) or pathway 2 (**P6**_PW2) in a) PBS and b) DMF (1o mM LiBr) with $c_{polymer}$ = 1 mg mL^{-1}.

Table S6: Overview of apparent molecular weight in kDa and dispersity index Đ as determined by SEC in PBS[a] and DMF[a] for **P4** and **P6** prepared via both pathways. c_{pol} = 1 mg mL^{-1}.

Sample	$M_{n,app}^{PBS}$	$M_{w,app}^{PBS}$	$Đ^{PBS}$ (-)	$M_{n,app}^{DMF}$	$M_{w,app}^{DMF}$	$Đ^{DMF}$ (-)
**P4**	46	65	1.43	56	75	1.35
**P4_PW1**	9.5	12	1.29	11	14	1.26
**P4_PW2**	32	47	1.47	55	72	1.31
**P6**	45	60	1.33	53	66	1.25
**P6_PW1**	9	12	1.30	10	13	1.30
**P6_PW2**	24	35	1.46	51	63	1.24

[a]PBS is 1x PBS with pH = 7.4. DMF contains 10 mM LiBr.

CD characterization of P4 before and after cross-linking

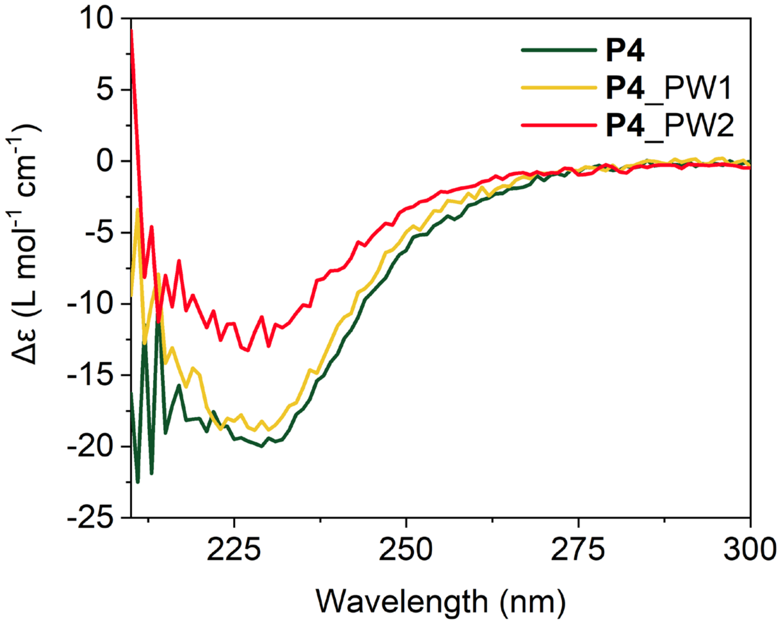

Figure S62: CD spectra of **P4** before and after cross-linking via both pathways. c_{pol} = 1 mg mL^{-1}. c_{BTA} = 41 µM.

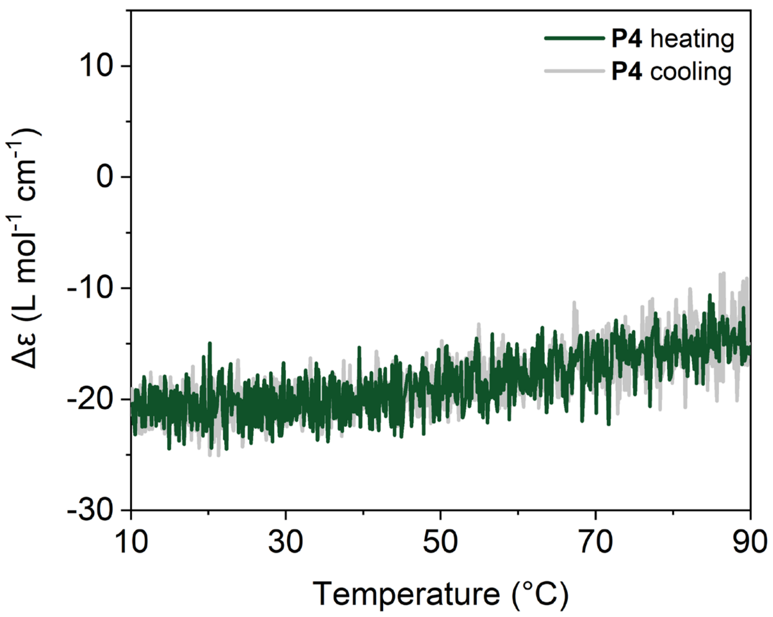

Figure S63: CD heating and cooling curve at λ = 225 nm in water of **P4** before cross-linking. $c_{polymer}$ = 1 mg mL^{-1}, c_{BTA} = 41 µmol.

CD characterization of P4 before and after cross-linking in water / IPA

Figure S64 shows the CD spectra of P4 before cross-linking and after cross-linking via both pathways in different water / isopropanol fractions. The cooling curves recorded at λ = 225 nm are shown in Figure S65. The cooling curves were recorded from 80 °C to 10 °C for different IPA fractions (ϕ_{IPA}) in water, which were then plotted as a function of ϕ_{IPA} per 10 °C in Figure S66. This clearly shows a decrease in the Cotton effect both with increasing ϕ_{IPA} and temperature. To assess the relative strength of the BTA self-assembly between pathways, the CD spectra were normalized between -1 and 0 for **P4**, **P4**_PW1, and **P4**_PW2, where -1 corresponds to the overall lowest measured Cotton effect for that sample (typically at 10 °C and ϕ_{IPA} = 0). Figure 4b in the main text shows the normalized CD signal of **P4**_PW1 and **P4**_PW2, whereas Figure S67 shows the data for **P4**. **P4** and **P4**_PW1 show almost identical behavior as a function of temperature and IPA concentration, suggesting that BTA disassembly is not hindered by the covalent constraints imposed by coumarin dimerization. The high increase at low wavelengths in Figures S64 and S65 were caused by too high sample absorbance.

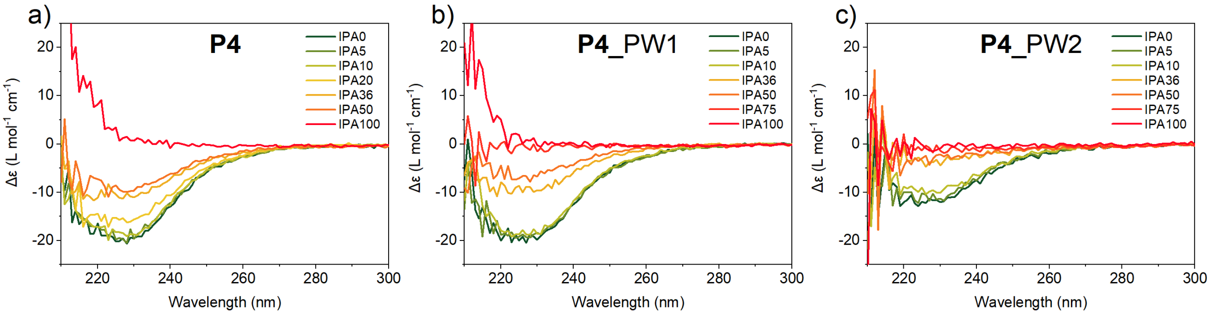

Figure S64: The CD spectra of a) **P4**, b) **P4**_PW1, and c) **P4**_PW2 in different mixtures of Water / isopropanol (IPA). The number following IPA denotes the volume percentage IPA.

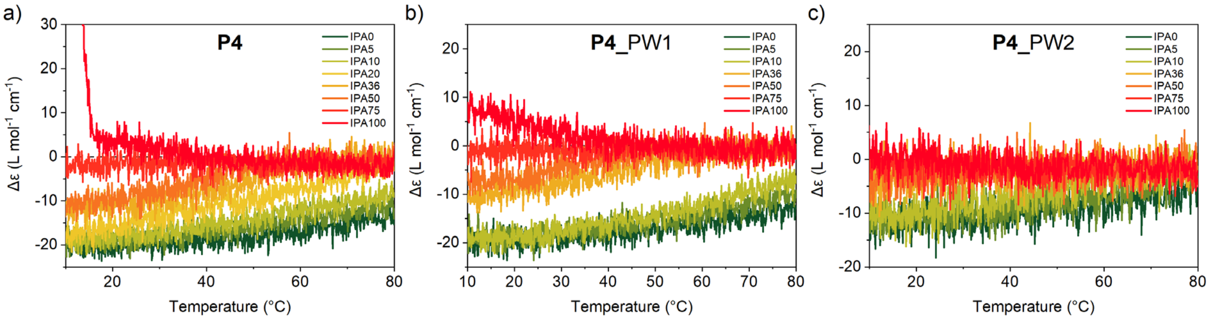

Figure S65: The CD cooling curves of **P4, P4**_PW1, and **P4**_PW2 in different water / IPA mixtures. The number following IPA denotes the volume percentage.

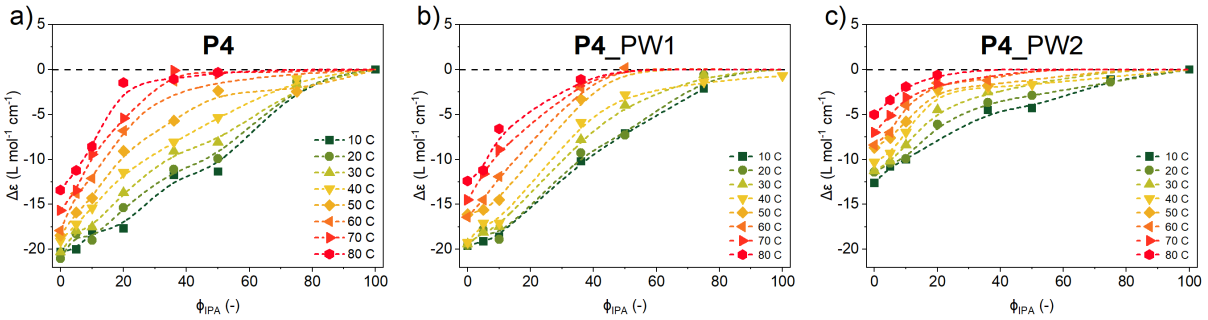

Figure S66: The molar circular dichroism of **P4, P4**_PW1, and **P4**_PW2 as function of IPA fraction in water / IPA mixtures measured at different temperatures. The dashed line is added to guide the eye.

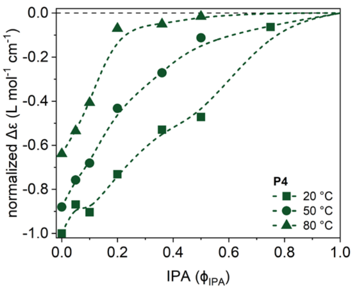

Figure S67: Normalized molar circular dichroism of **P4 o**btained from the CD cooling curves recorded at $\lambda = 225$ nm in water / IPA mixtures as a function of ϕ_{IPA} at different temperatures. The dashed lines are added to guide the eye. $c_{polymer} = 1$ mg mL^{-1}, $c_{BTA} = 41$ µmol.

9. Reverse cross-linking experiments of coumarin

The ring-opening cycloreversion of **4** was carried out by illumination of a 37.5 µM solution of **4** in acetonitrile with a λ = 254 nm lamp (Thorlabs). The absorbance and fluorescence spectra were measured periodically to follow the reverse reaction, shown in Figure S68. **4** was fully converted into **3** after 10 minutes of illumination, as determined from the full recovery of the monomer absorbance and fluorescence. Figure S69 shows the increase of the coumarin monomer absorbance for the ring-opening cycloreversion of **P4**. The change in the shape of the curve below λ = 250 nm is a result of detector saturation due to an increase in sample scattering.

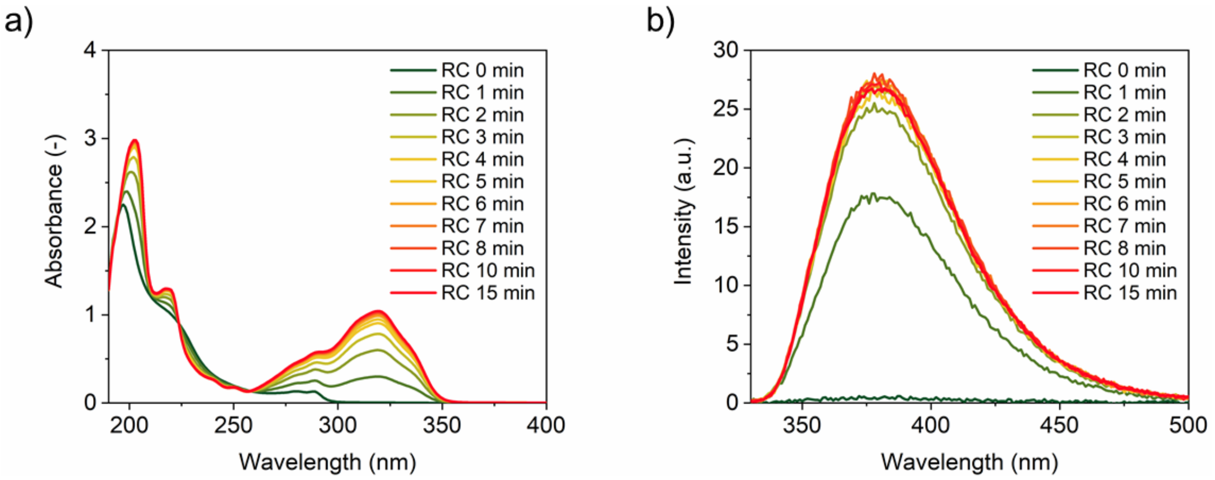

Figure S68: a) UV-vis absorbance spectra and b) fluorescence spectra of the coumarin dimer **4** followed over 15 minutes of reverse cross-linking (RC) into monomer **3**.

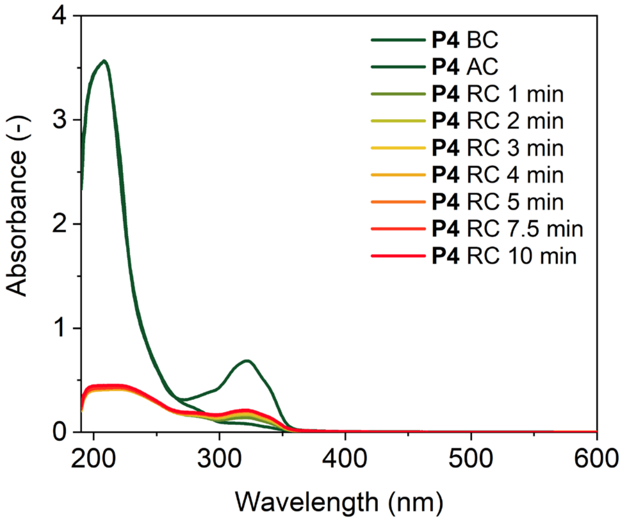

Figure S69: Absorbance spectra of **P4** before (BC) and after cross-linking at 365 nm (AC) via pathway 1, followed over 10 minutes of the ring-opening cycloreversion using UV-light at λ = 254 nm (RC). $c_{polymer}$ = 1 mg mL^{-1}.

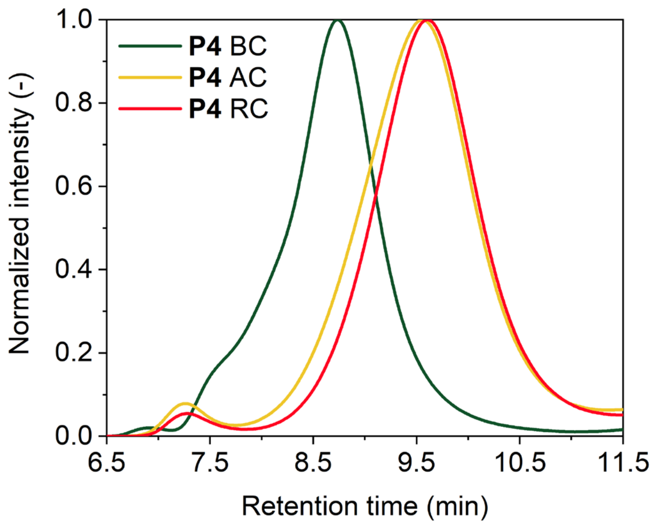

Figure S70: SEC traces of **P4** in PBS before cross-linking (BC), after cross-linking (AC) via pathway 1, and after 10 minutes of ring-opening cycloreversion (RC).

Table S7: Overview of the apparent M_n and M_w in kDa obtained from SEC in PBS for **P4** before cross-linking (BC), after cross-linking (AC), and after ring-opening cycloreversion (RC) for 10 minutes.

**Sample**	$\mathbf{M_{n,app}^{PBS}}$	$\mathbf{M_{w,app}^{PBS}}$	**Đ^{PBS} (-)**
**P4**	56	75	1.35
**P4 AC**	18	25	1.41
**P4 RC**	16	22	1.37

10. Nile Red emission spectra of P4 before and after cross-linking

Figure S71 shows the fluorescence spectra of free Nile Red mixed with **P4**, **P4**_PW1, and **P4**_PW2 in increasingly complex media. NR is shielded inside the hydrophobic pocket of **P4** for all pathways in water, PBS, and DMEM. In 10 vol% FBS in DMEM (FBS-DMEM) however, Nile Red immediately leaches out of the polymer and interacts with the FBS proteins, as evidenced by the observed blue shift. Figure S72 shows the NR fluorescence spectra in the different solvents. The emission maxima are summarized in Table S8. Figure S73 and S74 shows the fluorescence spectra of BTA-NR mixed with **P4**, **P4**_PW1, and **P4**_PW2 in PBS and 20 vol% FBS in PBS (FBS-PBS), respectively, as measured over 3 days. The measured emission maxima over time are summarized in Figure 6a in the main text for **P4**_PW1, and in Figure S75 for **P4** and **P4**_PW2. After 1 h, BTA-NR mixed into **P4** shows a $\lambda_{max,em}$ around 632 nm in both PBS and FBS-PBS. These similar values indicate that the hydrophobic proteins in

FBS do not enter the interior of the SCPN nor do they extract BTA-NR from the particle. For **P4**_PW2, there is a larger difference in $\lambda_{max,em}$ after 1 h between PBS and FBS-PBS, indicating more interaction with the FBS proteins. In FBS, a slow decrease of $\lambda_{max,em}$ is observed over three days, indicating slow exchange dynamics of the BTA-NR present inside the polymers towards the FBS proteins, irrespective of the folding pathway. Figure S76 shows the CD heating and cooling curves of BTA-NR mixed with **P4**, **P4**_PW1, and **P4**_PW2. The good overlap of the heating and cooling curves and agreement with the results obtained for the **P4** systems without BTA-NR indicates the proper mixing of BTA-NR with the BTA stacks in the **P4** systems.

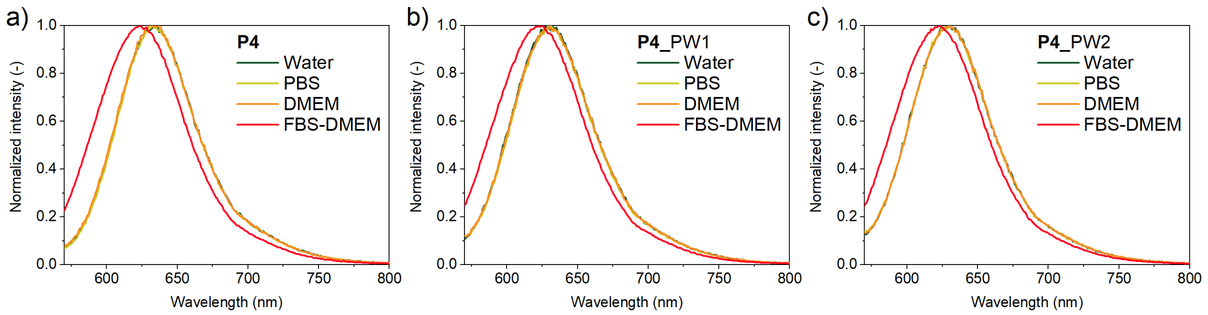

Figure S71: Normalized Nile Red emission spectra of a) **P4**, b) **P4**_PW1, and c) **P4**_PW2 in different media. c_{pol} = 0.2 mg mL^{-1}. c_{NR} = 2 µM.

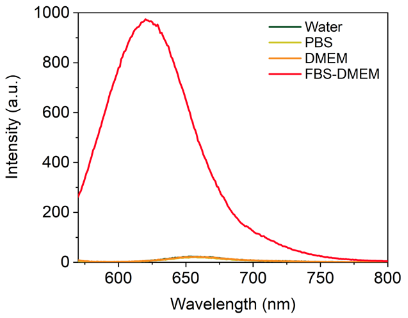

Figure S72: Nile Red emission spectra in the different media. c_{NR} = 2 µM.

Table S8: Nile Red emission maxima in different solvents for **P4**, **P4**_PW1, and **P4**_PW2.

	λ_{Water} (nm)	λ_{PBS} (nm)	λ_{DMEM} (nm)	$\lambda_{FBS\text{-}DMEM}$ (nm)
**Solvent**	656	657	657	622
**P4**	634	634	633	623
**P4_PW1**	630	630	630	623
**P4_PW2**	630	630	630	622

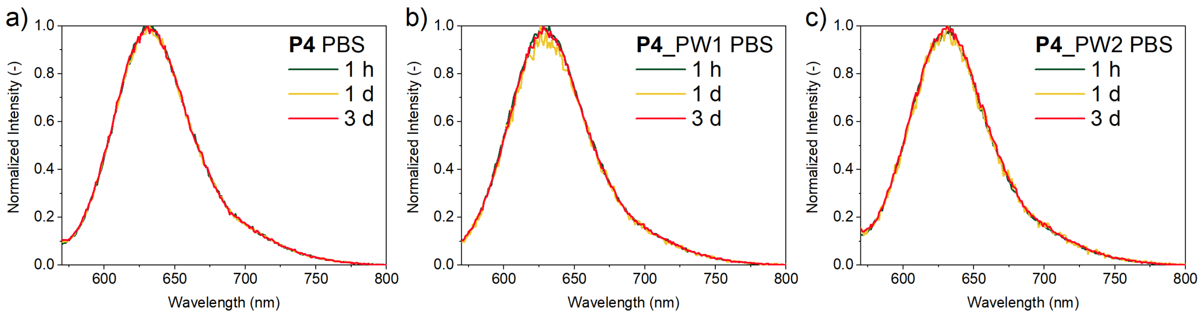

Figure S73: Normalized NR emission spectra of BTA-NR mixed into a) **P4**, b) **P4**_PW1, and c) **P4**_PW2 in PBS measured over three days. c_{pol} = 1 mg mL^{-1}. c_{NR} = 5.55 µM.

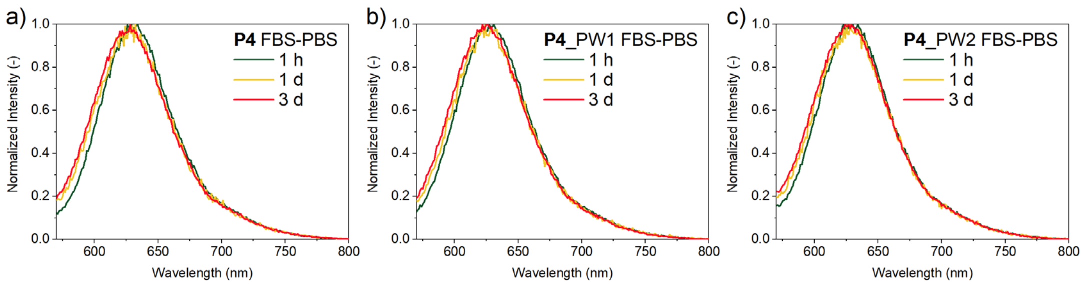

Figure S74: Normalized NR emission spectra of BTA-NR mixed into a) **P4**, b) **P4**_PW1, and c) **P4**_PW2 in FBS-PBS measured over three days. c_{pol} = 1 mg mL^{-1}. c_{NR} = 5.55 µM.

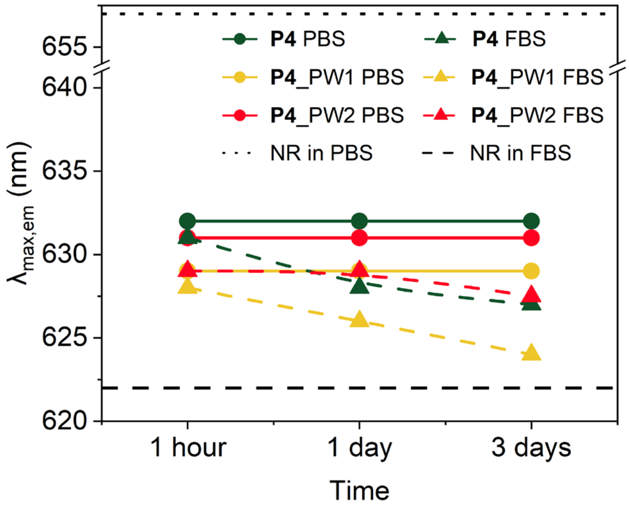

Figure S75: Nile Red fluorescence of **P4**, **P4**_PW1, and **P4**_PW2 samples in PBS and FBS-PBS plotted as the fluorescence maxima λ_{max} of Nile Red and BTA-NR against time. For NR measurements: $c_{polymer}$ = 0.2 mg mL^{-1}, c_{NR} = 2 µM. For BTA-NR measurements: $c_{polymer}$ = 1 mg mL^{-1}, $c_{BTA\text{-}NR}$ = 5.55 µM.

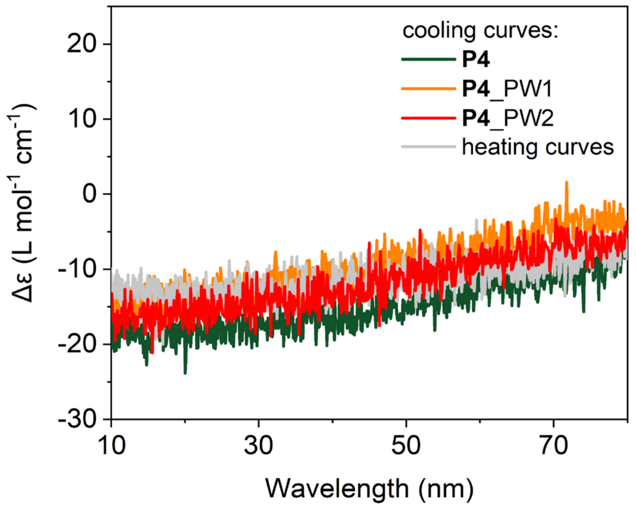

Figure S76: CD heating and cooling curves of **P4**, **P4**_PW1, and **P4**_PW2 mixed with BTA-NR. $c_{polymer}$ = 1 mg mL^{-1}, $c_{BTA\text{-}NR}$ = 5.55 µM. The combined BTA concentration is 47 µM.

11. Nile Red emission spectra of P4 in HeLa cells

Figure S77 shows the fluorescence spectra of NR for **P4** and **P4**_PW1 premixed with BTA-NR in DMEM or after incubation for 24 h with HeLa cells, which resulted in the uptake of the polymers. For both **P4** and **P4**_PW1, a broadening and slight blue shift is observed in HeLa cells compared to DMEM. This blueshift indicates that the BTA-NR experiences a less polar environment, most likely due to interaction of the Nile Red with proteins within the cells. Within the highly competitive environment of living cells, the cross-links provided by PW1 does not stabilize the SCPN conformation and the BTA-NR experiences the same microenvironment as **P4**. The observed blueshift reported here for **P4** and **P4**_PW1 reveals similar stability in HeLa cells for BTA-NR to what we have observed for polymers incorporating 20% dodecyl grafts or 5% BTA + 15% dodecyl grafts where the Nile Red was covalently attached to the polymer backbone, while showing better stability compared to polymers incorporating lower amounts of hydrophobic grafts.[2]

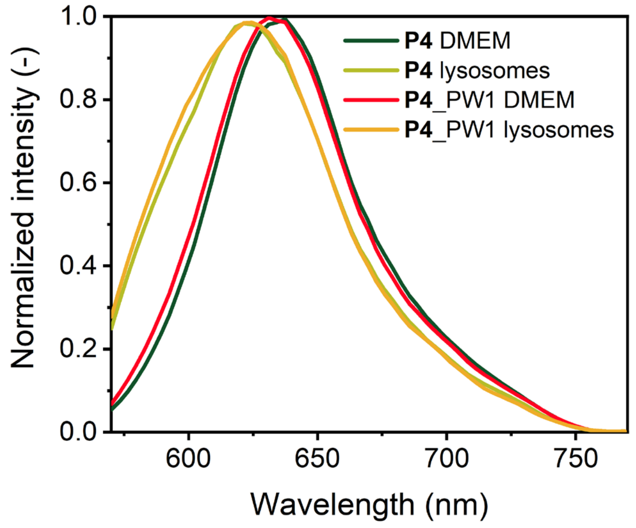

Figure S77: Nile Red fluorescence of **P4** and **P4**_PW1 samples mixed with BTA-NR in DMEM and inside the HeLa cells extracted from confocal microscopy. $c_{polymer}$ = 1 mg mL^{-1}, $c_{BTA\text{-}NR}$ = 5.55 µM.

12. References

(1) Liu, Y.; Pauloehrl, T.; Presolski, S. I.; Albertazzi, L.; Palmans, A. R. A.; Meijer, E. W. Modular Synthetic Platform for the Construction of Functional Single-Chain Polymeric Nanoparticles: From Aqueous Catalysis to Photosensitization. *J. Am. Chem. Soc.* **2015**, *137* (40), 13096–13105. https://doi.org/10.1021/jacs.5b08299.

(2) Deng, L.; Albertazzi, L.; Palmans, A. R. A. Elucidating the Stability of Single-Chain Polymeric Nanoparticles in Biological Media and Living Cells. *Biomacromolecules* **2021**. https://doi.org/10.1021/acs.biomac.1c01291.

(3) Jeffery, K.; Gareth, M.; Nicholas, Y.; Hank, P. Benzopyrane and Imidazole Derivatives Useful for the Stabilization of Lization and Amyloidogenic Immunoglobulin Light Chains. WO2020US25607 20200329, 2020.

(4) *CRC Handbook of Chemistry and Physics, 88th Edition*; Lide, D. R., Ed.; CRC Press: Boca Raton, FL, 2007.

(5) Brandrup, J.; Immergut, E. H.; Grulke, E. A.; A. Abe; Bloch, D. R. *Polymer Handbook*, 3rd ed.; Wiley: New York, 1989.

(6) Brandrup, J.; Immergut, E.; Grulke, E. A. *Polymer Handbook*, Vol. 12.; Wiley-Interscience, 1990.

(7) Polymer Source Inc. Poly(ethylene glycol) methyl ether, (initiator: methoxyethanol-based), P10558-EGOCH3 https://www.polymersource.ca/index.php?route=product/category&path=2_2183_15_9

5_526&subtract=1&categorystart=A-1.1.1.10&serachproduct=yes (accessed Apr 4, 2022).

(8) Cooper, J. R.; Dooley, R. B. *Release on the IAPWS Formulation 2008 for the Viscosity of Ordinary Water Substance*; Berlin, Germany, 2008.

(9) Kinart, C. M.; Kinart, W. J.; Ćwiklińska, A. 2-Methoxyethanol-Tetrahydrofuran-Binary Liquid System. Viscosities, Densities, Excess Molar Volumes and Excess Gibbs Activation Energies of Viscous Flow at Various Temperatures. *J. Therm. Anal. Calorim.* **2002**, *68* (1), 307–317. https://doi.org/10.1023/A:1014981921097.

(10) *Burdick & Jackson Solvent Guide*, 3rd ed.; Burdick & Jackson Laboratories: Muskegon, MI, 1990.

(11) Ter Huurne, G. M.; De Windt, L. N. J.; Liu, Y.; Meijer, E. W.; Voets, I. K.; Palmans, A. R. A. Improving the Folding of Supramolecular Copolymers by Controlling the Assembly Pathway Complexity. *Macromolecules* **2017**, *50* (21), 8562–8569. https://doi.org/10.1021/acs.macromol.7b01769.

(12) Chen, Y.; Hong, R.-T. Synthesis of Polyesters Containing Coumarin Dimer Components by Photopolymerization of 7,7′-Coumarinyl Polymethylene Dicarboxylates. *J. Polym. Res.* **1994**, *1* (3), 285–293. https://doi.org/10.1007/BF01374553.

(13) Seoane Rivero, R.; Bilbao Solaguren, P.; Gondra Zubieta, K.; Gonzalez-Jimenez, A.; Valentin, J. L.; Marcos-Fernandez, A. Synthesis and Characterization of a Photo-Crosslinkable Polyurethane Based on a Coumarin-Containing Polycaprolactone Diol. *Eur. Polym. J.* **2016**, *76*, 245–255. https://doi.org/10.1016/j.eurpolymj.2016.01.047.

(14) Jivaramonaikul, W.; Rashatasakhon, P.; Wanichwecharungruang, S. UVA Absorption and Photostability of Coumarins. *Photochem. Photobiol. Sci.* **2010**, *9* (8), 1120–1125. https://doi.org/10.1039/c0pp00057d.

(15) He, J.; Tremblay, L.; Lacelle, S.; Zhao, Y. Preparation of Polymer Single Chain Nanoparticles Using Intramolecular Photodimerization of Coumarin. *Soft Matter* **2011**, *7* (6), 2380–2386. https://doi.org/10.1039/c0sm01383h.

(16) Chang, H.; Shi, M.; Sun, Y.; Jiang, J. Photo-Dimerization Characteristics of Coumarin Pendants within Amphiphilic Random Copolymer Micelles. *Chinese J. Polym. Sci. (English Ed.* **2015**, *33* (8), 1086–1095. https://doi.org/10.1007/s10118-015-1657-4.

(17) Ciamician, G.; Silber, P. Chemische Lichtwirkungen. *Berichte der Dtsch. Chem. Gesellschaft* **1902**, *35* (4), 4128–4131. https://doi.org/10.1002/cber.19020350450.
